# Turn-on Fluorescent Biosensors for Imaging Hypoxia-like Conditions in Living Cells

**DOI:** 10.1021/jacs.2c01197

**Published:** 2022-04-29

**Authors:** Santiago Guisán-Ceinos, Alexandra R. Rivero, Fernando Romeo-Gella, Silvia Simón-Fuente, Silvia Gómez-Pastor, Natalia Calvo, Alejandro H. Orrego, José Manuel Guisán, Inés Corral, Francisco Sanz-Rodriguez, Maria Ribagorda

**Affiliations:** † Departamento de Química Orgánica, Facultad de Ciencias, Universidad Autónoma de Madrid, 28049 Madrid, Spain; ‡ Departamento de Química, Facultad de Ciencias, Universidad Autónoma de Madrid, 28049 Madrid, Spain; § Departamento de Biología, Facultad de Ciencias, Universidad Autónoma de Madrid, 28049 Madrid, Spain; ∥ Departamento de Biocatálisis, Instituto de Catálisis y Petroquímica (CSIC), Campus UAM, 28049 Madrid, Spain; ⊥ Institute for Advanced Research in Chemical Sciences (IAdChem), Universidad Autónoma de Madrid, 28049 Madrid, Spain

## Abstract

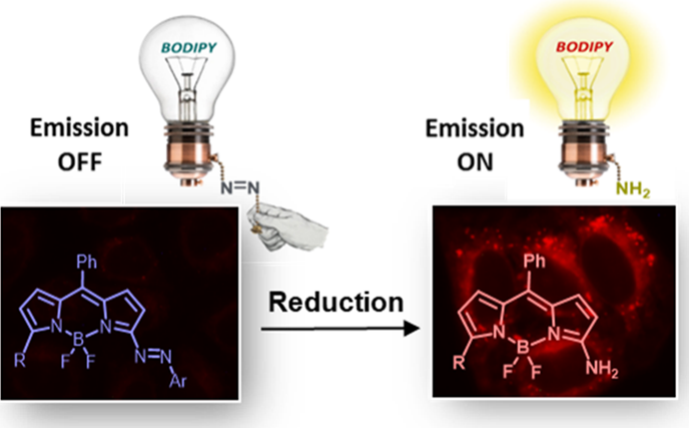

We present the synthesis, photophysical properties, and biological application of nontoxic 3-azo-conjugated BODIPY dyes as masked fluorescent biosensors of hypoxia-like conditions. The synthetic methodology is based on an operationally simple N=N bond-forming protocol, followed by a Suzuki coupling, that allows for a direct access to simple and underexplored 3-azo-substituted BODIPY. These dyes can turn on their emission properties under both chemical and biological reductive conditions, including bacterial and human azoreductases, which trigger the azo bond cleavage, leading to fluorescent 3-amino-BODIPY. We have also developed a practical enzymatic protocol, using an immobilized bacterial azoreductase that allows for the evaluation of these azo-based probes and can be used as a model for the less accessible and expensive human reductase NQO1. Quantum mechanical calculations uncover the restructuration of the topography of the S_1_ potential energy surface following the reduction of the azo moiety and rationalize the fluorescent quenching event through the mapping of an unprecedented pathway. Fluorescent microscopy experiments show that these azos can be used to visualize hypoxia-like conditions within living cells.

## Introduction

Azobenzenes have emerged as outstanding fluorescent quenchers used for biosensing and bioimaging studies.^1,2^ Following its coupling, the azo moiety is able to extinguish the emission of several fluorophores by a nonradiative process operating under the Förster resonance energy transfer (FRET) mechanism or by a non-FRET process generally associated with the ultrafast trans/cis N=N bond isomerization.^3^ The fluorescence of these azo-based quenching dyes can be activated by chemical or biological reductants (*i.e.*, azoreductases)^4^ that trigger the cleavage of the N=N azo moiety, releasing the fluorescent probe and allowing for the easy tracking of biological events by fluorescence microscopy.^5^ The reversibility of the first half-reduction of the azo N=N double bond is highly dependent on the oxygen in the medium. Thus, in reducing media and in the absence of oxygen, such as hypoxia-like conditions, the azo compounds can suffer an irreversible reductive breakdown of the N=N bond, releasing the fluorophore. Nagano *et al.*(6) originally reported a nonemissive fluorophore probe based on the azo Black Hole Quencher (BHQ-3) for the detection of acute ischemic and related hypoxia diseases.^7,8^ From this seminal work, a series of azocompounds, conjugated with other fluorophores, have been reported to inhibit different emission wavelengths and to detect different levels of hypoxia and acute ischemia.^9−11^ Although the use of azocompounds as fluorescent quenchers is well known, to the best of our knowledge, there has been no attempt to conjugate an azo moiety to a BODIPY by the C-3 position. This simple azo quencher would be useful for the development of novel sensors for hypoxia-like conditions because reductive cleavage of the azo bond would re-establish the outstanding emission properties of the BODIPY^12^ moiety.

**Figure 1:**
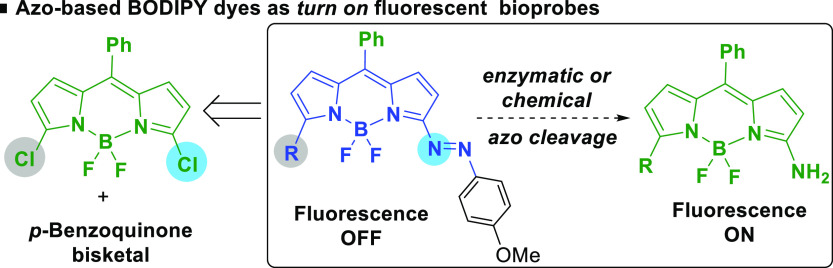
Design of nonfluorescent 3-azo-BODIPY and fluorescent 3-amino-BODIPY dyes.

Herein, we report the synthesis of 3-arylazo-BODIPY, prepared under very mild conditions from 3,5-dichloro-BODIPY and commercially available *p*-benzoquinone bisketal (Figure 1). The initial nonemissive azo-conjugated BODIPY accommodates a chloro substituent, amenable for chemical modification, providing a handle for further functionalization and modulation of the final photophysical properties. The fluorescence is turned on under chemical or enzymatic (*i.e.*, azoreductase) reductive conditions and also visualized in HeLa cells under hypoxia-like conditions by fluorescence imaging techniques. Quantum mechanical calculations provide a novel insight into the molecular origin of the quenching event and rationalize the photophysical properties of both azo dyes and the released amino fluorophores.

## Results and Discussion

**Scheme 1:**
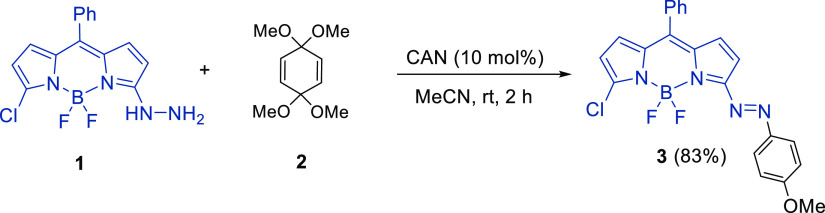
Synthesis of Azo-BODIPY Dye **3** from **1**

Generally, azo-based nonemissive fluorophores are prepared by azo-coupling reactions, between *in situ* generated aryldiazonium salts and electron-rich aromatic compounds, or by a Mills reaction, from nitroso derivatives and aromatic amines. In a previous work, we reported the synthesis of azo derivatives based on reactions of arylhydrazines with quinone bisketals.^13^ In contrast to conventional methods known for making azo derivatives, this strategy avoids using strong acids or base reagents and only requires catalytic amounts of cerium ammonium nitrate (CAN).^14,15^ To examine whether our methodology could be extended to the preparation of more complex heterocyclic (BODIPY) azo derivatives, we studied the reaction between hydrazine-BODIPY **1** and the commercially available bis-dimethyl acetal of *p*-benzoquinone **2**. To our delight, the reaction using CAN as the catalyst at rt gave the desired azo derivative **3** as a nonfluorescence dark blue solid in 83% isolated yield (Scheme 1).

**Scheme 2:**
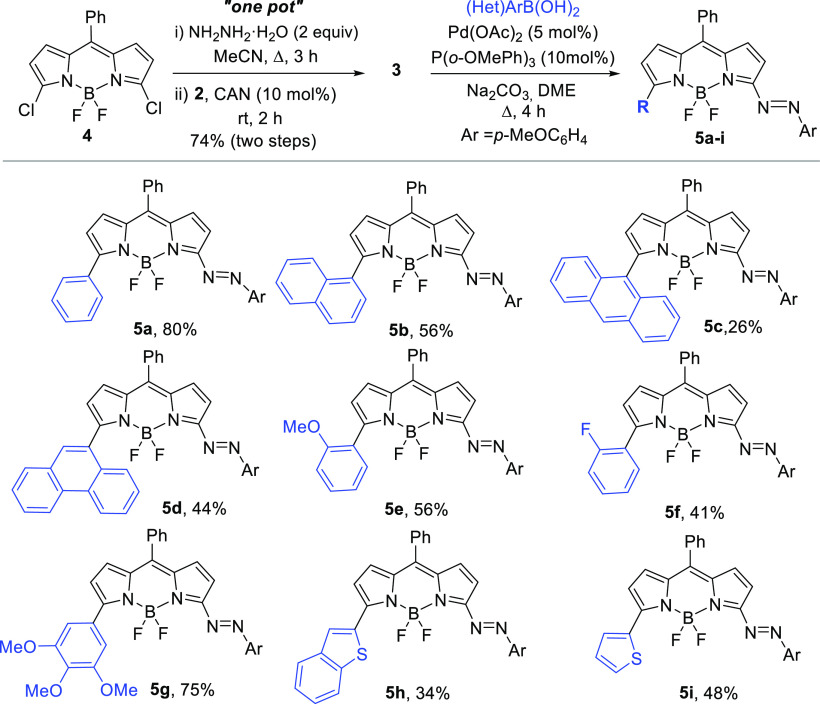
One-Pot Synthesis of **3** and Preparation of **5**

Gratifyingly, the method was improved following an operationally simple two-step strategy, starting from 3,5-dichloro-BODIPY^16^**4** by the sequential addition of hydrazine followed by the addition of **2** and CAN. This procedure avoided the isolation of hydrazine-**1** and decreased the amount of hydrazine required for its preparation^17^ (Scheme 2). Notably, the remaining 5-chloro substituent allowed to increase the chemical diversity and electronic properties accessible by a Suzuki cross-coupling reaction (Scheme 2).^18^

The feasibility of the cross-coupling reaction was initially studied using 5-chloro-3-arylazo-BODIPY **3** and phenyl boronic acid. After screening different palladium salts and phosphine ligands,^19^ the best yield was obtained using Pd(OAc)_2_ as the catalyst, P(2-MeOPh)_3_ as the ligand, and Na_2_CO_3_ as the base in DME at reflux for 4 h,^20^ isolating **5a** in 80% yield.

Following these conditions, several aryl and heteroaryl groups were engaged. Condensed aromatic boronic acids, such as naphthyl, anthracenyl, and phenantryl, afforded the desired compounds. Electron-rich and electron-poor aryl boronic acids and heterocyclic 2-benzothienyl and 2-thienyl derivatives were also well tolerated and gave the desired products in moderate to good yields. All these new 3-arylazo-BODIPY derivatives were dark blue solids, lacking fluorescence emission.

**Scheme 3:**
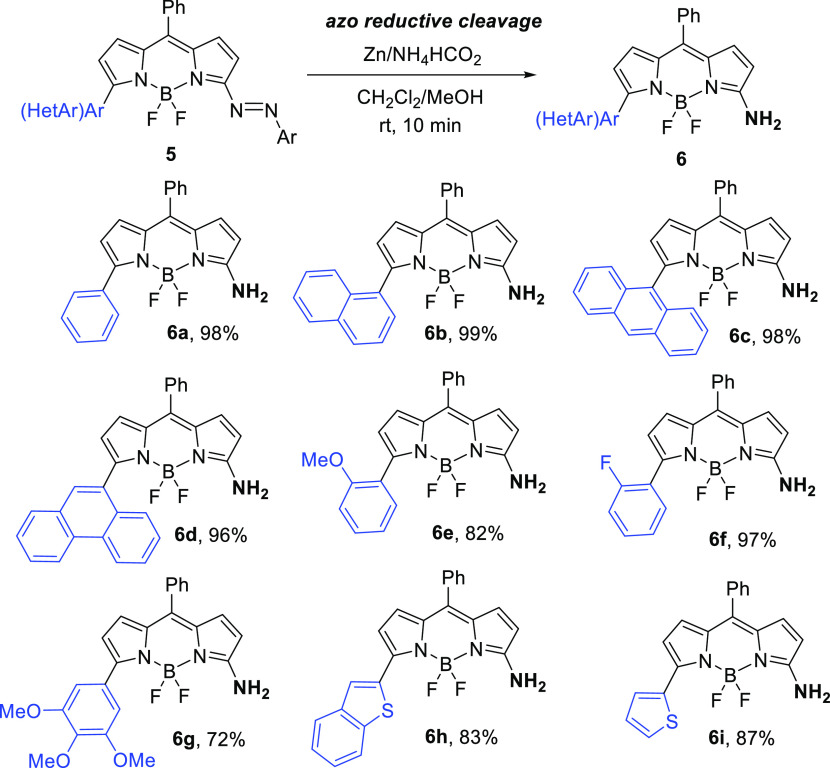
Synthesis of 3-Amino-BODIPY Dyes **6**

To examine the potential of azo-BODIPY **5** as a fluorescent turn-on probe under reductive conditions, we initially undertook the reductive azo bond cleavage by treating **5a–i** with zinc and ammonium formate (Scheme 3). In all cases, the corresponding 3-amino-derivatives **6a–i** were successfully formed in excellent yields. A remarkable color change was observed almost immediately, from an initially dark blue solution to a fluorescent green to orange-yellow solution.

**Figure 2:**
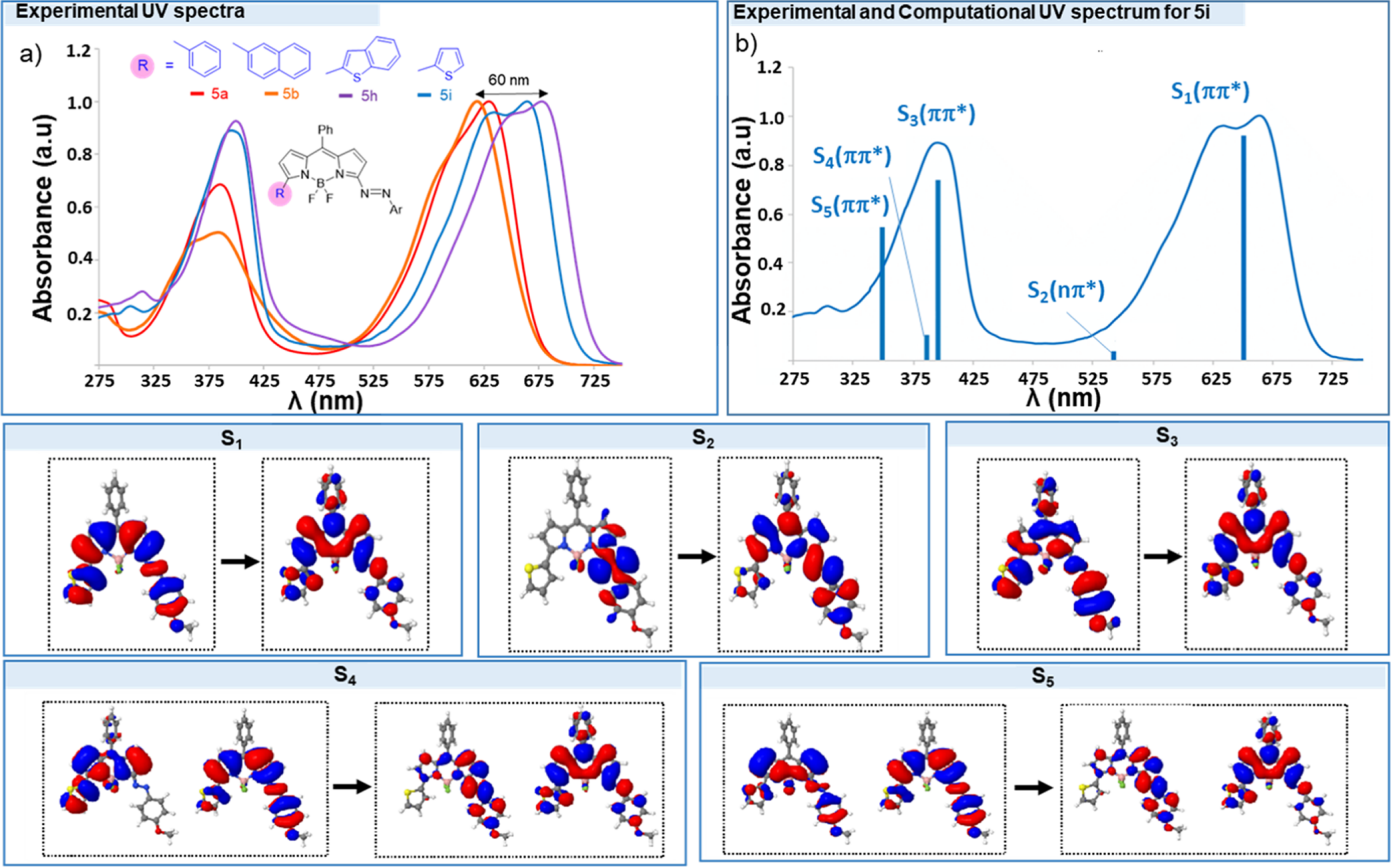
(Top) (a) Experimental absorption spectra of representative **5a,b** and **5h,i** derivatives in DCM. (b) RI-ADC(2)/def2-SVP vertical excitation energies superimposed to the experimental *trans*-azo **5i** absorption spectra (red-shift applied 0.38 eV). (Bottom) Natural transition orbitals for the lowest-lying excited electronic states S_1_–S_5_ contributing to the main absorption bands of **5i**.

Next, we studied the photophysical properties of the 3-arylazo-BODIPY **5** and 3-amino-BODIPY **6** using CH_2_Cl_2_ as the solvent (see Tables S2 and S3). The UV spectra of representative examples (**5a,b** and **5h,i**) are shown in Figure 2 (top a). The absorption spectra of **5** correspond to a red-shifted version of the combined chromophores (BODIPY and azobenzene). In general, two absorptions bands appear at 619–679 nm and in the UV region (∼370–400 nm). The maximum absorption band can be shifted up to 60 nm depending on the C-5 substituent. The thienyl derivatives **5h** (λ_abs_ 679 nm) and **5i** (λ_abs_ 665 nm) displayed the most red-shifted bands. As expected, azo-BODIPY **5** showed negligible fluorescence emission (see Figure S3 for a representative emission spectra of **5i**).

A detailed analysis of the nature of the two characteristic absorption bands on the most stable *trans*-azo isomer of **5i**, based on the scrutiny of the natural transition orbitals, reveals that the electronic transitions in these systems cannot be longer ascribed either to the BODIPY or the azobenzene moiety separately, but instead both fragments are involved in the electronic excitation; see Figure 2 (top b and bottom). In particular, the first absorption at 630–700 nm, which is assigned to the S_1_, corresponds to a ππ* transition localized along the BODIPY and azo aryl moieties. We ascribe the second absorption at 370–380 mainly to the ππ* S_3_ and S_5_ states, also delocalized on the BODIPY and the azobenzene moieties, although the predominant contribution in the case of S_3_ and S_5_ is from the azobenzene fragment and the BODIPY, respectively (see Figures 2b and S6). Our calculations predict the characteristic nπ* transition of the azobenzene as the dark state peaking at *ca.* 467 nm (S_2_).

**Figure 3:**
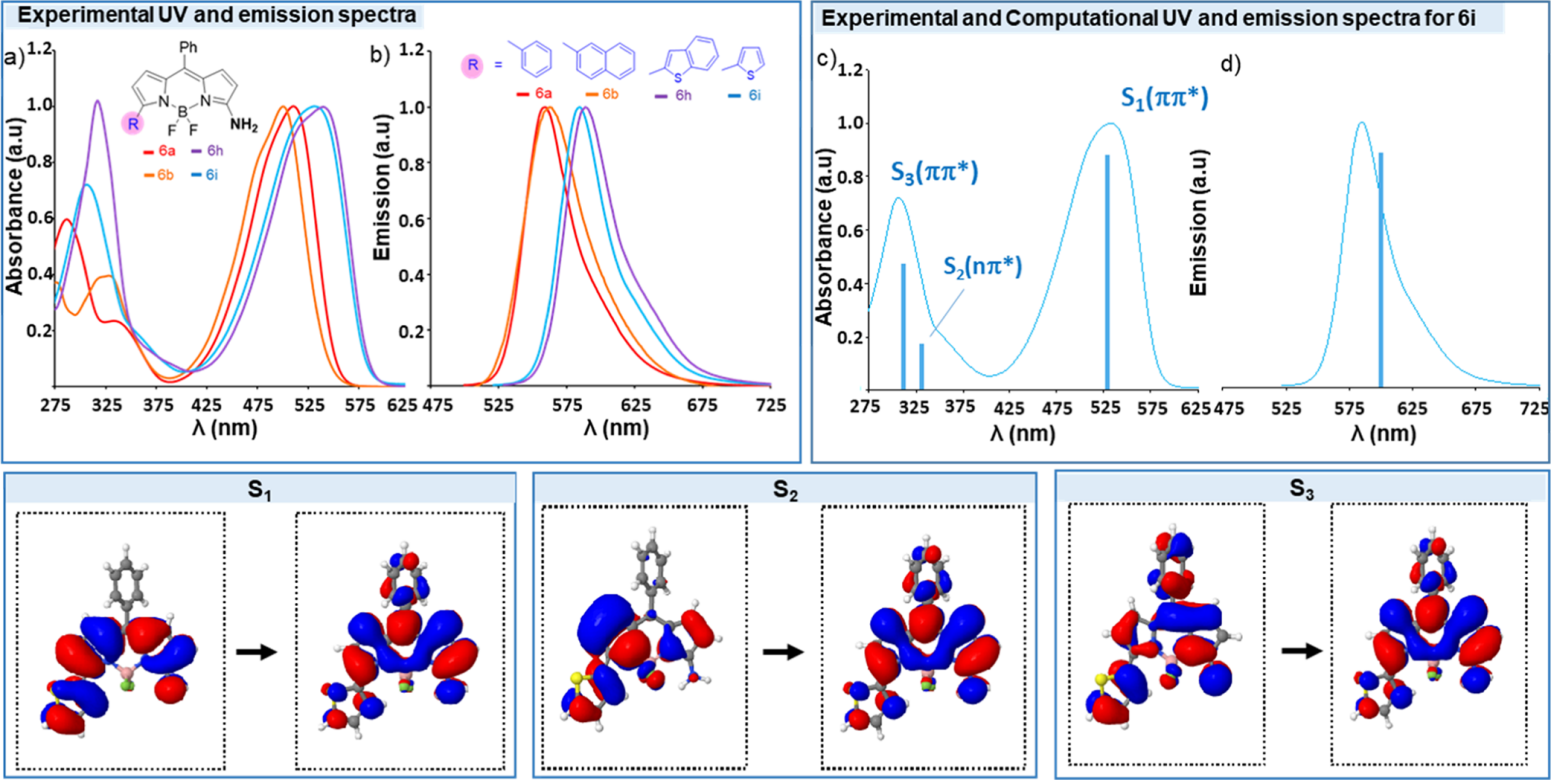
(Top left) Experimental (a) absorption and (b) emission spectra of representative derivatives in DCM exciting (λ_ex_) at 511 nm (**6a**), 500 nm (**6b**), 531 nm (**6h**), and 525 nm (**6i**). (Top Right) RI-ADC(2)/def2-SVP vertical energies superimposed to the experimental (c) absorption and (d) emission spectra of **6i** (red-shift applied 0.14 eV). (Bottom) Natural transition orbitals for the lowest-lying excited electronic states S_1_–S_3_ involved in the main absorption and emission bands.

On the contrary, the 3-amino-BODIPY derivatives **6** exhibit an intense emission fluorescence in the green-red range λ_em_ 552–589 nm (see Figure 3b for representative examples, Table S3 and Figure 3d for theoretical calculations of **6i**).

The UV–vis spectra of **6** show a maximum absorption at 493–531 nm (Figure 3a), calculated for **6i** at λ 500 nm and assigned to S_1_(ππ*) (Figure 3c). This band is characteristic of the BODIPY and is mainly localized over the pyrrole and thienyl rings (Figure 3 bottom S_1_). The substitution pattern at C-5 alters the position of both the absorption (up to 60 nm shift) and emission (up to 37 nm shift) maxima. Compounds **6h** (λ_em_ 589) and **6i** (λ_em_ 585) have the most red-shifted emission. For the biological studies, the UV–vis and emission spectra of **6i** were also measured in a PBS/DMSO solution, showing values of λ_abs_ = 500 nm and λ_em_ 585 nm. The fluorescence quantum yields (Φ) of **6** varied from 0.24 to 0.95 (Table S3). The highest values were obtained for the thienyl derivatives **6h** (Φ 0.91) and **6i** (Φ 0.95 in DCM and 0.4 in DMSO/PBS). The Stokes shifts are significantly large (from 50 to 85 nm, Table S3) for a BODIPY derivative, which is very interesting to allow for high-sensitivity bioimaging.^21^ All these results confirmed that the azo group situated at C-3 position of the BODIPY acts as a strong fluorescence quencher and that the 3-amino-BODIPY **6**, obtained upon the reductive azo cleavage, successfully recovers the BODIPY emission.

**Figure 4:**
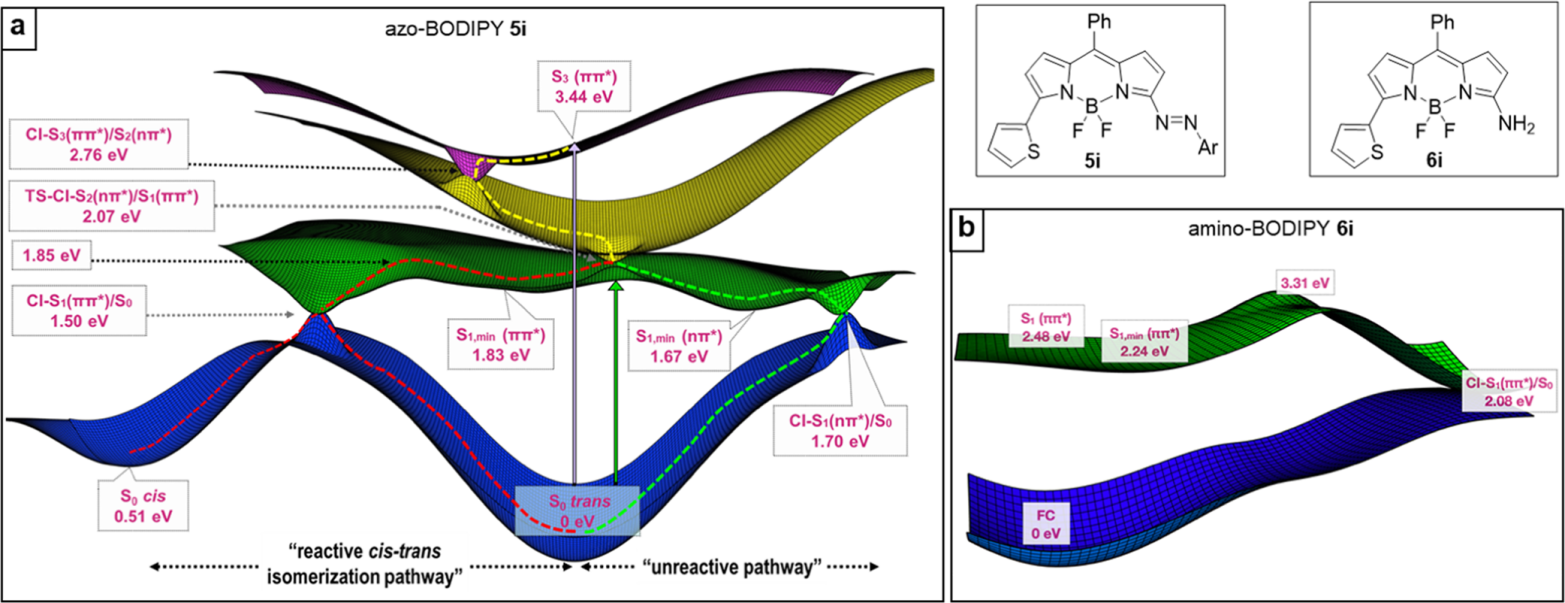
Potential energy landscape of (a) azo-BODIPY **5i** and (b) amino-BODIPY **6i** obtained at the RI-ADC(2)/def2-SVP level of theory.

The photophysical properties of the 3-azo-BODIPY and the 3-amino-BODIPY derivatives can be readily explained from the topography of the excited potential energy surfaces along the coordinates relevant to the deactivation of these systems (Figure 4). Interestingly, the coupling of the azo moiety to the BODIPY dramatically modifies the landscape of the excited state potential energy surface of the fluorophore. In fact, the deep S_1_ minimum characteristic of the parent BODIPY compound and its meso substituted derivatives^22a−22c^ (Figure 4b), responsible for the remarkable fluorescence of these species, is replaced in the azo-BODIPY system (Figure 4a), by two very shallow minima, S_1,min_(ππ*) and S_1,min_(nπ*), in the same potential (accessible after irradiation at 630–700 nm). The first minimum, located in the vicinity of the Franck–Condon region (FC), and thus very similar to the trans ground state isomer, presents ππ* character, while the second of nπ* nature would present overstretched CNN angles (see stationary geometries in Figure S9). A quite small barrier separates both minima, ensuing the exchange of population between them. For its part, a rather planar potential connects these minima with two internal conversion funnels, CI-S_1_(ππ*)/S_0_ and CI-S_1_(nπ*)/S_0_, as shown in Figure 4, (CI = conical intersection). CI-S_1_(ππ*)/S_0_ defining the “reactive pathway” would bifurcate the population between the cis-S_0_ and trans-S_0_ minima (see Figure 4a, red line path), while CI-S_1_(nπ*)/S_0_ would exclusively return the population to the original S_0_ trans minimum by the pathway name “unreactive” (see Figure 4a, green line path). The accessibility of the CI-S_1_(ππ*)/S_0_ and CI-S_1_(nπ*)/S_0_ internal conversion funnels from the S_1_ minima is consistent with the absence of fluorescence experimentally observed for these systems.

Our minimum energy path calculations also predict a downhill potential energy profile from S_3_ (state predominantly accessed after 370–380 nm irradiation) to S_1_. Specifically, decay from S_3_ would direct the population first toward the CI-S_3_(ππ*)/S_2_(nπ*) crossing and then to the CI-S_2_(nπ*)/S_1_(ππ*) crossing (Figure 4a, yellow line path), the latter located near the S_1_ transition state connecting the ππ*and nπ* minima, TS (ππ*–nπ*). This crossing is expected to distribute population between the two S_1,min_(ππ*) and S_1,min_(nπ*) minima. The exclusive experimental identification of the *trans*-azo-BODIPY isomer, following irradiation of the azo-BODIPY **5i** either with red, green, or UV photons, discards the potential deactivation of the system through the expected ultrafast trans/cis isomerization CI-S_1_(ππ*)/S_0_ funnel (Figure 4a, red line path) and points to S_3_FC → CI-S_3_/S_2_ → CI-S_2_/S_1_ → [S_1,min_(ππ*)] → S_1,min_(nπ*) → CI-S_1_(nπ*)/S_0_ → trans-S_0_ as the most probable decay mechanism for this system (Figure 4a, green line path).

However, in view of the similar energetics accessibility of both deactivation paths, we infer that the preference for the unreactive mechanism can be only attributed to dynamical effects, which can only be accounted for when undertaking molecular dynamics simulations. The preference, although no exclusiveness, for the nonreactive pathway has been actually observed in previous works on the simpler azobenzene system, which presents a very similar flat S_1_ potential energy surface along the nπ* and ππ* coordinates.^22d^ The restoration of the characteristic fluorescent properties of BODIPY upon the reduction of the azo group is also imprinted in the potential energy surfaces of the reduced species. As can be seen in Figure 4b, the population of the 3-amino-BODIPY **6i** excited to S_1_ would be directed toward a planar S_1,min_, vertically located at 574 nm (observed λ_em_ 552–589 nm). Moreover, in this case, the nonradiative decay of the population is hindered by an activation barrier that intercalates the minimum from the CI-S_1_(ππ*)/S_0_ conical intersection. Interestingly, while CI-S_1_/S_0_ for the amino-BODIPY shares some structural similarity to the most stable CI computed for the parent BODIPY,^22b,22c^ that is, butterfly folding along the C8–B axis and the tilting of the group/H located at the meso position, C3-functionalization of the BODIPY core alters the energetics of the decay paths of the chromophore. In particular, the incorporation of an amino group at C3 shifts CI-S_1_/S_0_ below the energy of the minimum, while the inaccessibility of the S_1_/S_0_ funnel and thus the fluorescent properties are preserved due to the existence of a barrier between the minimum and CI.

Next, we studied the potential use of these azo-BODIPY as turn-on fluorescent biosensors of reductive media. To this aim, the enzymatic reduction of azo-BODIPY **5** was studied using two different azoreductases capable of cleaving azo bonds, using nicotinamide adenine dinucleotide (NADH) or flavin-adenine-dinucleotide (FAD) as cofactors. Specifically, we tested the bacterial *Bacillus cereus* azoreductase (*azoRBC*) and a human NADH-quinone oxido-reductase (NQO1). The enzymatic reduction was initially tested with azo-BODIPY **5i**, and the azoreductase activity was measured as a function of the decrease in the intensity of the 615 nm absorption band (absorption maxima using H_2_O/EtOH 1:1). As a reference, the enzymes activities were evaluated with commercially available Methyl Red azocompound (λ_abs_ 430 nm), that is known to be cleaved by these enzymes. Bacterial *azoRBC* is a homodimeric enzyme of low molecular weight (21.5 kDa for each subunit), with optimum working conditions at pH 6–7 at 40 °C. Due to the low solubility of **5i** in aqueous enzymatic medium, the photophysical properties were measured in a 1:1 H_2_O/EtOH mixture, which allowed for increasing the substrate concentration 10 times (**5i** from ≈2.5 to 25 μM) and the reduced product concentration more than 10 times. However, in this solvent mixture, the bacterial *azoRBC* formed aggregates, inactivating and preventing the total reduction of the substrates (see Table S4). To avoid such inactivation, the enzyme was immobilized on a solid support (agarose gel) coated with polyethyleneimine (PEI). *azoRBC* was immobilized (*i-azoRBC*) by an adsorption method promoted by the ionic exchange between several carboxyl groups of each enzyme molecule and several ionized amino groups of a PEI-coated agarose support (Figure S13). Thus, the highly hydrophilic environment surrounding each enzyme molecule can also protect it from other negative effects promoted by the presence of EtOH that is necessary to solubilize substrates and products. The immobilization was complete in less than 1 h, retaining 100% of the activity, and *i-azoRBC* contains 0.33 mg of enzyme per gram of the catalyst (see Figure S14). The stability study comparing the free and the immobilized enzyme incubated in H_2_O/EtOH (1:1) showed that *i-azoRBC* was much more stable and retained more than 90% of its activity after 50 h, whereas the free enzyme was rapidly inactivated, and so, it would not be feasible to do long reduction tests (see Figure S15).

**Figure 5:**
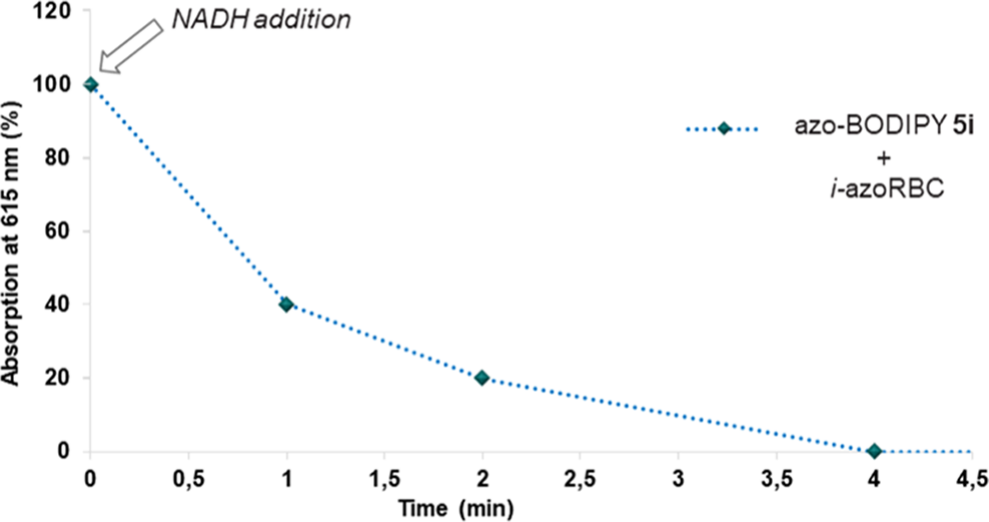
Time-course of the enzymatic reduction of azo-BODIPY **5i** with *i-azoRBC* and NADH (1:1 EtOH/TRIS buffer at pH 7.0). The 100% absorbance at 615 nm corresponds to 0.3 a.u.

The reduction process was carried out in the presence of the reducing cofactor, NADH, and monitored by the decrease of absorbance at 615 nm for **5i**. Without NADH, the absorbance remains unaltered, and after its addition, it decreases to 0.001 in 4 min (see Figures 5 and S16). In the absence of the enzyme, the addition of NADH to **5i** does not modify the absorbance at 615 nm. These results indicate that the release of the fluorophore must occur by an enzyme reductive cleavage pathway.

The activity of the immobilized enzyme against a selection of azo-BODIPY derivatives and the activity percentage (considering 100% the activity with Methyl Red) are depicted in Table 1. The enzyme *i-azoRBC* showed very good results for **5c** (125%, Table 1, entry 4) and **5f** (110%, entry 6), good for other derivatives such as 5-chloro-arylazo BODIPY **3** (80%, entry 3) and moderate for the *ortho*-methoxy derivative **5e** (30%, entry 5).

**Table 1:**
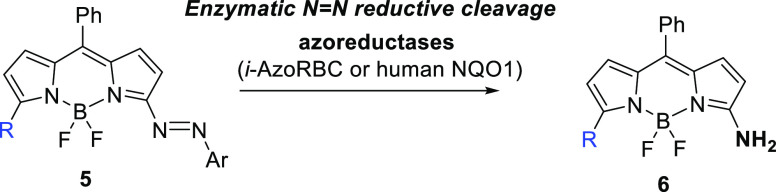
Enzymatic Reductive Cleavage of N=N of Azocompounds

			activity^a^ (%)^b^	
entry	azo	λ_abs_ (nm)	*i-azoRBC*	NQO1
1	methyl red	430	4 (100)	2 (100)
2	**5i**	615	4 (100)	0.6 (30)
3	**3**	608	3.2 (80)	1 (50)
4	**5c**	610	5 (125)	3.4 (170)
5	**5e**	614	1.2 (30)	1 (50)
6	**5f**	616	4.4 (110)	0.5 (25)
7	**5g**	630	2 (51)	1.4 (70)

a Activity = μmoles/min of reduced azocompound per mg of the enzyme used.

b Activity percentage considering 100% the activity with methyl red.

The reductive cleavage of azo-BODIPY was also tested with less accessible human enzyme NADH-quinone oxido-reductase (NQO1), and the activity was compared with the immobilized *i-azoRBC* (Table 1). NQO1 is a dimeric redox enzyme (with 30,000 kDa per subunit), dependent on the FAD cofactor and present in a large number of solid tumors in all types of organisms.^23^ NQO1 contains two active centers that are capable of giving up four electrons to reduce and break azo bonds and convert them into two amines. The reduction with NQO1 should be carried out in a completely aqueous media, and therefore, the concentration of azo-BODIPY **5** was required to decrease from 25 to 2.5 μM. Methyl red was also taken as a reference and considered as the 100% of the activity percentage (Table 1, NQO1 column, entry 1).

Finally, we evaluated the potential use of the azo-BODIPY **5i** as turn-on fluorescent biosensors in living cells. The initial toxicity studies were evaluated using the MTT assay,^24^ incubating HeLa cells for 2 h with both compounds, azo-BODIPY **5i** and the reduction product **6i**, at different concentrations (2, 5, 10, and 20 μM in complete medium, using DMSO as a cosolvent). The survival rates after 24 h showed no significant toxicity at concentrations up to 20 μM for both derivatives (Figure S17).

**Figure 6:**
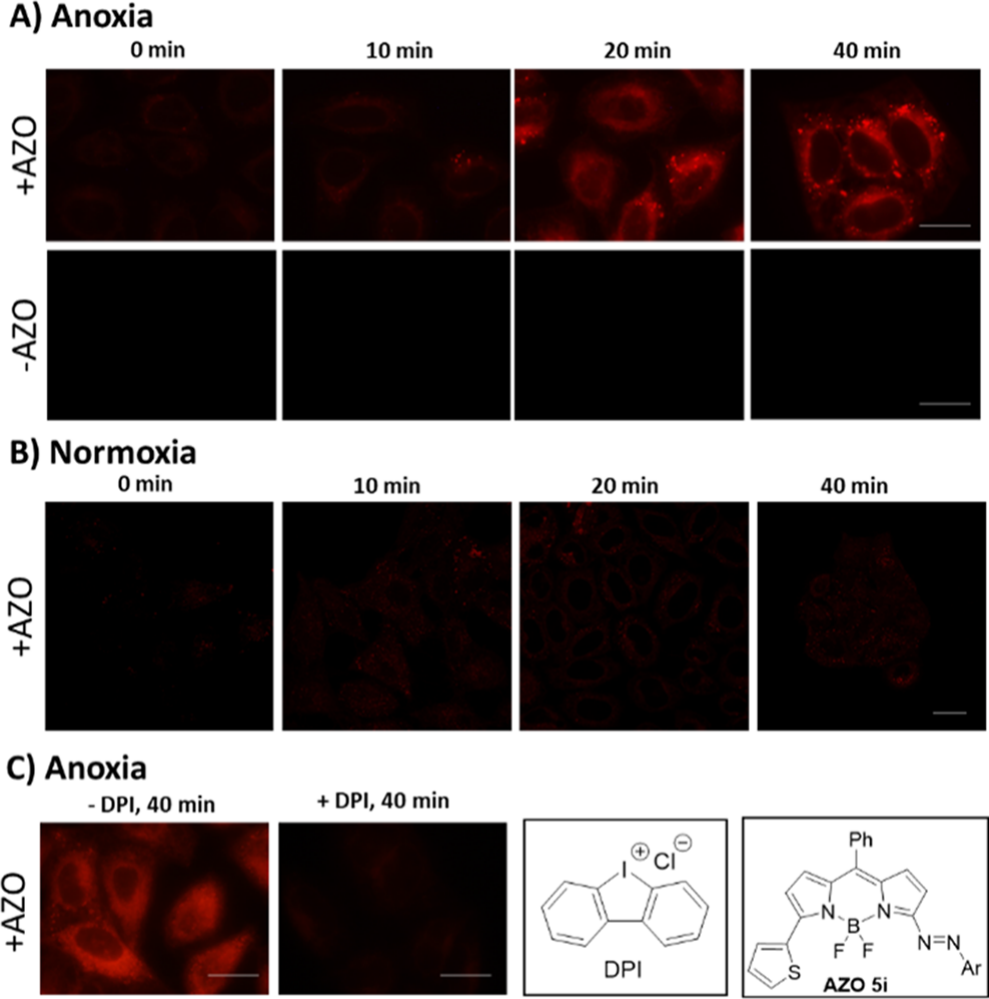
(A) Fluorescence images of HeLa cells incubated for 2 h with **5i** (+AZO) 10 μM and without **5i** (−AZO) and subjected to different oxygen deprivation times (10, 20, and 40 min). (B) Confocal images of HeLa cells incubated with **5i** for 2 h under normoxia at different times. (C) Fluorescence images of HeLa cells incubated with **5i** without DPI (−DPI) and with 10 μM DPI (+DPI) for 2 h and subjected to 40 min of oxygen deprivation time. The fluorescent images were obtained under green light excitation. The confocal images were obtained using excitation at 561 nm. Scale bar: 20 μM.

Live cell imaging studies were performed using **5i** in HeLa cells gradually subjected to an oxygen free atmosphere.^25^ It has been reported that the thin cover glass placed over a cell culture plate blocks the oxygen supply to the cells beneath it, resulting in a decrease of oxygen concentration and generating anoxia or different levels of hypoxia.^10a,10b,26^ Under such conditions, the azo-based fluorescent quenchers release the fluorophore upon cleavage of the N=N double bond by the inside cell azoreductases. In our case, HeLa cells were dosed with azo-BODIPY **5i** (10 μM) and incubated for 2 h.^27^ Then, the cells grown on coverslips, were placed on slides, and analyzed under a fluorescence microscope at different times (0, 10, 20, and 40 min). The fluorescence imaging studies revealed a red intracellular fluorescence increase under this oxygen-deprived condition (Figure 6A, +AZO). HeLa cells under the same oxygen-deprived condition but without **5i** showed no fluorescent signal (Figure 6A, −AZO). We tested the selectivity of **5i** under normoxic conditions (without the cover glass on top) and no fluorescence increase occurred (Figure 6B). (For the quantification of fluorescence intensity, see Figure S18).

Moreover, the inhibition of the azoreductase activity, using DPI (10 μM, diphenyleneiodonium chloride),^28,29^ under anoxic conditions (40 min) revealed no fluorescence increase (Figure 6C). All these results indicate that azo **5i** is reduced by reductases and help selectively differentiate between normoxic and anoxic conditions inside the HeLa cells.

In conclusion, a family of 3-arylazo-BODIPY dyes as novel fluorescent quenchers has been prepared. Our design allowed for an easy conjugation of the BODIPY fluorophore directly to the azo moiety under very mild conditions, complementary to the usual azo-coupling reaction that requires the preparation of diazonium salts. Our approach also allowed for the final tuning of the electronic properties of the BODIPY by the Suzuki cross-coupling reaction of different aryl and heteroaryl groups at C-5. The azo moiety is able to extinguish the emission of the BODIPY by a nonradiative process operating under a non-FRET mechanism. Our calculations predict that the coupling of azobenzene to the BODIPY triggers a restructuration of the topography of the S_1_ potential energy surface by substituting the deep minimum responsible for the fluorescence in the parent BODIPY compound for two shallow minima in the azo-BODIPY. From these minima, of ππ* or nπ* character, the access of two internal conversion funnels is thermodynamically feasible, allowing for the return of the population to the ground state. The absence of the cis-isomer during the experimental photoisomerization studies at different wavelengths discards the usual ultrafast trans/cis deactivation pathway and suggested that only the unreactive nπ* pathway is operative. The reduction of this azo-BODIPY allows us to access to 3-amino-BODIPY derivatives that possess a strong absorption in the green-red region, with large molar absorption coefficients and relatively large stokes shifts. The amino-BODIPY species are governed by an excited state potential energy profile, where nonnegligible energy barriers preclude the access of the internal conversion funnels to the ground state, which translate into fluorescence emission. Moreover, an immobilized bacterial azoreductase (*i-azoRBC*) protocol has been probed as a reliable model to study the azo-reducing capacity of less accessible human reductase NQO1, present in solid tumors. These results are very promising for future biological applications, as it would allow the most suitable azo compound to be selected following a simple enzymatic activity assay. The azo bond was reductively cleaved under enzymatic conditions and also in HeLa cells lacking oxygen, proving its potential application as turn-on fluorescent hypoxia probes.

## Supporting Information Available

Experimental procedures, UV–vis and emission spectra, fluorescence quantum yields, additional computational and enzymatic details, fluorescence microscopy images, fluorescence intensity quantification, ^1^H and ^13^C NMR spectra of all compounds (PDF)
