# Supplementary material for: Turn-on Fluorescent Biosensors for Imaging Hypoxia-like Conditions in Living Cells
Source: J Am Chem Soc. 2022 Apr 29;144(18):8185–93. doi: 10.1021/jacs.2c01197 (PMC9100661; doi:10.1021/jacs.2c01197)
Supplement: Supplementary file 1 — ja2c01197_si_001.pdf [file ja2c01197_si_001.pdf]

SUPPORTING INFORMATION

Turn on Fluorescent Biosensors for Imaging Hypoxia-like Conditions in Living Cells

Santiago Guisan-Ceinos,[a,‡] Alexandra R. Rivero,[a,‡] Fernando Romeo-Gella,[b] Silvia Simón-Fuente,[a] Silvia Gómez-Pastor,[c] Natalia Calvo,[c] Alejandro H. Orrego,[d] José Manuel Guisán,[d,*] Inés Corral,[b,e] * Francisco Sanz-Rodriguez,[c,*] Maria Ribagorda[a,e]*

[a] Departamento de Química Orgánica, Facultad de Ciencias, Universidad Autónoma de Madrid, C/ Francisco Tomás y Valiente 7, 28049-Madrid, Spain.
[b] Departamento de Química, Facultad de Ciencias, Universidad Autónoma de Madrid, 28049-Madrid, Spain.
[c] Departamento de Biología, Facultad de Ciencias, Universidad Autónoma de Madrid, 28049-Madrid, Spain.
[d] Departamento de Biocatálisis, Instituto de Catálisis y Petroquímica (CSIC), Campus UAM, 28049-Madrid. Spain.
[e] Institute for Advanced Research in Chemical Sciences (IAdChem), Universidad Autónoma de Madrid, Madrid-28049, Spain.

*Corresponding authors

maria.ribagorda@uam.es

ines.corral@uam.es

francisco.sanz@uam.es

jmguisan@icp.csic.es

Table of contents

1. EXPERIMENTAL PROCEDURES 2

2. SYNTHESIS OF STARTING MATERIALS 2

3. One pot synthesis of 5-chloro-3-arylazo-BODIPY 3 4

4. PREPARATION AND CHARACTERIZARION OF AZO BODIPY 5a-i 4

4.1 Suzuki cross-coupling optimization 4

4.2 General method A: Synthesis of 5-Aryl (heteroaryl)-3-arylazo BODIPY 5a-i 5

5. PREPARATION AND CHARACTERIZARION OF 3-AMINO-BODIPY 6a-i 10

5.1 General method B: Reductive cleavage of N=N azo bond 10

6. UV-Vis/EMISSION SPECTRA 15

6.1 Fluorescence Quantum Yields 17

7. COMPUTATIONAL STUDIES 18

7.1 Computational details 18

7.2 Reactive ground and excited PES for azo-BODIPY 5i 19

7.3 Calculated absorption spectra and NTOs (Natural Transition Orbitals) 21

7.4 Stationary geometries 5i 24

7.5 Stationary geometries 6i 25

8. ENZYMATIC AZO BOND CLEAVAGE STUDIES 26

8.1 BACTERIAL AZOREDUCTASE (*azoRBC*) 26

a) Expression, production and purification of *azoRBC* from the microorganism bacillus cereus 26

8.2 Preparation of *i-azoRBC*: Enzymatic immobilization on agarose PEI 27
8.3 Enzymatic Activity and Stability studies 28
a. ACTIVITY OF *i-azoRBC* 28
b. ENZYMATIC STABILITY STUDIES of *i-azoRBC* 29
8.4 ENZYME ASSAYS 29
8.5 TIME-COURSE OF THE ENZYMATIC REDUCTION OF AZO-BODIPY 5i 31
9. CELL CULTURE STUDIES 32
9.2 Administration of compounds 32
9.3 Intracellular localization. 32
9.4 MTT viability assay 32
9.5 Fluorescence Microscopy 33
10. NMR SPECTRA OF NEW COMPOUNDS 35
REFERENCES 54

1. EXPERIMENTAL PROCEDURES

Unless otherwise indicated, chemicals were purchased from Sigma-Aldrich, Fluorochem and Alfa Aesar Chemicals and were used without further purification. Air sensitive reactions were performed using oven-dried glassware (Oven = 150 °C) and performed under a nitrogen atmosphere using Schlenk techniques. Solvents were dried on a solvent purification system (PS-MD-5/7 Inert technology). Reactions were monitored by thin-layer chromatography (TLC) on silica-gel-coated aluminum foils (silica gel 60 F254, Merck). The TLC plates were visualized by UV light ($\lambda = 254$ nm). Flash-column chromatography was performed on silica gel 60. NMR spectra were recorded on a Bruker AV-300 spectrometer at 25 °C. Chemical shifts (δ) are reported in ppm relative to the residual solvent peak. Splitting patterns are indicated as (s) singlet, (d) doublet, (t) triplet, (q) quartet, (m) multiplet and (br) broad. Coupling constants (J) are reported in Hertz (Hz). High-resolution mass spectra (HRMS) were recorded on a Waters XEVO-G2 QTOF mass spectrometer, using electron impact (EI) or electrospray ionization (ESI) in positive or negative mode, depending on the analyte. UV-Visible experiments were conducted using a JASCO V-660 apparatus at room temperature. Emission spectra were recorded in a JASCO FP-8600 equipment. Quartz cuvettes were used for the measurements, in particular Hellma® precision cells made of Quartz Suprasil ® (10 x 10 mm). In these two instruments the temperature was controlled using a JASCO Peltier thermostated cell holder with a range of 263–383 K, adjustable temperature slope, and accuracy of ± 0.1 K. Solvents were purified by use of drying cartridges through a solvent delivery system. All chemicals were used as received unless otherwise noted. BODIPY **1**[1] and **4**[2] were synthesized according to the literature. Bis-dimethyl acetal of *p*-benzoquinone **2** is commercially available and can also be synthesized according to the literature.[3]

2. SYNTHESIS OF STARTING MATERIALS.

2.1. Synthesis of 2,5-Dichloro-8-phenyl-BODIPY[2b]

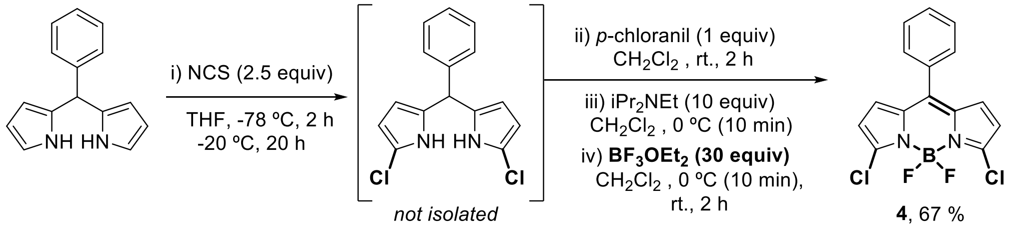

To a two necked 250 mL round bottom flask equipped with a stir bar was added 5-phenyl-dipyrromethane[4] (1.0 g, 4.5 mmol, 1.0 equiv.), the flask was evacuated and flushed with argon three times before 150 mL of dry tetrahydrofuran (0.03M) was added. The solution was cooled to -78 °C before N-chlorosuccinimide (2.5 equiv.) was added under an argon flow in one portion. The reaction mixture was stirred at -78 °C for 2 h and at -20 °C overnight. The reaction mixture was diluted with water and extracted with DCM. The combined organic solvent was washed with brine, dried over $MgSO_4$ and concentrated under reduce pressure. The obtained residue was placed in a 250 mL round-bottom flask and *p*-chloranil (1.1 g, 4.5 mmol, 1.0 equiv.) was added. Then, under an argon atmosphere dry dichloromethane (0.03 M) was added and the resulting reaction mixture stirred at rt for 2 h. Then, DIPEA (5.8 g, 7.9 mL, 45 mmol, 10 equiv.) was added at 0 °C, after 10 min BF_3·OEt_2 (19.2 g, 16.6 mL, 135 mmol, 30 equiv.) was added also at 0 °C. The reaction was allowed to warm to rt and stirred for 2 h. Then, saturated $NaHCO_3$ (30 mL) was slowly added to the crude mixture at 0 °C and extracted with DCM (30 ml x 3). The combined organic layers were dried over $MgSO_4$, the solvent was concentrated under reduce pressure and residue was purified by flash column chromatography using heptane: CH_2Cl_2 (1.5:1) as eluent to afford **4** as bright red powder (1 g, 67% yield).**^{1}H NMR** (300 MHz, $CDCl_3$): δ = 7.70-7.43 (m, H), 6.95 (d, *J* = 4.3 Hz, 2H), 6.81 (d, *J* = 4.3 Hz, 2H).

2.2. Synthesis of bis-dimethyl acetal of *p*-benzoquinone (3,3,6,6-tetramethoxycyclohexa-1,4-diene) 2[3]

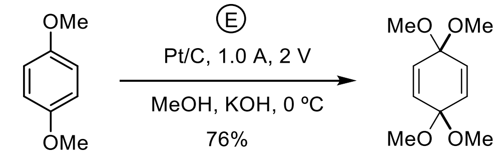

A solution of 1,4-dimethoxybenzene (17 g, 123 mmol) and KOH in 200 mL of MeOH was anodically oxidized at -5 °C, under constant current (1.0 A, 2 V), [Electrolysis was carried out in a single cell apparatus, using a circular platinum gauze anode (5 cm x 5 cm in diameter) 45-mesh, a graphite cathode (9 mm x 40 mm), and an AMEL (model 549) power supply]. The reaction was monitored by TLC. When the reaction was completed, the methanolic solution was evaporated *in vacuo* to afford a pale orange paste. This material was dissolved in ca. 200 ml of hot petroleum ether, filtered, and washed with water and saturated brine solution. The combined organic phases were dried over anhydrous $MgSO_4$, filtered and concentrated *in vacuo*, affording a light yellow solid which was recrystallized from petroleum ether to yield **2** as white crystals in 76% yield (18.6 g, 98.89 mmol).**^{1}H NMR** (300 MHz, $CDCl_3$): δ = 6.10 (s, 4H), 3.30 (s, 12H).

3. One pot synthesis of 5-chloro-3-arylazo-BODIPY 3

3-Chloro-5-(4-methoxyphenyl)diazenyl-8-phenyl-4,4-difluoro-4-bora-3a,4a-diaza-s-indacene 3

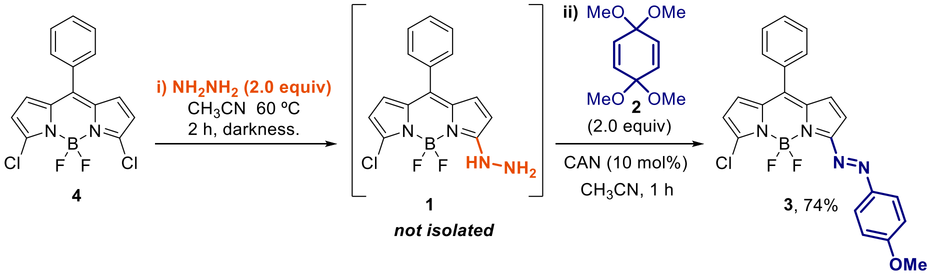

3,5-Dichloro BODIPY **4** (1.0 g, 3.0 mmol) was added to an oven dried microwave tube; the tube was sealed, evacuated and flushed with argon three times before dry acetonitrile (0.03 M) and $N_2H_4 \cdot H_2O$ (2.0 equiv) were added. Then, the resulting solution was heated at 80 ºC for 2 h. After that period of time the reaction mixture was cooled to room temperature and the tube was opened before 1,1,4,4-tetramethoxycyclohexa-1,4-diene **2** (2.0 equiv) and CAN (10 mol %) were added (after the addition of CAN, a fast and drastic change of color from dark red to deep dark blue was observed). Finally, when the reaction was finished (monitored by TLC), the solvent was evaporated under reduce pressure and the residue was purified by flash column chromatography using Heptane: CH_2Cl_2 (1:1) as eluent to afford the desire product as a dark powder in 73% yield (967 mg, 2.2 mmol). **^{1}H NMR** (300 MHz, $CDCl_3$): δ = 8.09 (d, *J* = 9.1 Hz, 2H), 7.72 – 7.43 (m, 5H), 7.02 (d, *J* = 9.1 Hz, 2H), 6.91 (s, 2H), 6.88 (d, *J* = 4.4 Hz, 1H), 6.47 (d, *J* = 4.4 Hz, 1H), 3.91 (s, 3H). **^{13}C NMR** (75 MHz, $CDCl_3$): δ = 164.0, 162.5, 148.7, 144.8, 143.8, 135.9, 134.4, 131.3, 131.1, 130.6, 130.5, 128.5, 126.6, 118.9, 114.6, 108.3, 55.7. **^{19}F NMR** (282 MHz, $CDCl_3$): δ = -143.45 (q, *J* = 27.0 Hz).**^{11}B NMR** (96 MHz, Acetone): δ = 0.93 (t, *J* = 26.6 Hz). **HRMS (ESI):** Calcd for $C_{22}H_{16}BClF_2N_4O$ (M+): 436.1074 found: 436.1079.

4. PREPARATION AND CHARACTERIZARION OF AZO BODIPY 5a-i

4.1 Suzuki cross-coupling optimization

Table S1: Optimization of the Suzuki reaction between **4** and phenyl boronic acid

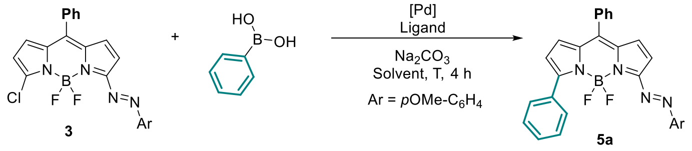

Entry	Catalyst	mol% Catalyst/Ligand	Solvent	T (ºC)	M	5a Yield (%)^a
1	$Pd(PPh_3)_4$	10	Toluene	110	0.1	7
2	$Pd(PPh_3)_4$	10	Dioxane	110	0.1	6
3	$Pd(PPh_3)_4$	10	DME	65	0.1	14
4	$Pd(acac)_2$	10	DME	65	0.1	-
5	$Pd(dppe)Cl_2$	10	DME	65	0.1	-
6	$Pd(dppe)Cl_2$	10	DME	65	0.1	-
7	$Pd(dppf)Cl_2$	10	DME	65	0.1	26
8	XPhosPdG2	10	DME	65	0.1	12
9	$Pd(CH_3CN)_2Cl_2$	10	DME	65	0.1	12
10	$Pd(dba)_3$	10	DME	65	0.1	21
11	$Pd(OAc)_2/P(^tBu)_3$	10/10	DME	65	0.1	-
12	$Pd(OAc)_2$/P(2-furyl)$_3$	10/10	DME	65	0.1	-
13	$Pd(OAc)_2$/P(2-OMePh)$_3$	10/10	DME	65	0.1	54
14	$Pd(OAc)_2$/P(2-OMePh)$_3$	10/20	DME	65	0.1	61
**15**	**$Pd(OAc)_2$/P(2-OMePh)$_3$**	**5/10**	**DME**	**65**	**0.05**	**80**

Reaction conditions: On a sealed tube under argon, **4**: phenyl boronic acid: base 1:1.5:3, catalyst, ligand and solvent were heated for 4 h. ^{a} Isolated yields.

4.2 General method A: Synthesis of 5-Aryl (heteroaryl)-3-arylazo BODIPY 5a-i

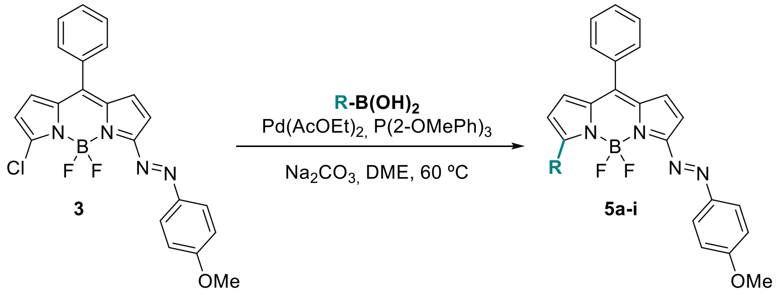

General procedure A: To an oven dried microwave tube was added 5-chloro-arylazo BODIPY **3** (100 mg, 0.23 mmol, 1.0 equiv), the corresponding boronic acid acid (2.0 equiv), $Pd(OAc)_2$ (5 mol%), P(2-OMe-Ph)$_3$ (10 mol%) and Na_2CO_3 (3.0 equiv). The tube was evacuated and flushed with argon three times before dry dimethoxyethane (0.05 M) was added. The resulting suspension was heated at 60 ºC for 2 h. Then, the reaction mixture was diluted with CH_2Cl_2 and filtered in short pad of celite. After that time the solvent was evaporated *in vacuo* and the residue was purified by flash column chromatography using a gradient of Heptane: CH_2Cl_2 from [2:1] to [1:1] as eluent to afford the desire product as a dark powder.

5-Phenyl-3-azo-BODIPY 5a

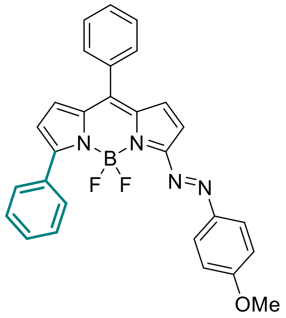

Following general method A, azo bodipy **5a** was isolated as a dark blue solid in 80% yield (88 mg, 0.18 mmol). **^{1}H NMR** (300 MHz, $CDCl_3$)**:** δ = 8.11-7.97 (m, 4H), 7.69 – 7.45 (m, 8H), 7.08-6.97, m, 3H), 6.88 (d, *J* = 4.9 Hz, 2H), 6.75 (d, *J* = 4.4 Hz, 1H), 3.90 (s, 3H). **^{13}C NMR** (75 MHz, $CDCl_3$): δ = 163.4, 148.6, 133.9, 132.3, 132.2, 132.1, 130.6 (2C), 130.2, 130.1, 129.7, 129.6, 128.4 (2C), 128.3 (2C), 126.2 (2C), 114.5 (2C), 55.7. **^{19}F NMR** (282 MHz, $CDCl_3$): δ = -135.27 (q, *J* = 29.1.0 Hz, 2F). **^{11}B NMR** (96 MHz, $CDCl_3$): δ = 1.47 (t, *J* = 29.1 Hz). **HRMS** (IE): Calculated for $C_{28}H_{21}BF_2N_4O$ (M+ H): 479.1854; found: 479.1855.

5-Naphtalenyl-3-azo-BODIPY 5b

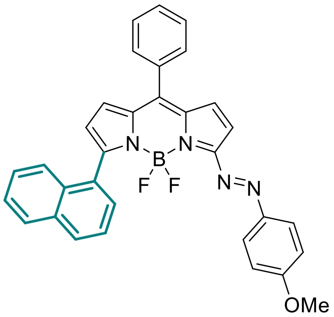

Following general method A, azo bodipy **5b** was isolated as a dark blue solid in 56% yield (67 mg, 0.13 mmol). **^{1}H- NMR** (300 MHz, $CDCl_3$): δ = 8.06 – 7.96 (m, 2H), 7.92 (d, *J* = 8.5 Hz, 3H), 7.87 (d, *J* = 8.3 Hz, 1H), 7.73 – 7.54 (m, 6H), 7.55 – 7.41 (m, 2H), 7.07 (d, *J* = 4.2 Hz, 1H), 6.96 – 6.84 (m, 4H), 6.70 (d, *J* = 4.4 Hz, 1H), 3.85 (s, 3H). **^{13}C- NMR** (75 MHz, $CDCl_3$): δ = 163.4, 161.7, 159.3, 148.5, 144.2, 138.4, 136.5, 135.9, 133.9, 133.5, 133.5, 132.8, 132.0, 130.9, 130.6 (2C), 130.4, 130.2, 130.0, 129.9, 128.8, 128.56, 128.4, 128.1, 127.9, 127.8, 126.5, 126.5, 126.3, 126.0, 126.0, 125.8, 125.3, 125.0, 123.5, 123.5, 114.4, 107.4 (2C), 55.6. **^{19}F- NMR** (471 MHz, $CDCl_3$): δ = -127.96 – -129.56 (m, 1F), -142.67 – -144.42 (m, 1F). **^{11}B- NMR** (96 MHz, $CDCl_3$): δ = 1.28 (t, *J* = 28.1 Hz). **HRMS** (EI): Calculated for Calcd for $C_{32}H_{23}BF_2N_4O$ (M+H): 529.2011; found: 529.2013.

5-(9-Anthracenyl)-3-azo-BODIPY 5c

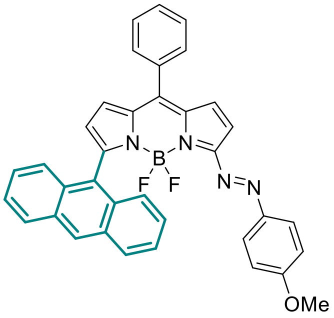

Following general method A, azo-BODIPY **5c** was isolated as a dark blue solid in 26% yield (34 mg, 0.06 mmol). **^{1}H-NMR** (300 MHz, $CDCl_3$): δ = 8.62 (s, 1H), 8.07 (d, *J* = 8.3 Hz, 2H), 7.87 – 7.69 (m, 6H), 7.67 – 7.56 (m, 3H), 7.53 – 7.32 (m, 4H), 7.20 (d, *J* = 4.2 Hz, 1H), 6.95 (d, *J* = 4.7 Hz, 1H), 6.88 – 6.78 (m, 3H), 6.66 (d, *J* = 4.2 Hz, 1H), 3.79 (s, 3H). **^{13}C- NMR** (75 MHz, $CDCl_3$): δ = 163.5, 148.4, 133.9, 131.1, 131.0, 130.7, 130.5, 128.7, 128.5, 128.3, 127.1, 126.8, 126.4, 125.8, 125.3, 114.3, 55.6. **^{19}F- NMR** (471 MHz, $CDCl_3$): δ = -139.86 (q, *J* = 27.8 Hz, 2F) **^{11}B- NMR** (96 MHz, $CDCl_3$): δ = 1.07 (t, *J* = 27.7 Hz). **HRMS** (ESI): Calculated for $C_{36}H_{25}BF_2N_4O$ (M+H): 579.2169; found: 579.2172.

5-(9-Phenanthracenyl)-3-azo-BODIPY 5d

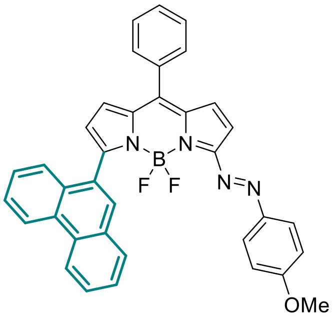

Following general method A, azo-BODIPY **5d** was isolated as a dark blue solid in 44% yield (58 mg, 0.10 mmol). ^{1}**H-NMR** (300 MHz, $CDCl_3$): δ = 8.77 (t, *J* = 7.9 Hz, 2H), 8.26 (s, 1H), 8.04 (d, *J* = 7.6 Hz, 1H), 7.89 (d, *J* = 8.5 Hz, 3H), 7.80 – 7.49 (m, 9H), 7.10 (d, *J* = 4.2 Hz, 1H), 6.93 (d, *J* = 4.4 Hz, 1H), 6.91 – 6.83 (m, 3H), 6.72 (d, *J* = 4.2 Hz, 1H), 3.81 (s, 3H). ^{13}**C- NMR** (75 MHz, $CDCl_3$): δ = 163.4, 148.6, 134.0, 130.9, 130.9, 130.9, 130.7, 130.4, 130.4, 130.1, 128.9, 128.5, 127.6, 127.0, 126.9, 126.8, 126.7, 126.3, 123.0, 122.6, 114.4, 55.6. ^{19}**F- NMR** (471 MHz, $CDCl_3$): δ = -127.84 – -130.63 (m, 1F), -142.33 – -144.65 (m, 1F). ^{11}**B- NMR** (96 MHz, $CDCl_3$): δ = 1.31 (t, *J* = 28.1 Hz). **HRMS** (ESI): Calculated for $C_{36}H_{26}BF_2N_4O$ (M+H): 579.2169; found: 579.2173.

5-(2-Methoxyphenyl)-3-azo-BODIPY (5e)

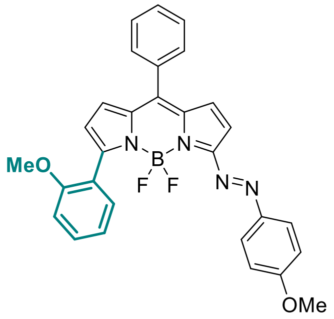

Following general method A, azo-BODIPY **5e** was isolated as a dark blue solid in 56% yield (65 mg, 0.13 mmol). ^{1}**H-NMR** (300 MHz, Acetone-d6): δ = 7.86 – 7.72 (m, 3H), 7.66 – 7.49 (m, 5H), 7.42 – 7.33 (m, 1H), 7.10 – 6.88 (m, 5H), 6.83 – 6.66 (m, 3H), 3.79 (s, 3H), 3.72 (s, 3H). ^{13}**C- NMR** (75 MHz, Acetone-d_6): δ = 164.5, 160.5, 159.0, 157.4, 154.5, 149.2, 144.6, 142.0, 137.8, 134.6, 132.6, 132.4, 131.6 (2C), 131.4, 129.6, 129.5 (2C), 126.5 (2C), 125.7, 122.5, 121.0, 115.6, 112.2 (2C), 107.0, 106.9, 56.2 (2C). ^{19}**F- NMR** (282 MHz, Acetone-d_6): δ = 42.21 (q, *J* = 28.3 Hz, 2F). ^{11}**B- NMR** (96 MHz, Acetone-d_6): δ = 1.25 (t, *J* = 28.3 Hz). **HRMS** (ESI): Calculated for $C_{29}H_{23}BF_2N_4O_2$ (M+H): 509.1882 found: 509.1884.

5-(2-Fluorophenyl)-3-azo-BODIPY (5f).

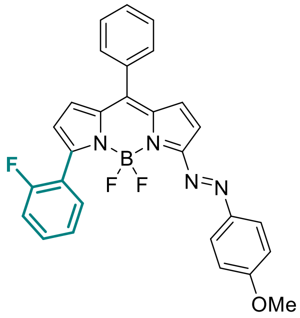

Following general method A, azo- BODIPY **5f** was isolated as a dark blue solid in 41% yield (47 mg, 0.09 mmol). **^{1}H-NMR** (300 MHz, $CDCl_3$): δ = 8.14 (td, *J* = 7.6, 1.8 Hz, 1H), 8.00 (d, *J* = 9.1 Hz, 2H), 7.71 – 7.42 (m, 6H), 7.34 (td, *J* = 7.6, 1.2 Hz, 1H), 7.19 (ddd, *J* = 9.7, 8.3, 1.2 Hz, 1H), 7.01 – 6.87 (m, 5H), 6.76 (t, *J* = 3.8 Hz, 1H), 3.89 (s, 3H). **^{13}C- NMR** (75 MHz, $CDCl_3$): δ = 163.9, 162.1, 148.6, 133.8, 131.6, 131.5, 130.6, 130.4, 130.3, 128.4, 126.4, 124.0, 123.0, 122.7, 115.8, 115.7, 115., 114.5, 107.7, 55.7. **^{19}F- NMR** (282 MHz, $CDCl_3$): δ = --112.48 (s, 1F), -111.90 (s, F), -136.08 (q, *J* = 28.6 Hz, 2F), -136.65 (q, *J* = 28.6 Hz, 2F. **^{11}B- NMR** (96 MHz, $CDCl_3$): δ = 1.29 (t, *J* = 28.7 Hz). **HRMS** (ESI): Calculated for $C_{28}H_{20}BF_3N_4O$ (M+H): 497.1760; found: 497.1762.

5-(3,4,5-Trimethoxyphenyl)-3-azo-BODIPY 5g.

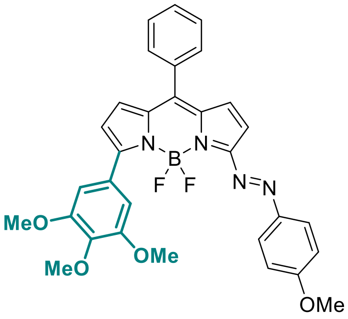

Following general method A, azo-BODIPY **5g** was isolated as a dark blue solid in 75% yield (98 mg, 0.17 mmol). **^{1}H-NMR** (300 MHz, $CDCl_3$): δ = 8.02 (d, *J* = 8.2 Hz, 2H), 7.71 – 7.48 (m, 5H), 7.37 (s, 2H), 7.10 – 6.94 (m, 3H), 6.89 (q, *J* = 4.8 Hz, 2H), 6.76 (d, *J* = 4.4 Hz, 1H), 3.97 – 3.91 (m, 12H). **^{13}C- NMR** (75 MHz, $CDCl_3$): δ = 163.4, 161.1, 160.9, 153.0, 148.7, 143.3, 143.1, 140.0, 137.9, 134.0, 132.2, 130.6, 130.3, 129.2, 128.4, 127.4, 126.1, 114.5, 107.4, 61.0, 56.3, 55.7. **^{19}F- NMR** (471 MHz, $CDCl_3$): δ = -134.87 (q, *J* = 29.3 Hz, 2F). **^{11}B- NMR** (96 MHz, $CDCl_3$): δ = 1.52 (t, *J* = 29.3 Hz). **HRMS** (ESI): Calculated for $C_{31}H_{27}BF_2N_4O_4$ (M+H): 569.2172; found: 569.2171.

5-(2-Benzo[b]thienyl)-3-azo-BODIPY 5h.

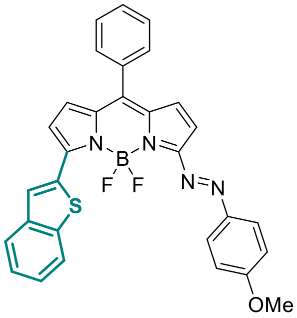

Following general method A, azo-BODIPY **5h** was isolated as a dark blue solid in 34% yield (42 mg, 0.08 mmol). **^{1}H-NMR** (300 MHz, $CDCl_3$): δ = 8.66 (s, 1H), 8.10 (d, *J* = 9.0 Hz, 2H), 8.05 – 7.94 (m, *J* = 9.4 Hz, 1H), 7.88 – 7.81 (m, *J* = 9.1 Hz, 1H), 7.65 – 7.49 (m, 5H), 7.46 – 7.36 (m, *J* = 9.2 Hz, 2H), 7.05 (d, *J* = 9.0 Hz, 2H), 6.97 (s, 2H), 6.91 (dd, *J* = 16.9, 4.5 Hz, 2H), 3.93 (s, 3H). **^{13}C- NMR** (75 MHz, $CDCl_3$): δ = 163.6, 148.8, 141.2, 140.9, 133.9, 133.9, 133.2, 133.2, 131.7, 131.7, 130.6, 130.3, 129.6, 128.5, 126.3, 126.2, 125.7, 124.9, 122.4, 121.9, 114.6, 107.9 (s), 55.7. **^{19}F- NMR** (471 MHz, $CDCl_3$): δ = -139.56 (q, *J* = 29.5 Hz, 2F). **^{11}B- NMR** (96 MHz, $CDCl_3$): δ = 1.67 (t, *J* = 29.5 Hz). **HRMS** (ESI): Calculated for $C_{30}H_{21}BF_2N_4OS$ (M+H): 535.1575; found: 535.1577.

5-(2-Thienyl)-3-azo-BODIPY 5i.

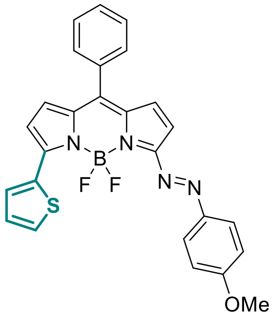

Following general method A, azo-BODIPY **5i** was isolated as a dark blue solid in 48% yield (53 mg, 0.11 mmol). **^{1}H-NMR** (300 MHz, $CDCl_3$): δ = 8.45 (d, *J* = 3.9 Hz, 1H), 8.07 (d, J = 8.4 Hz, 2H), 7.57 (q, *J* = 6.1, 5.6 Hz, 7H), 7.04 (d, *J* = 8.6 Hz, 2H), 7.00 – 6.85 (m, 3H), 6.82 (d, J = 4.7 Hz, 1H), 3.92 (s, 3H). **^{19}F-NMR** (471 MHz, $CDCl_3$): δ = -140.22 (q, *J* = 29.6 Hz, 2F). **^{11}B-NMR** (96 MHz, $CDCl_3$): δ = 1.61 (t, *J* = 29.5 Hz). **HRMS** (ESI): Calculated for $C_{26}H_{19}BF_2N_4OS$ (M+H): 485.1418; found: 485.1420.

5. PREPARATION AND CHARACTERIZARION OF 3-AMINO-BODIPY 6a-i

5.1 General method B: Reductive cleavage of N=N azo bond

Synthesis of 3-amino-5-aryl(heteroaryl)-BODIPY 5a-i

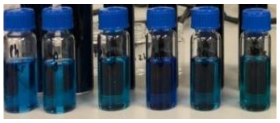

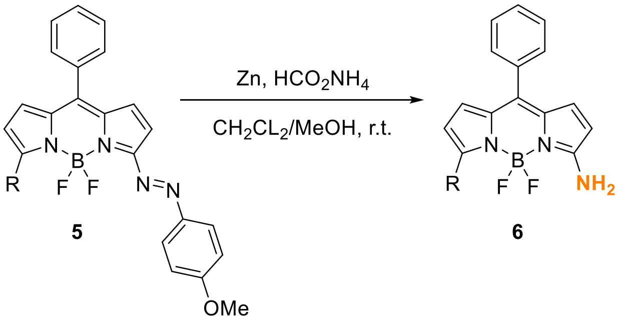

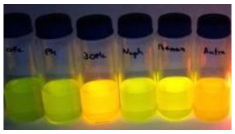

To a suspension of the corresponding azo-BODIPY **5** (1.0 equiv) and zinc dust (10 equiv) in CH_2Cl_2 (0,1 M), ammonium formate (20 equiv), dissolved in the minimum amount of methanol, was added and the mixture was stirred at room temperature. After the completion of the reaction (monitored by TLC) the reaction mixture was filtered through a pad of Celite, the solvent was evaporated *in vacuo* and the residue was purified by flash column chromatography using heptane:AcOEt as eluent to afford the desire product.

3-Amino-5-phenyl-BODIPY (6a).

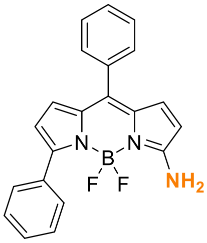

Following general method B, amino-BODIPY **6a** was isolated as an orange-red solid in 98% yield (70 mg, 0.19 mmol). ^{1}**H-NMR** (300 MHz, $CDCl_3$): δ = 7.83 (d, *J* = 6.97 Hz, 2H), 7.56 – 7.31 (m, 8H), 6.86 (d, *J* = 4.8 Hz, 1H), 6.50 (d, *J* = 3.8 Hz, 1H), 6.41 (d, *J* = 3.8 Hz, 1H), 6.03 (d, *J* = 4.8 Hz, 1H), 5.71 (s, 2H). ^{13}**C-NMR** (75 MHz, $CDCl_3$): δ = 160.7, 148.4, 134.9, 134.6, 134.4, 134.3, 132.6, 130.4, 129.2, 129.1, 129.1, 128.2, 127.9, 127.8, 122.3, 115.6, 112.5. ^{19}**F-NMR** (282 MHz, $CDCl_3$): δ = -142.15 (q, *J* = 33.9 Hz, 2F). ^{11}**B-NMR** (96 MHz, $CDCl_3$): δ = 1.41 (t, *J* = 34.5 Hz). **HRMS (ESI):** Calculated for $C_{21}H_{16}BF_2N_3$ (M+H): 360.1482; found: 360.1478.

3-Amino-5-(1-naphtalenyl)-BODIPY (6b).

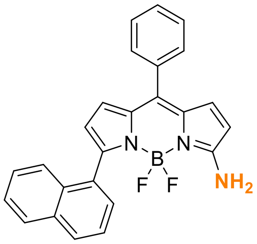

Following general method B, amino-BODIPY **6b** was isolated as an orange-red solid in 99% yield (49 mg, 0.12 mmol). **^{1}H-NMR** (300 MHz, $CDCl_3$): δ = 7.89 (t, *J* = 7.2 Hz, 3H), 7.79 (d, *J* = 7.0 Hz, 1H), 7.65 – 7.36 (m, 8H), 6.83 (d, *J* = 4.8 Hz, 1H), 6.57 (d, *J* = 3.7 Hz, 1H), 6.38 (d, *J* = 3.7 Hz, 1H), 5.88 (d, *J* = 4.9 Hz, 1H), 5.31 (s, 2H). **^{13}C-NMR** (75 MHz, $CDCl_3$): δ = 160.9, 144.7, 135.1, 134.6, 133.5, 133.3, 133.2, 133.0, 132.5, 130.4, 129.2, 128.7, 128.3, 128.2, 127.9, 126.9, 125.0, 120.7, 116.8, 112.9. **^{19}F-NMR** (282 MHz, $CDCl_3$): δ = -138.55 – (-141.46) (m, 1F), -146.58 – (-148.58) (m, 1F). **^{11}B-NMR** (96 MHz, $CDCl_3$): δ = 1.14 (t, *J* = 33.6 Hz). **HRMS (ESI):** Calculated for $C_{25}H_{18}BF_2N_3$ (M+H): 410.1639; found: 410.1640.

3-Amino-5-(9-anthracenyl)-BODIPY (6c).

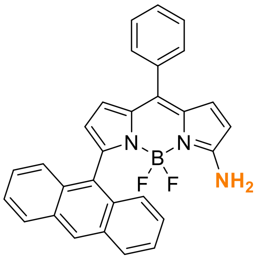

Following general method B, amino-BODIPY **6c** was isolated as an orange-red solid in 98 % yield (23 mg, 0,05 mmol). **^{1}H-NMR** (300 MHz, $CDCl_3$): δ = 8.54 (s, 1H), 8.03 (d, *J* = 8.6 Hz, 2H), 7.82 (d, *J* = 8.6 Hz, 2H), 7.69 – 7.34 (m, 9H), 6.89 (d, *J* = 4.8 Hz, 1H), 6.72 (s, 1H), 6.42 (d, *J* = 3.7 Hz, 1H), 5.97 (d, *J* = 4.5 Hz, 1H), 5.36 (s, 2H). **^{13}C-NMR** (75 MHz, $CDCl_3$): δ = 160.8, 144.8, 135.1, 134.6, 133.5, 133.3, 133.2, 133.0, 132.5, 130.4, 129.2, 128.7, 128.3, 128.2, 127.9, 126.9, 126.0, 125.8, 125.0, 120.7, 116.9, 112.9. **^{19}F-NMR** (282 MHz, $CDCl_3$): δ = -138.78 – -141.55 (m, 1F), -145.30 – -149.60 (m, 1F). **^{11}B-NMR** (96 MHz, $CDCl_3$): δ = 1.14 (t, *J* = 33.9 Hz). **HRMS (ESI):** Calculated for $C_{29}H_{20}BF_2N_3$ (M+H): 461.1797; found: 461.1673.

3-Amino-5-(9-phenanthracenyl)-BODIPY (6d).

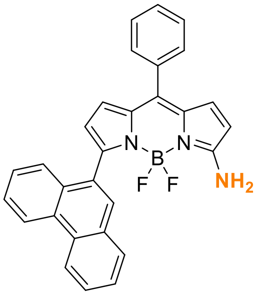

Following general method B, amino-BODIPY **6d** was isolated as an orange-red solid in 96% yield (22 mg, 0.05 mmol). ^{1}**H-NMR** (300 MHz, $CDCl_3$): δ = 8.73 (t, *J* = 7.4 Hz, 2H), 8.05 (s, 1H), 7.94 (t, *J* = 7.8 Hz, 2H), 7.76 – 7.37 (m, 9H), 6.85 (d, *J* = 4.8 Hz, 1H), 6.60 (d, *J* = 3.6 Hz, 1H), 6.41 (d, *J* = 3.7 Hz, 1H), 5.86 (d, *J* = 4.9 Hz, 1H), 5.40 (s, 2H). ^{13}**C-NMR** (75 MHz, $CDCl_3$): δ = 160.5, 148.6, 147.5, 147.4, 134.7, 134.5, 133.9, 132.3, 130.4, 129.8, 129.3, 129.1, 128.2, 127.8, 125.0, 123.2, 122.7, 122.0, 116.5, 115.62, 114.9, 112.1. ^{19}**F-NMR** (282 MHz, $CDCl_3$): δ = -140.03 – (-141.43) (m, 1F), -146.71 – (-148.23) (m, 1F). ^{11}**B-NMR** (96 MHz, $CDCl_3$): δ = 1.49 (t, *J* = 34.5 Hz). **HRMS (ESI):** Calculated for $C_{29}H_{20}BF_2N_3$ (M+H): 460.1801; found: 460.1791.

3-Amino-5-(2-methoxiphenyl)-BODIPY (6e).

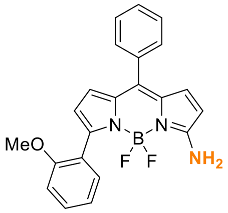

Following general method B, amino-BODIPY **6e** was isolated as an orange-red solid in 82% yield (16 mg, 0.04 mmol). ^{1}**H-NMR** (300 MHz, $CDCl_3$): δ = 8.11 – 7.97 (m, 4H), 7.69 – 7.45 (m, 5H), 7.08 – 6.97 (m, 3H), 6.88 (d, *J* = 4.9 Hz, 2H), 6.75 (d, *J* = 4.4 Hz, 1H), 3.90 (s, 3H). ^{1}**H-NMR** (300 MHz, Acetone-d_6): δ = 7.63 (d, *J* = 6.4 Hz, 1H), 7.60 – 7.47 (m, 5H), 7.33 (ddd, *J* = 8.3, 7.5, 1.7 Hz, 1H), 7.04 (d, *J* = 7.7 Hz, 1H), 6.97 (td, *J* = 7.5, 1.0 Hz, 1H), 6.90 (d, *J* = 4.9 Hz, 1H), 6.31 (dt, *J* = 8.6, 3.8 Hz, 3H), 3.76 (s, 3H). ^{13}**C-NMR** (75 MHz, $CDCl_3$): δ = 160.6, 157.9, 143.8, 134.8, 134.7, 134.0, 133.3, 132.7, 132.2, 130.4, 129.6, 129.1, 128.1, 123.7, 121.5, 119.8, 116.6, 112.3, 111.0, 55.9. ^{19}**F-NMR** (282 MHz, $CDCl_3$): δ = -144.31 (q, *J* = 32.5 Hz, 2F). ^{11}**B-NMR** (96 MHz, $CDCl_3$): δ = 1.19 (t, *J* = 34.0 Hz). **HRMS (IE):** Calculated for $C_{22}H_{18}BF_2N_3O$ (M+H): 390.1589; found: 390.1590.

3-Amino-5-(2-fluorophenyl)-BODIPY (6f).

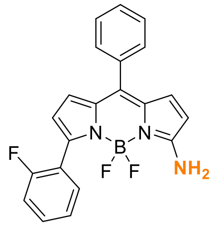

Following general method B, amino-BODIPY **6f** was isolated as an orange-red solid in 97% yield (33 mg, 0.09 mmol). **^{1}H-NMR** (300 MHz, $CDCl_3$): δ = 7.84 (td, *J* = 7.4, 1.2 Hz, 1H), 7.57 – 7.42 (m, 5H), 7.42 – 7.31 (m, 1H), 7.23 (td, *J* = 7.6, 1.2 Hz, 1H), 7.19 – 7.10 (m, 1H), 6.88 (d, *J* = 4.8 Hz, 1H), 6.52 (d, *J* = 3.8 Hz, 1H), 6.45 (d, *J* = 2.0 Hz, 1H), 6.05 (d, *J* = 4.9 Hz, 1H), 5.72 (s, 2H). **^{13}C-NMR** (75 MHz, $CDCl_3$): δ = 162.1, 161.1, 158.8, 139.8, 135.4, 134.4, 133.9, 133.8, 133.1, 132.4, 130.4, 129.8, 129.7, 129.2, 128.2, 123.3, 123.3, 122.5, 122.3, 121.2, 116.7, 115.5, 115.2, 113.2. **^{19}F-NMR** (282 MHz, $CDCl_3$): δ = -113.27 (s, 1F), -143.41 – -143.97 (m, 2F). **^{11}B-NMR** (96 MHz, $CDCl_3$): δ = 1.23 (t, *J* = 34.2 Hz). **HRMS (ESI):** Calculated for $C_{21}H_{15}BF_3N_3$ (M+H): 378.1388; found: 378.1396.

3-Amino-5-(3,4,5-trimethoxyphenyl)-BODIPY (6g).

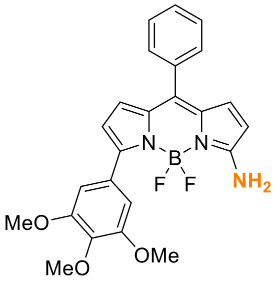

Following general method B, amino-BODIPY **6g** was isolated as an orange-red solid in 72% yield (13 mg, 0.03 mmol). **^{1}H-NMR** (300 MHz, $CDCl_3$): δ = .48 (d, *J* = 2.5 Hz, 5H), 7.11 (s, 2H), 6.85 (d, *J* = 4.8 Hz, 1H), 6.47 (d, *J* = 3.8 Hz, 1H), 6.40 (d, *J* = 3.8 Hz, 1H), 6.05 (d, *J* = 4.8 Hz, 1H), 5.81 (s, 2H), 3.91 (d, *J* = 1.1 Hz, 9H). **^{13}C-NMR** (75 MHz, $CDCl_3$): δ = 160.9, 152.6 (2C), 147.8, 137.7, 134.9, 134.5, 134.4, 133.6, 132.7, 130.4 (2C), 130.0, 129.1, 128.2 (2C), 121.8, 115.3, 113.0, 106.5, 60.9, 56.1 (2C). **^{19}F-NMR** (282 MHz, $CDCl_3$): δ = -141.77 (q, *J* = 33.3 Hz, 2F). **^{11}B-NMR** (96 MHz, $CDCl_3$) δ = 1.42 (t, *J* = 33.9 Hz). **HRMS (ESI):** Calculated for $C_{24}H_{22}BF_2N_3O_3$ (M+H): 450.1779; found: 450.1796.

3-Amino-5-(2-benzo[b]thienyl)-BODIPY (6h).

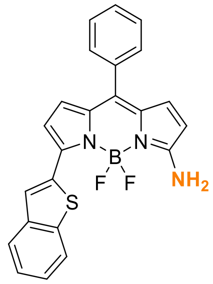

Following general method B, amino-BODIPY **6h** was isolated as an orange-red solid in 83% yield (32 mg, 0.08 mmol). **^{1}H-NMR** (300 MHz, $CDCl_3$): δ = 8.09 (s, 1H), 7.80 (dd, *J* = 16.7, 1.5 Hz, 2H), 7.48 (s, 5H), 7.32 (dt, *J* = 6.2, 5.6 Hz, 2H), 6.86 (d, *J* = 4.8 Hz, 1H), 6.67 (d, *J* = 4.0 Hz, 1H), 6.47 (d, *J* = 4.0 Hz, 1H), 6.07 (d, *J* = 4.8 Hz, 1H), 5.85 (s, 2H). **^{13}C-NMR** (75 MHz, $CDCl_3$): δ = 160.9, 141.0, 140.3, 139.7, 135.4, 135.1, 134.4, 133.5, 133.2, 130.4, 129.3, 128.2, 117.0 (s), 113.1. **^{19}F-NMR** (282 MHz, $CDCl_3$): δ = -144.25 (q, *J* = 34.1 Hz, 2F). **^{11}B-NMR** (96 MHz, $CDCl_3$): δ = 1.50 (t, *J* = 34.3 Hz). **HRMS (IE):** Calculated for $C_{23}H_{16}BF_2N_3S$ (M+H): 416.1202; found: 416.1201.

3-Amino-5-(2-thienyl)-BODIPY (6i).

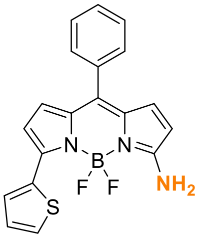

Following general method B, amino-BODIPY **6h** was isolated as an orange-red solid in 87% yield (19 mg, 0.05 mmol). **^{1}H-NMR** (300 MHz, $CDCl_3$): δ = 7.81 (d, *J* = 3.7 Hz, 1H), 7.47 (s, 5H), 7.30 (dd, *J* = 5.1, 1.1 Hz, 1H), 7.11 (dd, *J* = 5.1, 3.7 Hz, 1H), 6.84 (d, *J* = 4.9 Hz, 1H), 6.55 (d, *J* = 4.0 Hz, 1H), 6.45 (d, *J* = 3.9 Hz, 1H), 6.06 (d, *J* = 4.8 Hz, 1H), 5.76 (s, 2H). **^{13}C-NMR** (75 MHz, $CDCl_3$): δ = 160.6, 140.9, 135.8, 134.8, 134.6, 134.5, 133.7, 132.8, 130.4, 129.2, 128.1, 127.4, 127.3, 127.2, 125.6, 122.4, 116.1, 112.4. **^{19}F-NMR** (282 MHz, $CDCl_3$): δ = -144.23 (q, *J* = 34.3 Hz, 2F). **^{11}B-NMR** (96 MHz, $CDCl_3$): δ = 1.43 (t, *J* = 34.7 Hz). **HRMS (IE):** Calculated for $C_{19}H_{14}BF_2N_3S$ (M+H): 366.1046; found: 366.1052.

6. UV-Vis/EMISSION SPECTRA

Absorbance spectrum of azo BODIPY **5a-i** and absorbance and emission spectrum of amino BODIPY **6a-i**.

Figure S1 Absorbance spectrum of azo BODIPY **5a-i**.

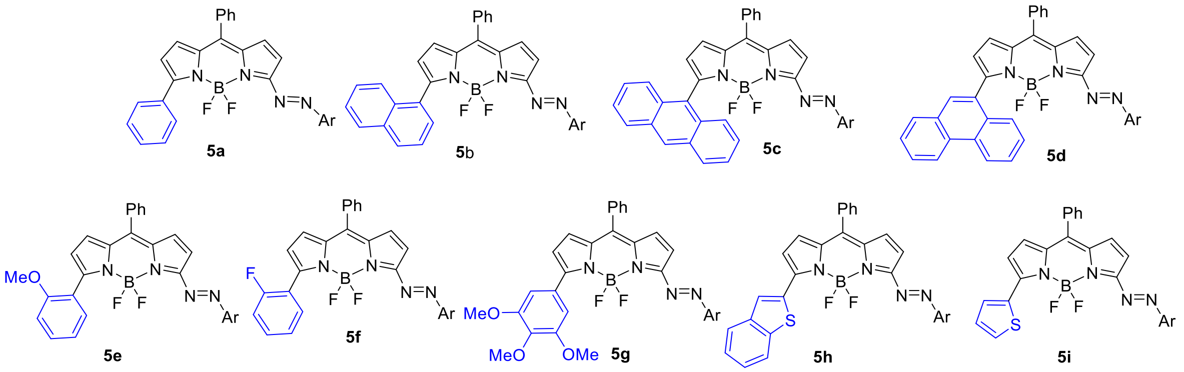

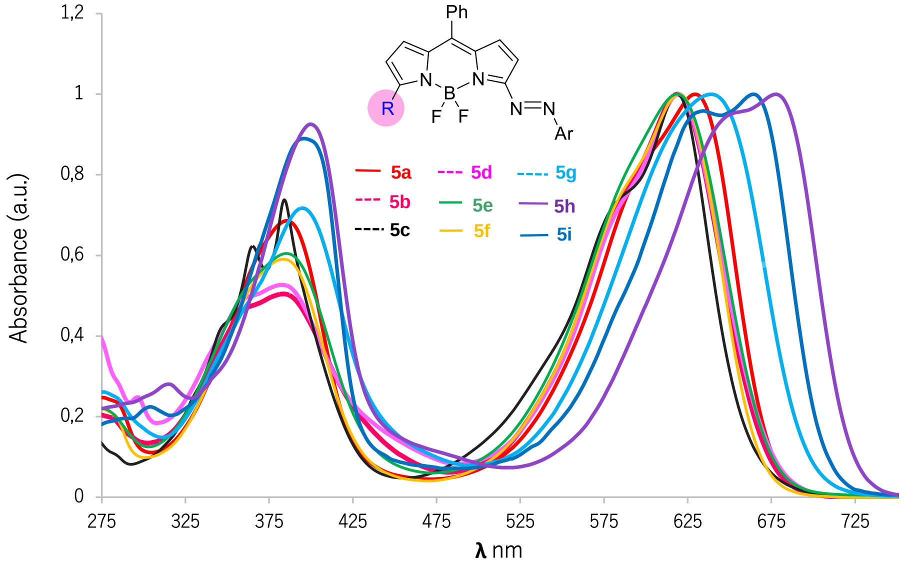

Figure S1 Absorbance spectrum of azo BODIPY **5a-i**.

Table S2. Absorption spectra data of azo-BODIPY **5a-i**.[a]

Entry	R	5	λ_{abs} (nm)[b]	ε ($cm^{-1}M^{-1}$)
1	Ph	**5a**	630	45047
2	Naphthyl	**5b**	620	37064
3	anthracenyl	**5c**	620	63412
4	phenanthryl	**5d**	620	31974
5	2-MeOPh	**5e**	619	58367
6	2-F-Ph	**5f**	621	48326
7	3,4,5-$(MeO)_3$Ph	**5g**	640	58321
8	2-benzothienyl	**5h**	679	29122
9	2-thienyl	**5i**	665	20815

[a]DCM used as solvent 10^{-5} M. [b] Maximum absorption wavelength.

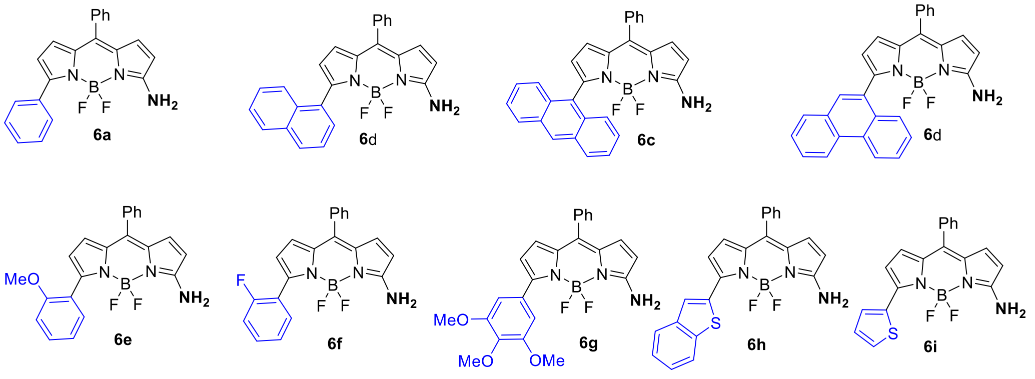

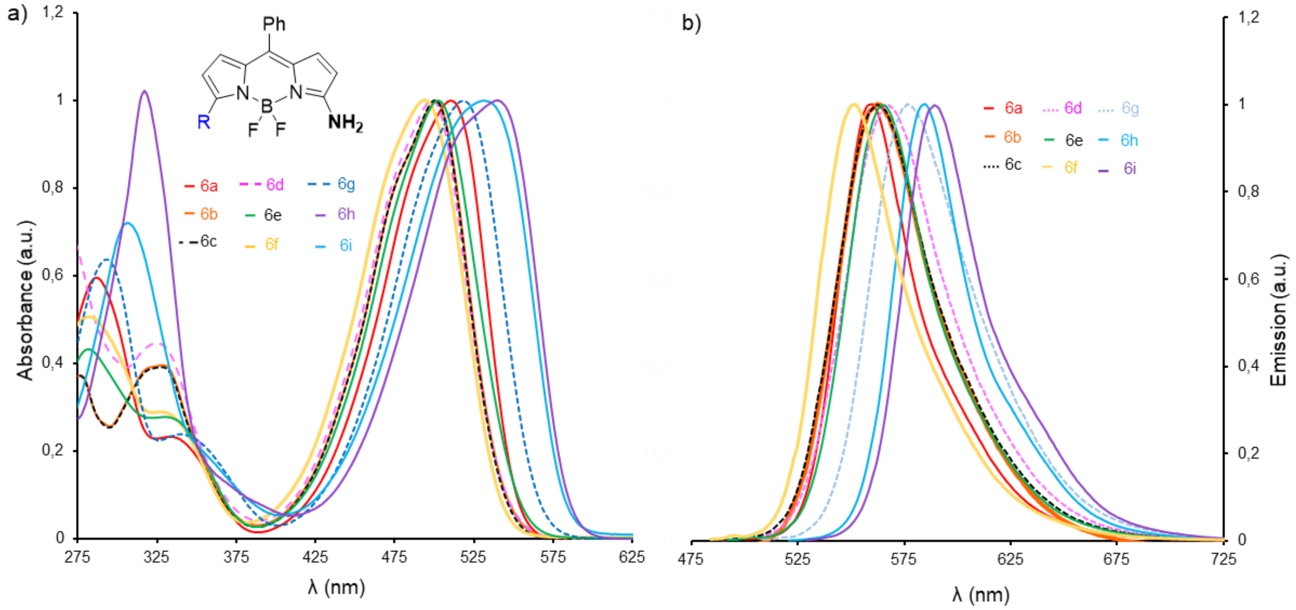

Figure S2 a) Absorbance spectrum of **6a-i**. b) Emission spectrum of **6a-i**.

Table S3. Photophisical data of amino-BODIPY **6a-i**.[a]

Entry	6	λ_{abs} nm[b]	ε ($cm^{-1}M^{-1}$)	λ_{em} (nm)[c]	Φ[d]	Stoke shift (nm)
1	**6a**	511	35694	560	0.55	50
2	**6b**	500	30676	562	0.33	63
3	**6c**	500	37830	562	0.27	63
4	**6d**	499	32008	567	0.47	68
5	**6e**	502	35854	565	0.24	63
6	**6f**	493	30833	552	0.27	59
7	**6g**	517	31106	576	0.7	59
8	**6h**	531	32344	589	0.91	64
9	**6i**	525	26645	585	0.95	54
10	**6i**[e]	500	26645	585	0.4	67

[a] DCM used as solvent (10^{-5} M). [b] Maximum absorption wavelength.. [c] Emission upon excitations at the maximum λ_{abs}. [d] Rhodamine 101 was used as standard. [e]DMSO/PBS used as solvent.

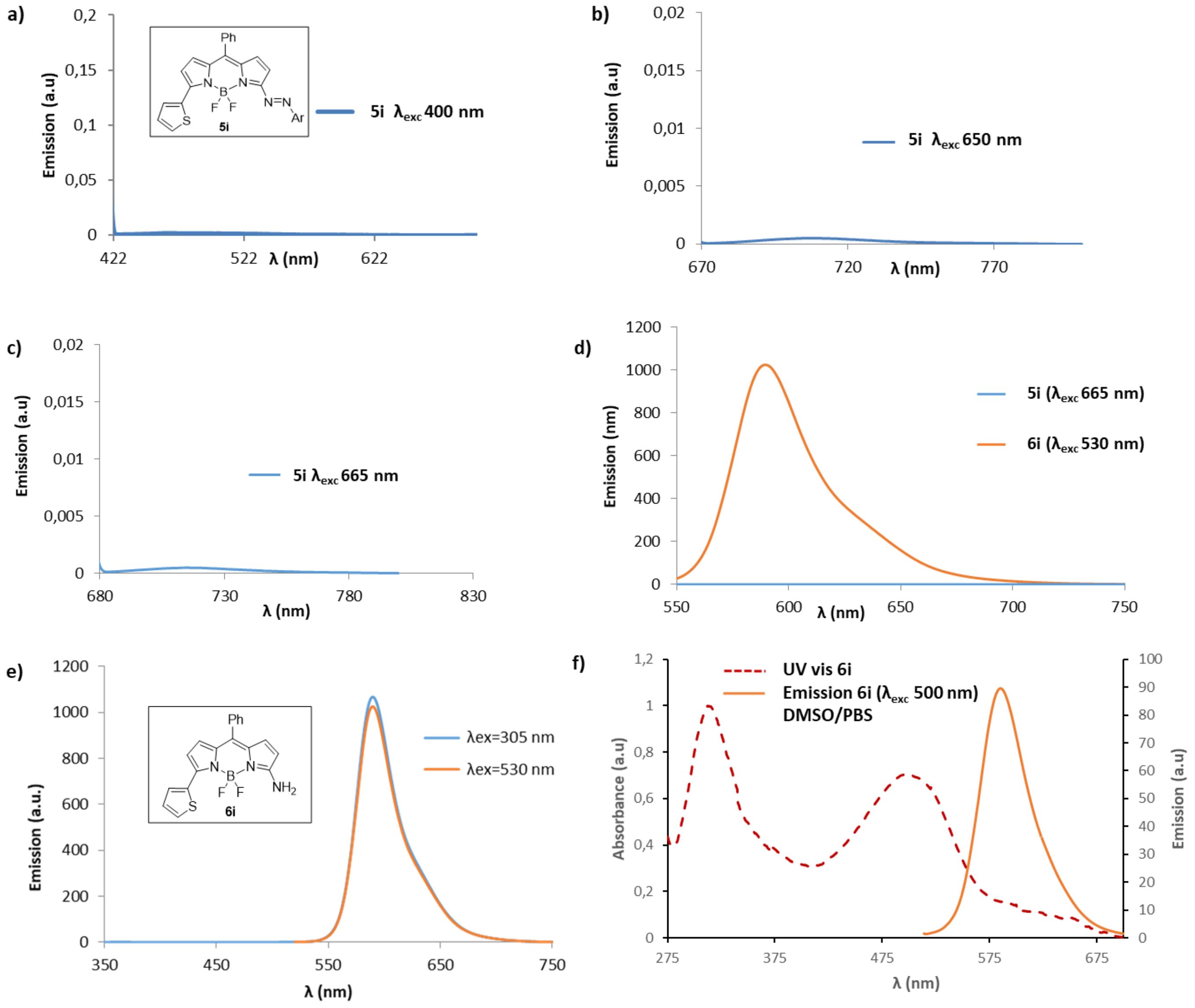

Figure S3 a-c) Experimental emission spectrua of azo-BODIPY **5i** [10^{-5} M in DCM] upon excitation at λ_{ex} 400, 650 and 665 nm respectively. **d**) Comparison of emission spectra of azo-BODIPY **5i** and amino-BODIPY **6i** (λ_{ex} 500 nm). **e**) Emission spectrum of amino-BODIPY **6i** [10^{-5} M in DCM] upon excitation at λ_{ex} 305 nm (S_3) or λ_{ex} 530 nm (S_1); λ_{em} at 590 nm. **f**) Normalized UV-vis (red dot-line) and emission (orange line) spectra of amino-BODIPY **6i** [using PBS/DMSO as solvent (10^{-7} M, ratio 4:1 respectively)], **6i** λ_{em} 585 nm exciting at λ_{ex} 500 nm.

6.1 Fluorescence Quantum Yields

Quantum yields were determined by measuring both absorbance and fluorescence of amino-BODIPY **6** and Rhodamina 101 in EtOH (Rhodamina 101 as standard $\Phi r = 1$).[5] The measurements were performed using 10×10 mm cuvettes on non-degassed samples. Quantum yields were determined in DCM for all amino-BODIPY **6** and also in DMSO/PBS for **6i**. For the relative determination of the fluorescence quantum yield (Φ), the following formula was used:[6]

$$\Phi_x = \Phi_r \text{ x } \frac{F_x}{F_r} \text{ x } \frac{1-10^{-A_r(\lambda_{ex})}}{1-10^{-A_x(\lambda_{ex})}} \text{ x } \frac{n_x^2}{n_r^2}$$

Subscripts ***x*** and ***r*** refer to sample and reference (standard) fluorophore respectively with known quantum yield **Φ*r*** in a specific solvent

F stands for the spectrally corrected, integrated fluorescence spectra.

***A*(λ_{ex})** denotes the absorbance at the used excitation wavelength λ_{ex}.

n represents the refractive index of the solvent (at the average emission wavelength).

To minimize inner filter effects, the absorbance at the excitation wavelength λ_{ex} was kept under 0.1.

7. COMPUTATIONAL STUDIES

7.1 Computational details

Ground state geometry optimizations for the azo-BODIPY **5i** and the amino-BODIPY **6i** were performed at the wB97X-D/cc-pVDZ[7,8] level of theory, in a continuum of dichloromethane under the Polarizable Continuum Model (PCM) using the integral equation formalism variant (IEFPCM).[9,10,11] Subsequent frequency calculations to check the nature of the stationary points found were undertaken at the same level of theory. These calculations were done with the Gaussian 16 (Revision C.01).[12]

The rotational flexibility of the **5i** derivative requires a conformational analysis to identify the most stable conformer(s) at thermal equilibrium, for which we will model the photophysics. The most stable conformers differing in the orientation of the thiophene and azo groups relative to the BODIPY moiety were scrutinized. In turn, the effect on the internal energy of the rotation of the methoxy substituent around the $C_{Ar}O$ bond was also evaluated. The geometries, relative energies in solution and population according to a Boltzmann distribution at 298 K of the 8 most stable conformers are collected in Figure S4. The two most stable species (conformers D, 51% and C, 36%) have in common the layout of the three chromophores while differ in the dihedral of the methoxy group. Since the rotation of the OCH_3 substituent around the $C_{Ar}O$ bond is not expected to significantly change the ground and excited potential energy landscape, all the subsequent electronic structure calculations were performed on the major D conformer.

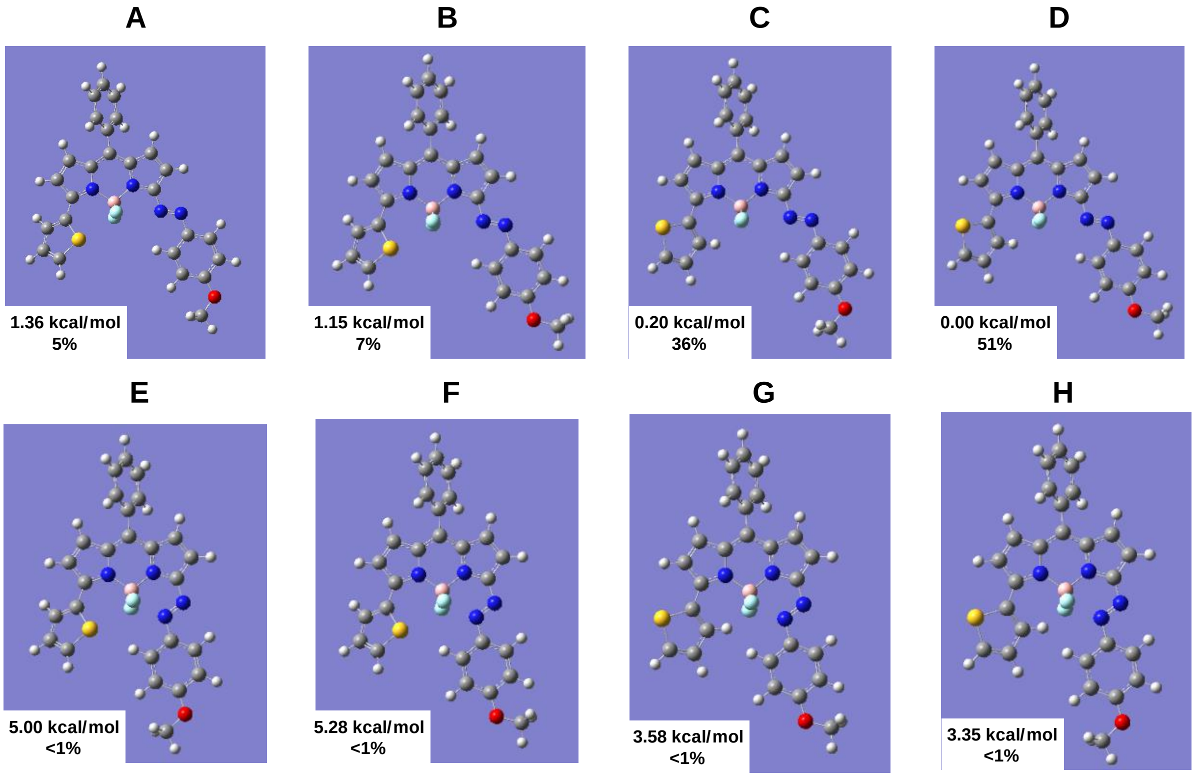

Figure S4 Conformational analysis for the *trans*-**5i** derivative. Energies are calculated at the wB97X-D/cc-pVDZ level of theory, in a continuum of dichloromethane under the IEFPCM formalism.

For the D conformer of **5i** and **6i**, gas phase vertical excitation energies were calculated at the RI-ADC(2)/def2-SVP[13,14,15,16,17,18] level of theory with Turbomole 7.02.[19]
Natural transition orbitals (NTOs) and excitation analysis was carried out with the TheoDORE package.[20]
Excited state geometry optimizations for minima, transition states and conical intersections were undertaken at the RI-ADC(2)/def2-SV(P) level of theory, using in the latter case SHARC[21,22] as an interface between Turbomole, for energy and gradient calculations, and Orca 4.1, as a geometry optimizer.[23]
Additionally, for benchmarking purposes, gas phase S_1 vertical excitation energies for azo-BODIPY **5i** and for amino-BODIPY **6i** were also respectively computed at the MS3-CASPT2(8,6)/ANO-S-VDZ and MS5-CASPT2(12,10)/ANO-S-VDZ levels of theory with the OpenMolcas code.[24]
For the **5i** derivative, the CASPT2 S_1 excitation energy of 1.91 eV (640 nm) is in excellent agreement with the experimental absorption spectrum (585 nm, see Table S2). Additionally, a good agreement between the CASPT2 and the ADC(2) levels of theory was found for the S_1 excited state energy (ΔE~0.3 eV). It is particularly interesting that the state order is kept at both levels of theory, being the S_1 ($\pi\pi^*$) the first bright state, S_2 ($n\pi^*$) the dark state and S_3 ($\pi\pi^*$) the second bright state.
According to the CASPT2 approach, the S_1 state in **6i** absorbs at 2.29 eV (541 nm), also in good agreement with both experimental data and ADC(2) calculations.

7.2 Reactive ground and excited PES for azo-BODIPY 5i

For the **5i** derivative, three different ground state minima were found: *trans*, *cis* bottom and *cis* top, with relative energies 0, 0.51 and 0.51 eV, respectively (see Figure S5). The two *cis* isomers differ in the relative orientation of the azobenzene moiety with respect to the BODIPY resulting in positive (*cis* bottom) or negative (*cis* top)

helicities. These minima are connected to the *trans* isomer through energy barriers of ~1.4 eV (~32 Kcal/mol) which should avoid fast thermal isomerization at room temperature. These transition states (TS^{top} and TS^{bottom}, see Figures S5 and S9) show geometries close to those of the reactive S_1/ S_0 conical intersections: CI^{top}-$S_1(\pi\pi^*)/S_0$ and CI^{bottom}-$S_1(\pi\pi^*)/S_0$.

Figure S5 shows both possible reactive pathways starting from the *trans* isomer, and evolving either towards the *cis* top or the *cis* bottom isomer. For clarity, and due to the symmetry of the S_1 and S_0 potential energy surfaces along the isomerization coordinate, only the potential energy landscape connecting the *trans* and *cis* bottom isomer is shown in Figure 4 of the main manuscript. The unreactive pathway can be also found in Figure 4 (a) of the main manuscript.

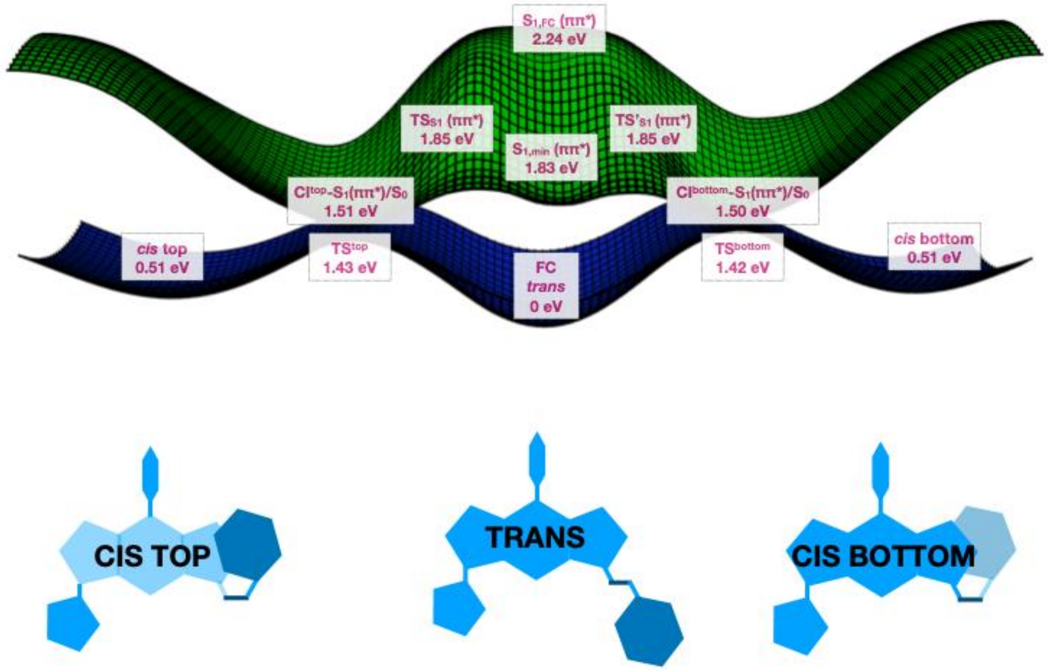

Figure. S5 Ground and excited potential energy surface showing the reactive/*trans-cis* isomerization pathway for the azo-BODIPY **5i** derivative at the RI-ADC(2)/def2-SVP level of theory.

7.3 Calculated absorption spectra and NTOs (Natural Transition Orbitals)

a) *trans*-**5i** azo-BODIPY

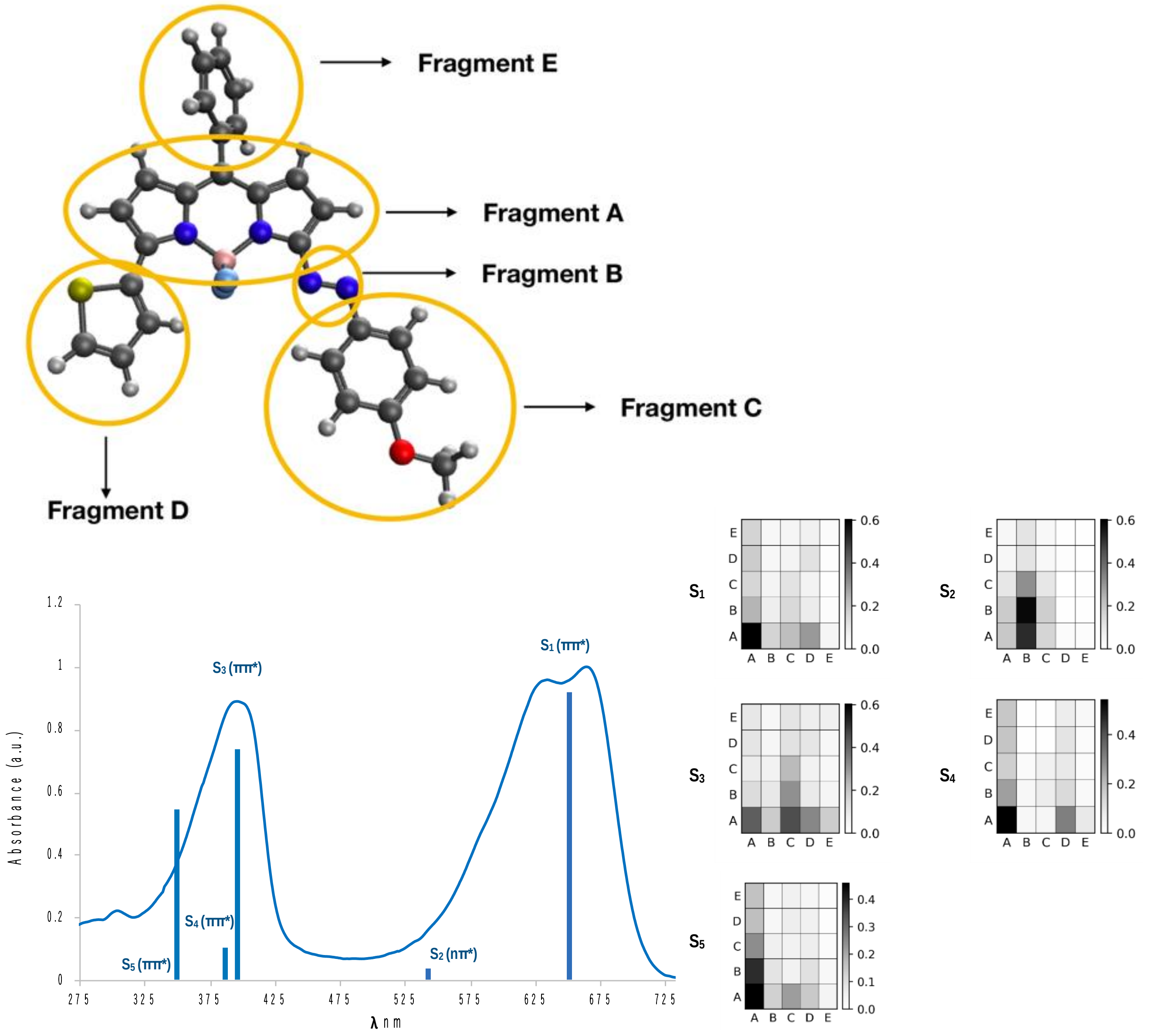

Figure S6 (Top) Fragment decomposition used for excitation analysis (see also Fig 2 manuscript). (Bottom-left) RI-ADC(2)/def2-SVP vertical excitation energies superimposed to the experimental absorption spectrum for the *trans* isomer of the azo-BODIPY **5i** derivative and (bottom-right) electron-hole correlation plots, obtained at the RI-ADC(2)/def2-SVP level of theory, for isomer *trans*-**5i** azo-BODIPY derivative where the horizontal and vertical axes indicate the origin and fate of the electronic transitions responsible for the lowest energy region of the absorption spectrum. Associated NTOs in Figure 2 (manuscript).

b) *cis*-top azo-BODIPY **5i**

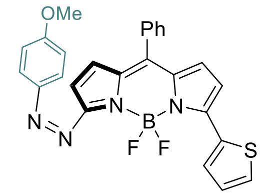

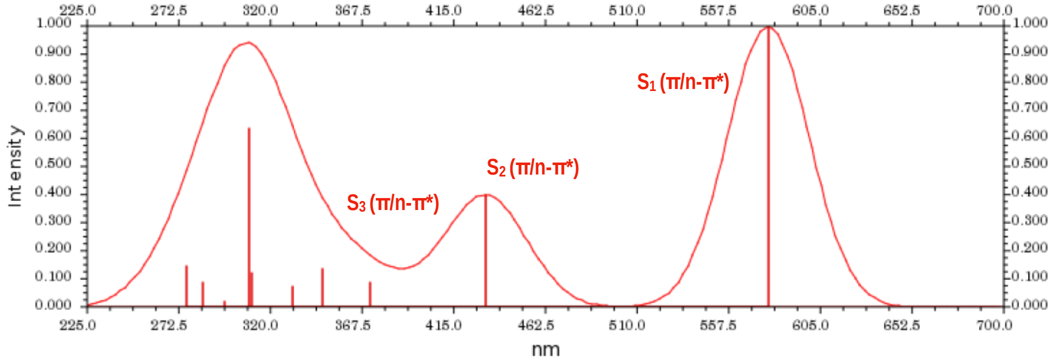

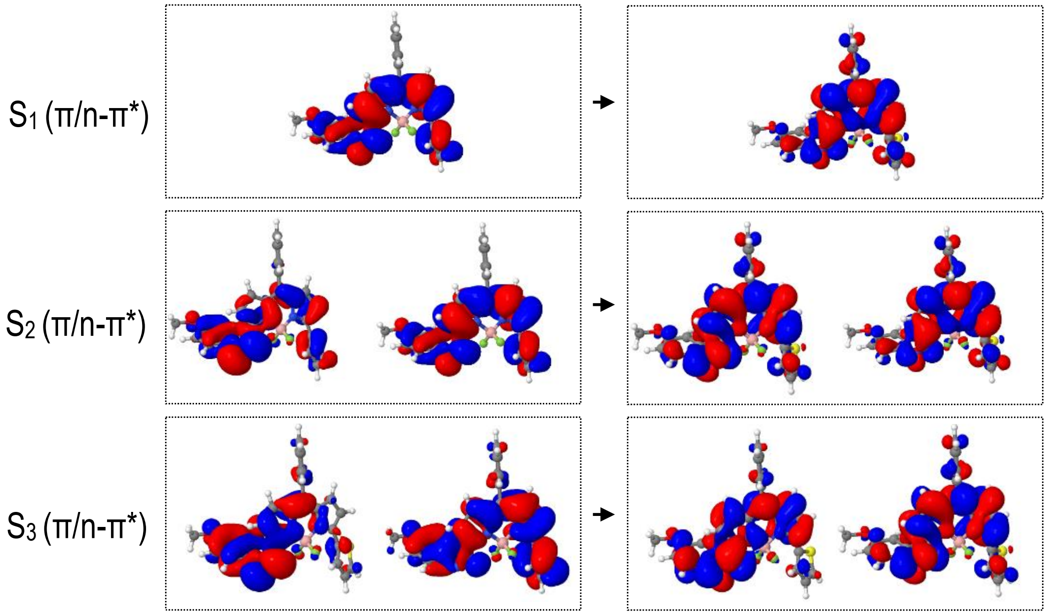

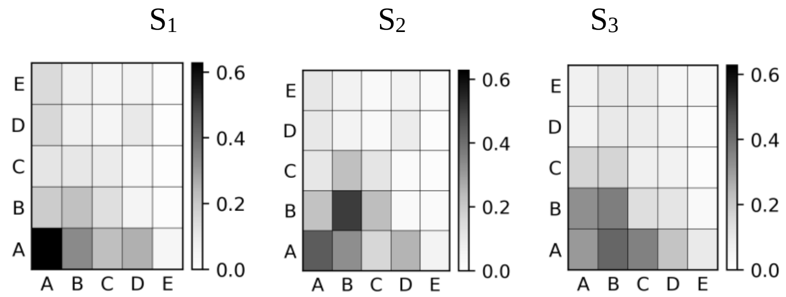

Figure S7 (Top) Computed absorption spectrum for the *cis*-top isomer of the azo-BODIPY **5i** derivative obtained at the RI-ADC(2)/def2-SVP level of theory (FWHM 20nm), (Middle) NTOs involved in the S_1, S_2 and S_3 electronic excited states (for clarity of visualization the azo compound has been rotated 180º along the z-axis). (Bottom) Electron-hole correlation plots where the horizontal and vertical axis respectively indicate the origin and fate of the electronic transitions obtained at same level of theory (The A-E fragments as shown in Figure S6).

b) *cis*-bottom azo-BODIPY**5i**

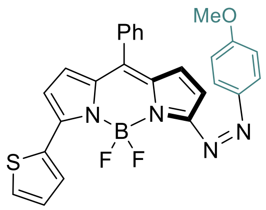

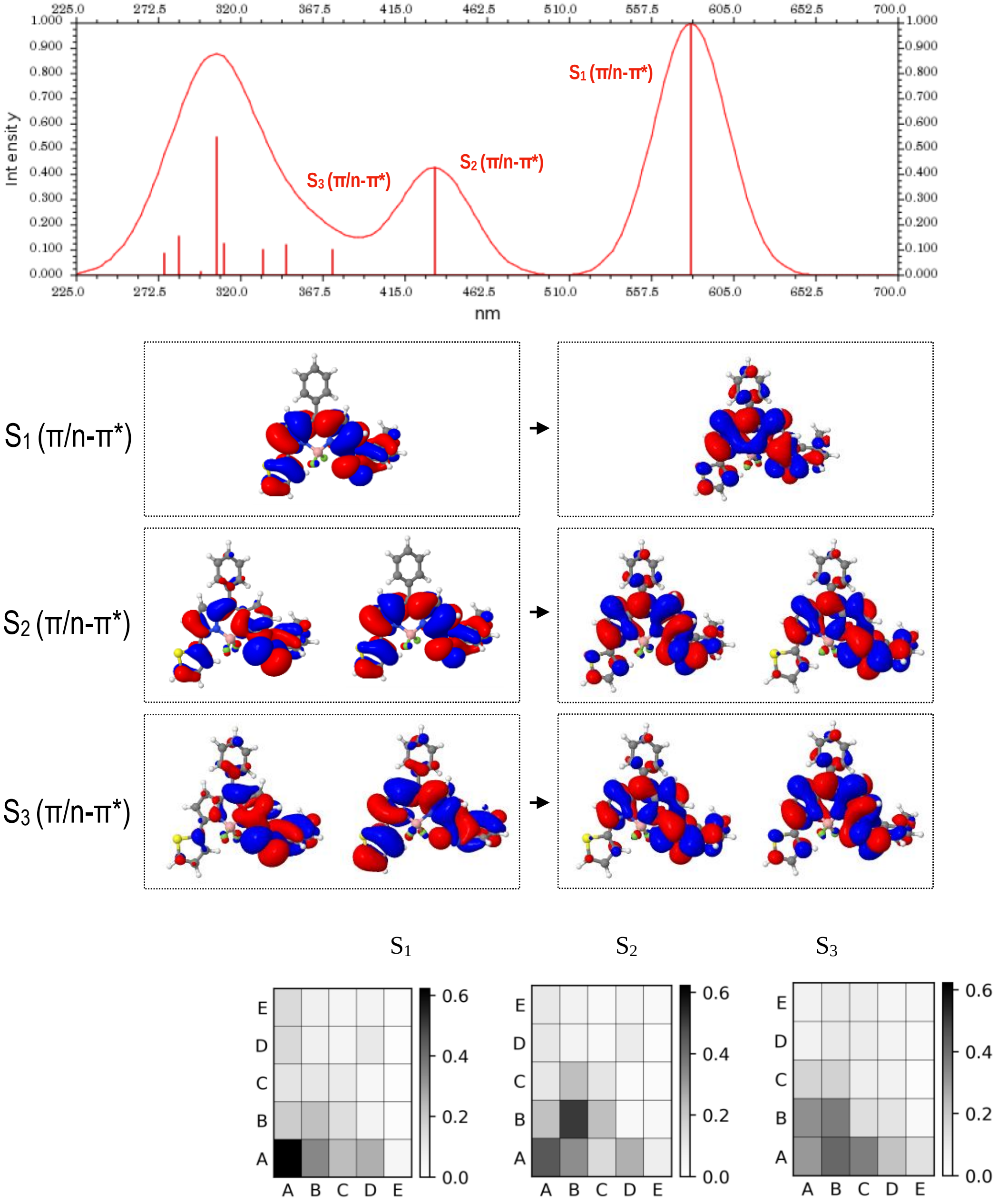

Figure S8 (Top) Computed absorption spectrum for the *cis*-bottom azo-BODIPY **5i** obtained at the RI-ADC(2)/def2-SVP level of theory (FWHM 20nm). (Middle) NTOs involved in the S_1, S_2 and S_3 electronic excited states. (Bottom) Electron-hole correlation plots where the horizontal and vertical axis respectively indicate the origin and fate of the electronic transitions obtained at same level of theory (The A-E fragments as shown in Figure S6).

7.4 Stationary geometries 5i

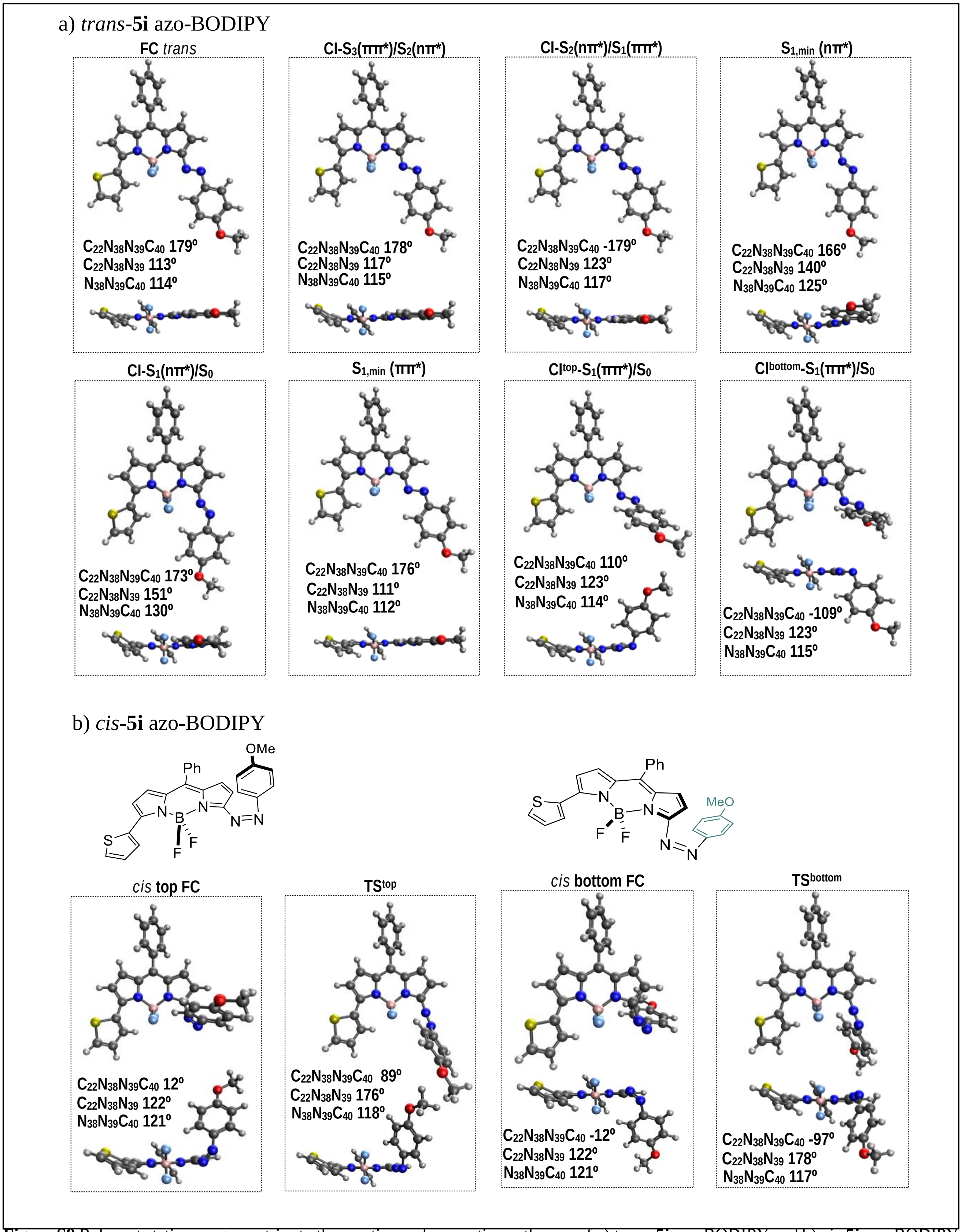

Figure S9 Relevant stationary geometries to the reactive and unreactive pathways of a) *trans*-**5i** azo-BODIPY and b) *cis*-**5i** azo-BODIPY (*cis*-top and *cis*-bottom).

7.5 Stationary geometries 6i

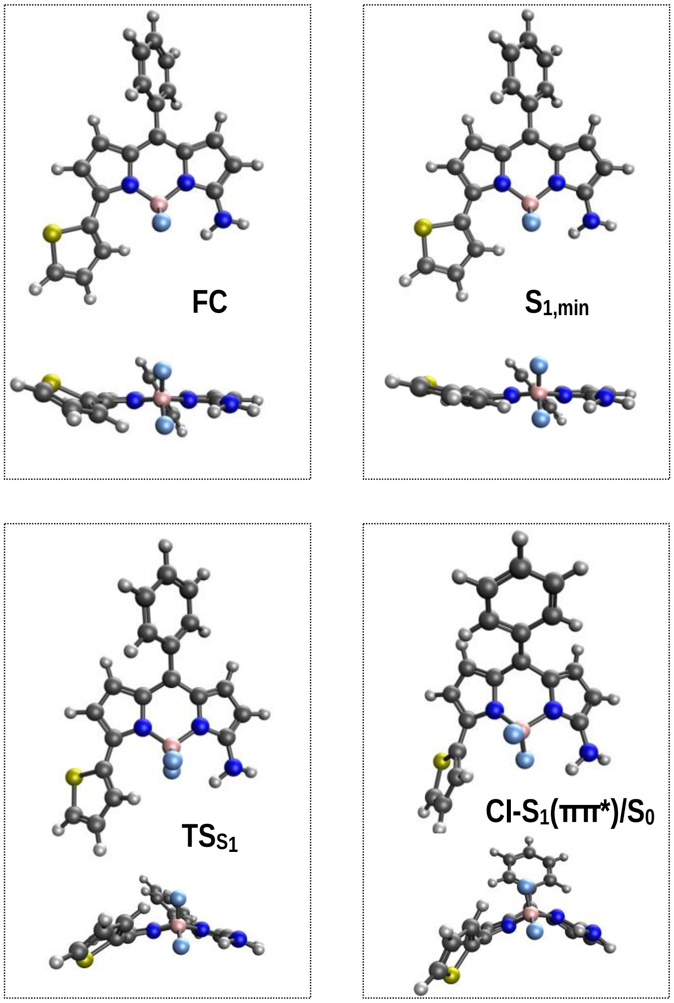

Figure S10 Relevant stationary geometries to the deactivation mechanism of the 3-amino-BODIPY **6i**.

8. ENZYMATIC AZO BOND CLEAVAGE STUDIES

Purified human NAD(P)H: quinone oxidoreductase 1 (NQO1 human)[25] was obtained from Sigma Chem. Co (St Louis Mo) (Catalogue No. SRP6539). The bacterial azoreductase from Bacillus cereus was produced and purified in the laboratory from the gene *azoRBC* that contains the sequence encoding the enzyme and the sequence encoding a poly-His tail per enzyme subunit. This gene was supplied by Genescript, NJ.

The enzymatic studies were carried out using a Spectrophotometer JASCO v730 (Tokyo, Japan) with a spectrophotometric cell provided with magnetic stirring.

8.1 BACTERIAL AZOREDUCTASE (*azoRBC*)

a) Expression, production and purification of *azoRBC* from the microorganism bacillus cereus

The gene coding for the *azoRBC* enzyme was cloned into a plasmid pET28b to be expressed recombinantly in the E. *coli* BL21 (DE3) organism. The expression was carried out in a ZY autoinduction medium containing 30 µg/mL of kanamycin at 37 ºC for 16 h. The cells were harvesting by centrifugation at 4000 rpm for 30 min. Cell pellets were suspended in 50 mM sodium phosphate buffer, 150 mM NaCl, 5 mM Imidazole and 3 mM benzamidine, pH 7.4 and lysed by means of 10 seconds ON/OFF sonication cycles at an amplitude of 20 % in an ice-water bath. Then, the lysate was centrifuged for 30 minutes at 14000 rpm. The pellet was discarded, and the protein was purified by an imidazole gradient using an agarose column IDA-Ni^{2+}. The poly-histidine domain present at the N-terminus of each sub-unit strongly adsorbs on the metal chelate. The native proteins were eluted with 100 mM imidazole. Then, the recombinant enzyme was eluted with sodium phosphate buffer (50 mM), NaCl (150 mM), and Imidazole (250 mM) (Figure S11).

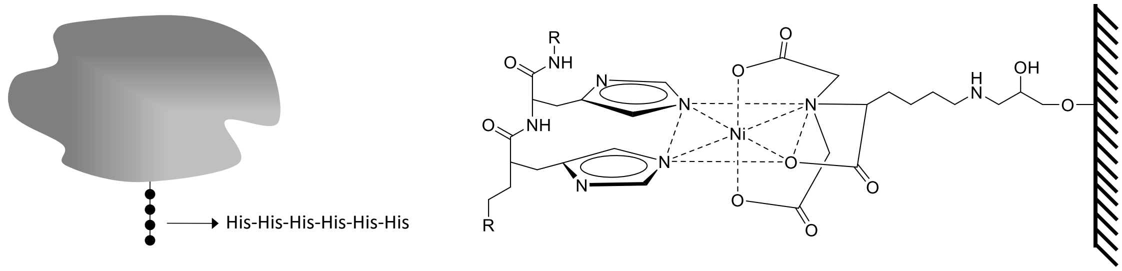

Figure S11 Azoreductase purification: The plasmid containing the gene encoding the enzyme was expressed in Escherichia coli BL21. To purify the enzyme, a plasmid was acquired with its gene to which 6 histidine residues had been added at the amino terminal. The enzyme was purified by selective adsorption on supports activated with metal chelates (agarose column IDA-Ni^{2+}).

Finally, the pure enzyme was dialyzed against 25 mM sodium phosphate pH 7 and stored at 4 °C. Protein concentration was determined by Pierce® BCA kit and protein purity was checked by SDS-PAGE (Sodium Dodecyl Sulfate PolyAcrylamide Gel Electrophoresis) (Figure S12).

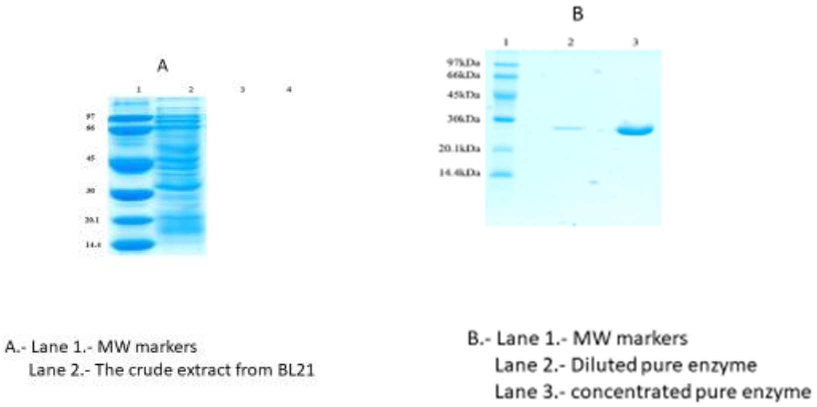

Figure S12 SDS-PAGE electrophoresis. The denatured proteins (boiled for 5 min in the presence of SDS) were fully unfolded. The hydrophobic pockets were loaded in the SDS anionic surfactant and acquired a similar large negative charge for all proteins. These proteins were electrophoresed through a porous polyacrylamide gel: Proteins move (between a negative pole and a positive pole) through the gel according to their size (smaller ones move faster, while larger ones move at a slower rate). **A**) SDS of E. coli BL21: lane 1: MW markers; lane 2: Crude extract from BL21. **B**) SDS of pure azoreductase: lane 1: MW markers; lane 2: diluted pure enzyme; lane 3: concentrated pure enzyme. The presence of a single band on the gel indicates that the protein was completely pure.

8.2 Preparation of *i-azoRBC*: Enzymatic immobilization on agarose PEI

Bacterial *azoRBC* was immobilized on a solid support (agarose gel) coated with polyethyleneimine (PEI). The enzyme was immobilized by an adsorption method promoted by the ionic exchange between several carboxyl groups of each enzyme molecule and several ionized amino groups of a polyethyleneimine-coated agarose support. Thus, the highly hydrophilic environment surrounding each enzyme molecule can also protect it from other negative effects promoted by the presence of ethanol that is necessary to solubilize substrates and products. The immobilized enzyme on a solid support was used and filter from the reaction medium, retaining the biocatalyst in a porous filter as soon as the reaction was finished. In this manner, the immobilize enzyme avoided the generation of aggregates that inactivated the enzyme.

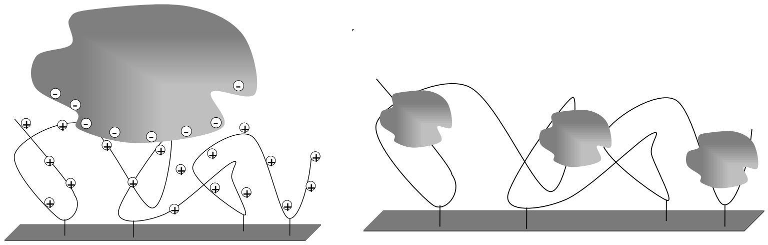

Fig. S13 Representation of the immobilized enzyme on agarose PEI support.

Two milliliters of diluted azo-RBC (0.5 mg/mL) with 60 mL of TRIS buffer (25 mM in water, pH 7), was added 3 g of agarose 6B support (6%). The agarose support had the surface completely covered with polyethyleneimine (PEI) of 25000 Da molecular weight. The suspension was gently stirred to avoid breaking the particles from the solid support.
The initial activity of the solution (before adding the support) was **0.18 Δ Abs /min** using 50 µL of the enzyme solution. The disappearance of the activity of the supernatant indicates the percentage of enzyme that is adsorbed on the solid support. On the other hand, the activity of the suspension indicates the percentage of activity that the enzyme retains after its immobilization. As it can be seen in Figure S14, the enzyme is completely immobilized

in less than 1 h and that the immobilized enzyme retains 100% of the activity. The derivative contains 0.33 mg of enzyme per gram of catalyst.

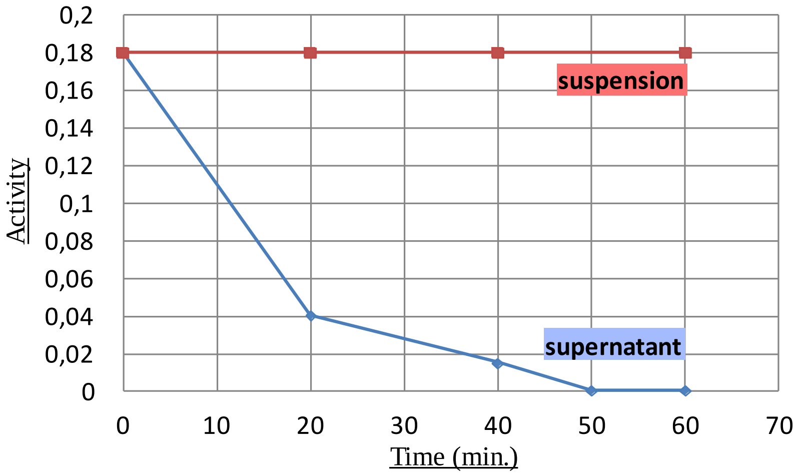

Figure. S14 Time-course of immobilization of bacterial azoreductase azo-RBC on PEI-agarose. The enzyme is 100% immobilized (on polyethylenimine agarose) in 50 minutes. Activity measurements based on activity of the supernatant (blue line) and the suspension (red line) solutions during the immobilization studies. The immobilized enzyme (suspension, red line) retains 100% of the activity corresponding to the soluble enzyme used for the immobilization.

8.3 Enzymatic Activity and Stability studies

The standard activity of bacterial reductase *azoRBC* has been measured as a free *azoRBC*, and also during the immobilization process. Likewise, the standard activity has been also measured of the immobilized enzyme *i-azoRBC* and the human NQO1 enzyme. In all cases, Methyl Red which is a fairly water soluble azo derivative, was used as a reference. The concentration of Methyl Red could be increased a tenfold factor establishing as the *standard reductase activity assay* 50 µL of a 1 mg/mL solution of Methyl Red.

a. ACTIVITY OF *i-azoRBC*

The activities of soluble (*azoRBC*) and immobilized azoreductase (***i-****azoRBC*) were measured in fully aqueous solution (buffer solution) and in a 1:1 water/ethanol solution. The assay followed the *standard reductase activity assay* using fully aqueous conditions (2 mL of buffer solution) or 50% ethanol (1 mL of ethanol/1 mL of buffer solution) and Methyl Red as a reference. The immobilized enzyme was assayed by using a stirring bar in the spectrophotometric cell in order to maintain the immobilized derivative perfectly suspended in the reaction mixture.
The comparative results are shown in the following table.

Table S4: Enzyme activity of *azoRBC* and immobilized *i-azoRBC*

	**Enzyme activity Δ Abs** (430 nm)/min (%)[a]	
Reaction conditions	Free ***azoRBC***	***i-azoRBC***
Fully aqueous	0.18 (100%)	0.18 (100%)
50% ethanol	**0.006 (3%)**	**0.036 (20%)**

[a] Considering 0.18 Abs/min the 100% see Figure S14

The immobilized enzyme (*i-azoRBC*) was approximately six times more active in the presence of ethanol than the free enzyme. This result could not only be related to aggregation phenomena but could also be due to the direct effect of ethanol, which decreases the enzymatic activity of the free enzyme. The presence of PEI in the immobilized derivative can reduce the effective concentration of ethanol in the surroundings of each immobilized enzyme molecule.

b. ENZYMATIC STABILITY STUDIES of *i-azoRBC*

The stability of the immobilized azoreductase and the free enzyme (Figure S15) was studied using 1 mL of the corresponding enzyme (free or immobilized) diluted with 5 mL of buffer solution and 5 mL of ethanol at pH 7.0 (25 mM TRIS buffer). At different times, samples (200 µL) of each of the suspensions were withdrawn and activities were measured and compared with those that were obtained in the absence of ethanol. The activity was measured according to the *standard reductase activity assay* (50 µL of a 1 mg/mL solution of Methyl Red). The immobilized enzyme is shown to be more stable to ethanol than the soluble enzyme without immobilization.

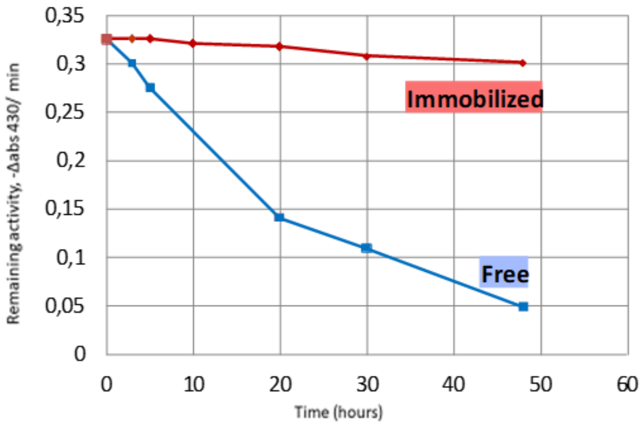

Fig. S15 Time-course of inactivation of soluble (blue line) and immobilized (red line) enzyme *azoRBC* in the presence of 50% ethanol (mixed with 25 mM TRIS buffer at pH 7.0) at 25°C.

8.4 ENZYME ASSAYS

Enzyme assays in FULLY AQUEOUS MEDIA (human or bacterial enzymes)

ENZYMES SOLUTIONS

A solution of bacterial enzyme (*azoRBC*) was prepared by diluting 10 µg of purified enzyme in 10 mL of TRIS 50 mM of pH 8.0. Similarly, a commercial sample of 10 µg of human NQO1 was dissolved in 10 mL of TRIS 50 mM of pH 8.0. The enzyme solutions (1 µg/mL) were stored at 4 ^{o}C.
To a spectrophotometer cuvette was added 2 mL of TRIS buffer (50 mM, pH 7.0), 200 µL of previously prepared bacterial *azoRBC* or human NQO1 solutions (1 µg/mL dissolved in 50 mM TRIS buffer of pH 8.0), and 200 µL of substrate (Methyl Red as the reference azocompound or the corresponding azo-BODIPY **5**) (25 µg/mL in ethanol) at 25°C. At this point, the absorbance of the reaction mixture was maintained constant. Then, the reaction was started through the addition of 30 µL of 10 mM of NADH (nicotine adenine dinucleotide). This reducing cofactor is essential for azoreductase activity. The decrease in absorbance (λ_{max} at 430 nm for Methyl Red and at ~600 nm for each azocompound were proportional to the rate of reduction of the different azocompounds.

a. Bacterial reductase assay in 50% EtOH with *azoRBC* or *i-azoRBC* enzyme

The reaction mixture includes 1 mL of absolute ethanol, 1 mL of 50 mM TRIS buffer of pH 7.0, 200 µL of free *azoRBC* or immobilized bacterial *i*-azo-RBC(1 µg/mL dissolved in 50 mM TRIS buffer of pH 8.0), and 200 µL of the corresponding azocompound (250 µg/mL in ethanol) at 25°C. The absorbance of the reaction mixture was registered to ensure that it was maintained constant and no precipitation nor undesirable adsorptions of the substrates on. The reaction was initiated through the addition of 30 µL of 10 mM of NADH (nicotine adenine dinucleotide). The decrease in absorbance (λ_{max} at 430 nm for Methyl Red and at ~600 nm for each azocompound) were proportional to the rate of reduction of the different azocompounds.

In the case of immobilize enzyme (*i-azoRBC*), after the total reduction of each substrate (final absorbance lower than 0.01), the reaction mixture was easily separated from the immobilized enzyme by using a micro-filter syringe. Since, azo-BODIPY compounds **5** were more soluble in the presence of 50% ethanol, and hence, higher substrate concentration could be used, the color changes associated with the reduction reaction products and the fluorescence of the different reaction products in 50% ethanol could be observed. The reaction mixture was placed in a fluorescent cell and illuminated with a deuterium lamp in the dark. The different azo-BODIPY were transformed into distinct fluorophores with pink, violent, or orange fluorescence. Representative examples of the products emitting a more intense fluorescence are shown in the following Table S5.

Table S5. Observation under a deuterium lamp of the reaction products of different azo-BODIPY after their enzymatic reduction with *i-azoRBC* in aqueous medium (with 50% ethanol).

**Released BODIPY**	**Fluorescence cuvettes**	λ_{em} (max nm)[a]
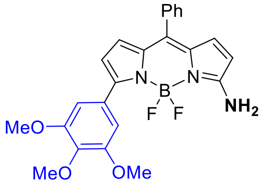 **6g**	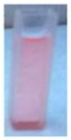	576
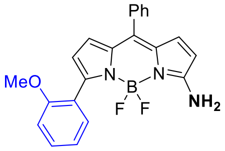 **6e**	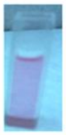	565
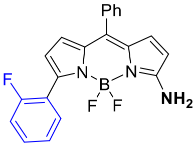 **6f**	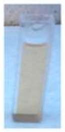	552

(a) From the emission spectra spectra measurements described in Table 3 of the manuscript.

The reaction temperature was 25°C. The absorbance of the reaction mixture was constant before adding NADH. The rate of reduction of the azo-BODIPY was measured by the decrease in absorbance at the maximun λ_{abs} of each azo-BODIPY per minute. The reduction of Methyl Red is considered as 100% and the reduction rates of the different azo compounds are relative to it, results are shown in Table S6.

Table S6. Enzymatic reduction of representative azo-BODIPY compounds **5**.

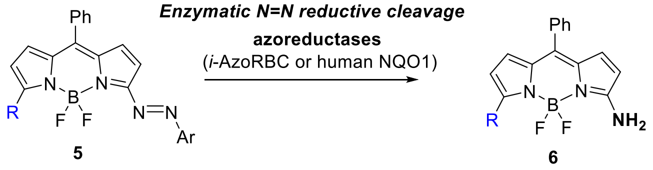

Entry	Azo compound	R	λ_{abs} (nm)[a]	*i-azoRBC*[b] (%)[c]	NQO1[b] (%)[c]
1	**Methyl red**	-	430	4 (100)	2 (100)
2	**5i**	2-thienyl	615	4 (100)	0.6 (30)
3	**4a**	Cl	608	3.2 (80)	1 (50)
4	**5c**	anthracenyl	610	5 (125)	3.4 (170)
5	**5e**	2-MeOPh	614	1.2 (30)	1 (50)
6	**5f**	2-F-Ph	616	4.4 (110)	0.5 (25)
7	**5g**	3,4,5-(OMe)$_3$Ph	630	2 (51)	1.4 (70)

[a] Concentration $2.5 \cdot 10^{-6}$ in EtOH/H_2O. [b] Activity = µmoles/min of reduced azocompound per mg of enzyme used. [c] Activity percentage considering the reduction of Methyl Red as 100%.

8.5 TIME-COURSE OF THE ENZYMATIC REDUCTION OF AZO-BODIPY 5i

The reduction process was carried out in the presence of the reducing cofactor, NADH, and monitored by the decrease in the absorbance at 615 nm for **5i**. The immobilized enzyme and the azocompound **5i** are incubated in 50% ethanol in 50 mM TRIS buffer at pH 7.0 in a spectrophotometric cell with stirring. Without the NADH, the absorbance at 615 nm (0.3) remains unaltered for 20 minutes, and after addition of reduced NADH the absorbance decreases down to 0.001 in 4 minutes (Figure S16). At this moment, the reaction mixture exhibits a pink fluorescence under a deuterium lamp. In the absence of enzyme, the addition of NADH to the azocompound does not modify the absorbance at 615 nm and the azocompound remains unmodified.

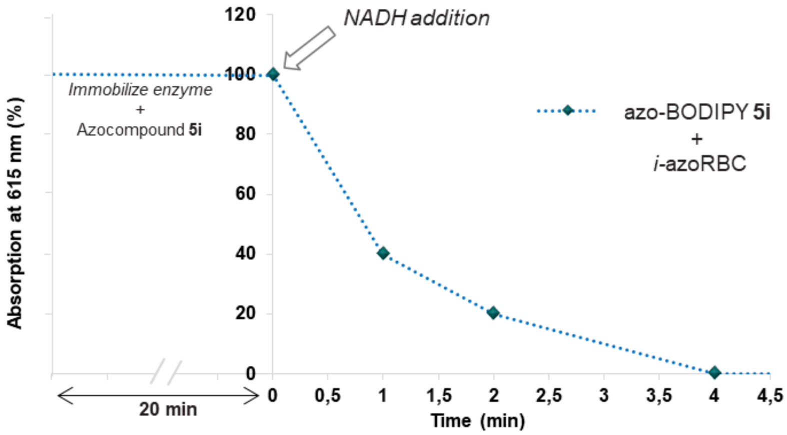

Figure S16. Time-course of the enzymatic reduction of azo-BODIPY **5i** with *i-azoRBC* upon addition of NADH (Absorbance at 615 nm, 100% corresponds to 0.3 a.u.).

9. CELL CULTURE STUDIES

9.1 Cell culture

HeLa (human cervical epithelial cell line) has been used as tumor cell model. Cell line was grown in Dulbecco's Modified Eagle's Medium (DMEM, Gibco, Paisley, Scot- land, UK) supplemented with fetal calf serum (FCS 10%, Gibco), and 0.5% of antibiotics (penicillin G [10,000 U/mL] and streptomycin sulfate [10,000 mg/mL] (Gibco)). DMEM supplemented with FCS and antibiotics will be referred to as complete medium. Cells were grown in a MIDI40 cell incubator (Thermo Scientific), with a 5% CO_2 atmosphere, a 95% relative humidity and a constant temperature of 37 ºC. For the photocytotoxicity experiments, cells were plated on 24 wells plates and for fluorescence experiments, cells were plated onto round coverslips placed into wells.

9.2 Administration of compounds

Stock solutions of the corresponding compounds (**5i** or **6i**) were prepared in DMSO (Panreac) at a concentration of 1 mg/mL (2 mM and 2.7 mM respectively). The work solutions were obtained by dissolving the compounds in complete medium. The final concentration of DMSO was always lower than 0.5% (v/v), and the lack of toxicity of this solvent for the cells was also tested and confirmed. All the treatments were performed when cultures reached around 60-70% of confluence.

For the time-course analyses of oxygen deprived conditions, HeLa cell grown on coverslips were treated with **5i** (10 μM) for 2 h, place on a glass slide and visualized under fluorescent microscopy after 0, 10, 20 and 40 min.

To analyze the impact of azoreductase inhibitor, HeLa cells were incubated with DPI (10 μM in complete medium) and the azo **5i** for a period of 2 h followed by 40 min of oxygen deprivation.

To carry out controls with HeLa cells incubated with **5i** (10 μM) under normoxia conditions, they were cultured on 35mm petri dish with a 10mm coverslip inserted into the bottom (MatTek). The cells were analyzed under a confocal microscopy.

9.3 Intracellular localization.

HeLa cells were seeded on coverslips placed into 24-well plates at densities of 3 x 10^4 cells and allowed to grow for 48 h. After that, cells were incubated for 2 h in the presence of the corresponding azo-BODIPY **5i** at 10 µM. The samples were washed twice with PBS, then mounted on slides with PBS and examined with a fluorescence microscope.

9.4 MTT viability assay

Cell viability were documented by the MTT assay.[26] Following appropriate treatments, 3-(4,5- dimethylthiazol-2-yl)-2,5-diphenyltetrazolium bromide (MTT) solution was added to each well at a concentration of 0.5 ng/mL, and plates were incubated at 37 ºC for 2-3 h. The resulting formazan crystals were dissolved by the addition of DMSO and absorbance was measured at 540 nm. The results were expressed as cell survival percentage of control (cell survival (%) = (mean OD treated cells/mean OD value of control cells) x 100%).

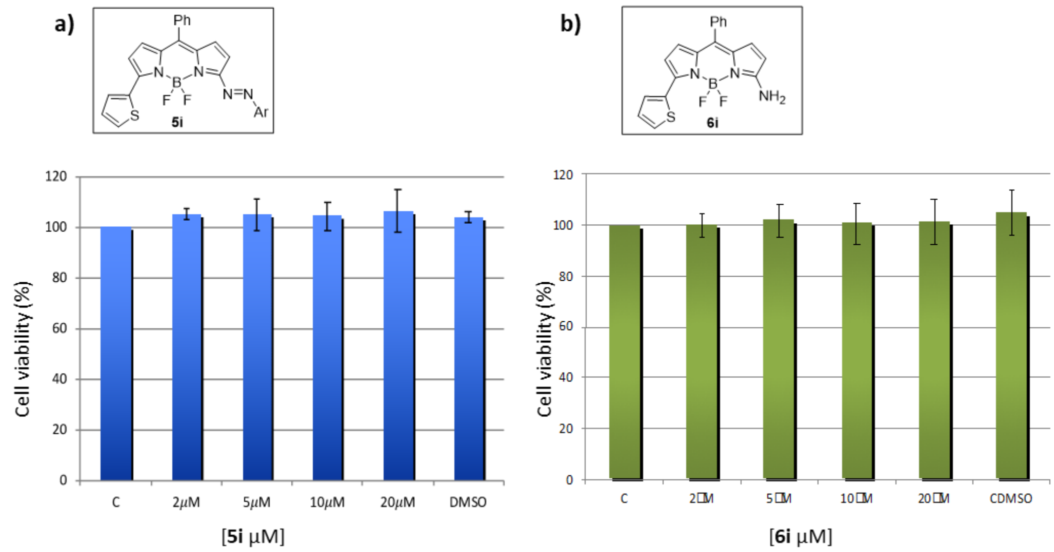

Figure S17 Quantification of HeLa cell viability 24h after treatment during 2 h in the dark with different concentrations (2, 5, 10 and 20 µM) of **5i** (blue bars) and **6i** (green bars), evaluated using the MTT assay. The survival rates showed no significant toxicity at concentrations up to 20 µM**.**

9.5 Fluorescence Microscopy

Epifluorescence microscope Olympus BX61:

Microscopic observations and photographs were performed in an Olympus photomicroscope IMT-2, equipped with a HBO 100 W mercury lamp and the corresponding filter sets for fluorescence microscopy: UV (365 nm), blue (450-490 nm, exciting filter BP 490) and green (545 nm, exciting filter BP 545) and a 100 x oil objective lens.

The cell images were taken with a digital Camera: Olympus DP70; Fluorescence Filter Cube: U-MWIG; Exciter Filter: BP520-550 nm; Dichroic Beam Splitter: DM565 nm; Barrier Filter: BA580-IF.

Confocal Laser Scanning Microscope coupled to an AxioObserver (Zeiss) inverted microscope LSM710:

Confocal Images were obtained in a LSM710 Confocal Laser Scanning Microscope coupled to an AxioObserver inverted microscope (Zeiss).

Cells were illuminated with a DPSS 561 nm laser. 63 x oil objective lens. Emission: 571-700 nm, Excitation: 561nm; dichroic filter: MBS 488/561

Quantification of the fluorescence of the images

To carry out the quantification of the fluorescence of the images, the following steps have been carried out with FIJI (version: 2.3.0/1.53f):

1. Calibration of the images to obtain the area in SI units (microns).
2. Isolation of the channel of interest (in our case red) (the original image is in RGB).
3. Subtract a common background value (specifically 3.5) obtained from the average of several regions of each times.
4. Selection of a specific signal by applying a threshold following the "Moments" algorithm since it adjusts correctly to the desired signal.
5. Measure of the specific signal using this algorithm.

The results represented in Figure S18 A corresponds to fluorescence images of HeLa cells (Figure S18 B) incubated with **5i** (+ AZO) and subjected to different oxygen deprivation times (0, 10, 20, 30, 40, 50 and 60 min). Each bar in Figure S18 (A) corresponds to the mean fluorescence intensity of three different fields from a single experiment. As can be seen in Figure S18 (A) the change in fluorescence increased 8.3 times from 0 min to 60 min of anoxia.

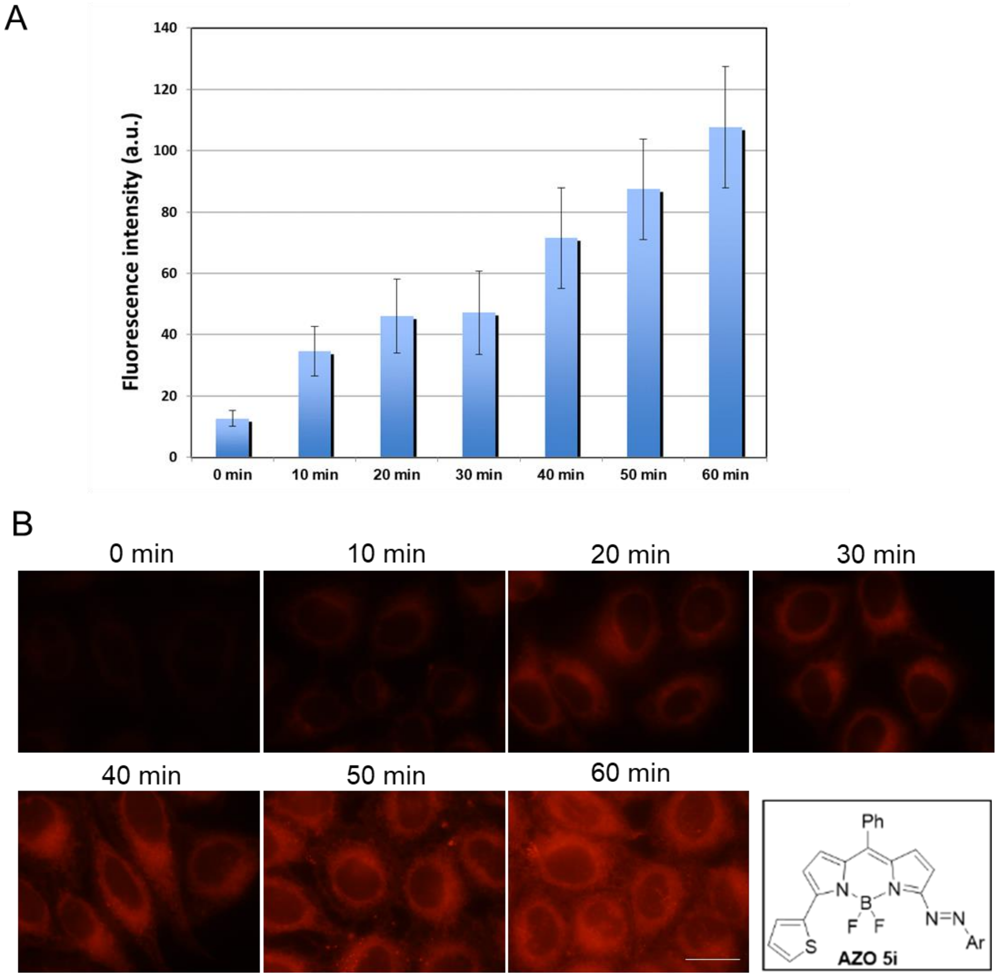

Figure S18 (A) Relative fluorescence intensity (calculated with Fiji version: 2.3.0/1.53f, data are mean fluorescence intensity +/- S.D.) of (B) fluorescence images of HeLa cells incubated with **5i** (+ AZO) and subjected to different oxygen deprivation times (0, 10, 20, 30, 40, 50 and 60 min). Scale bar: 20 μm. The excitation and emission wavelengths were 520-550 nm and 580 nm, respectively.

10. NMR SPECTRA OF NEW COMPOUNDS

^{1}H-NMR (300 MHz, $CDCl_3$)

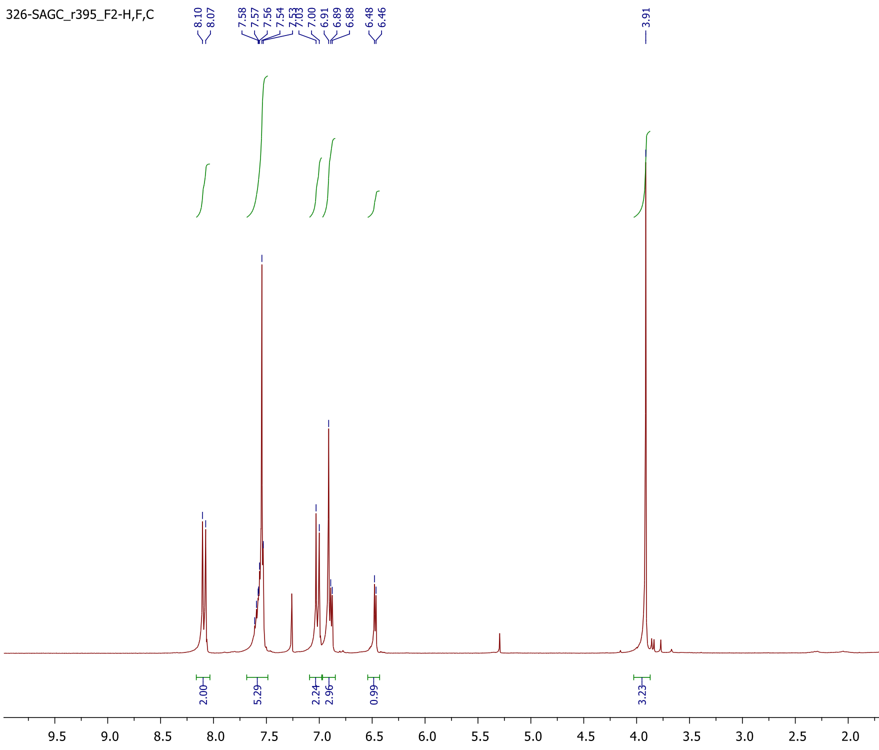

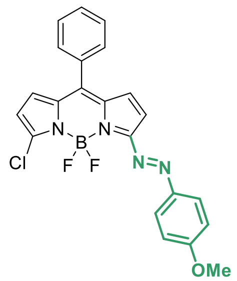

4a

^{13}C-NMR (75 MHz, $CDCl_3$)

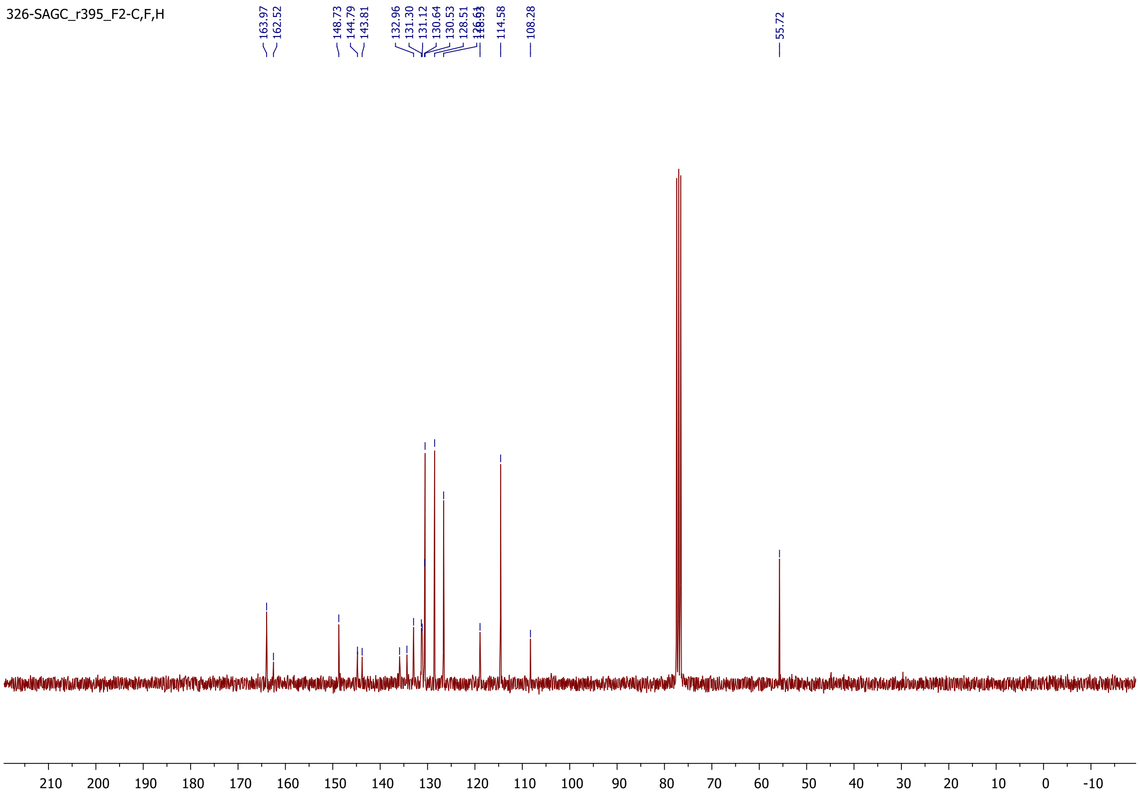

^{1}H-NMR (300 MHz, $CDCl_3$)

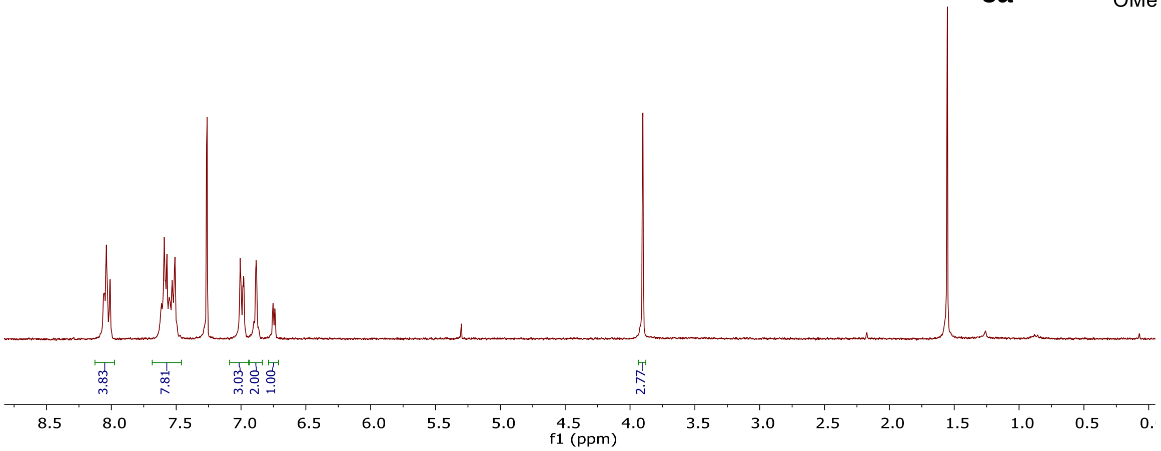

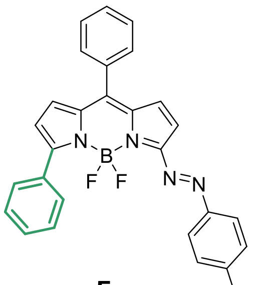

5a

^{13}C-NMR (75 MHz, $CDCl_3$)

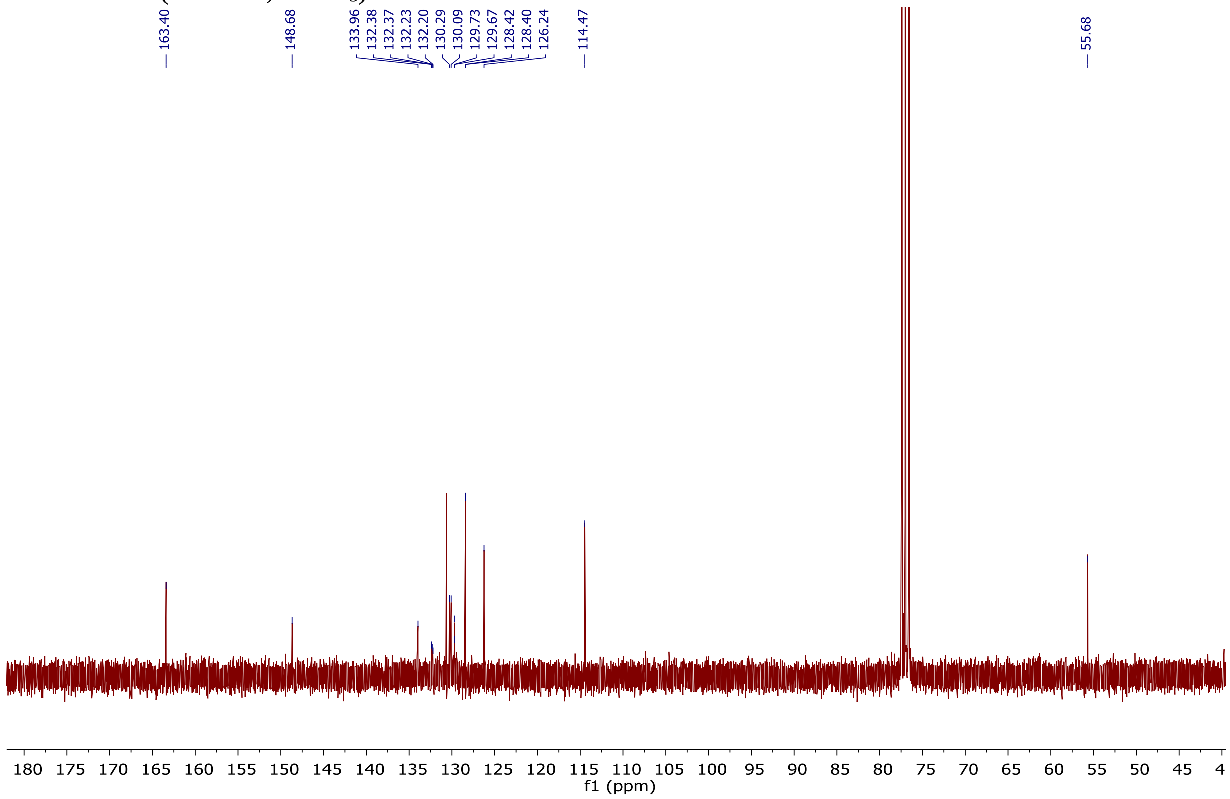

^{1}H-NMR (300 MHz, $CDCl_3$) **5b**

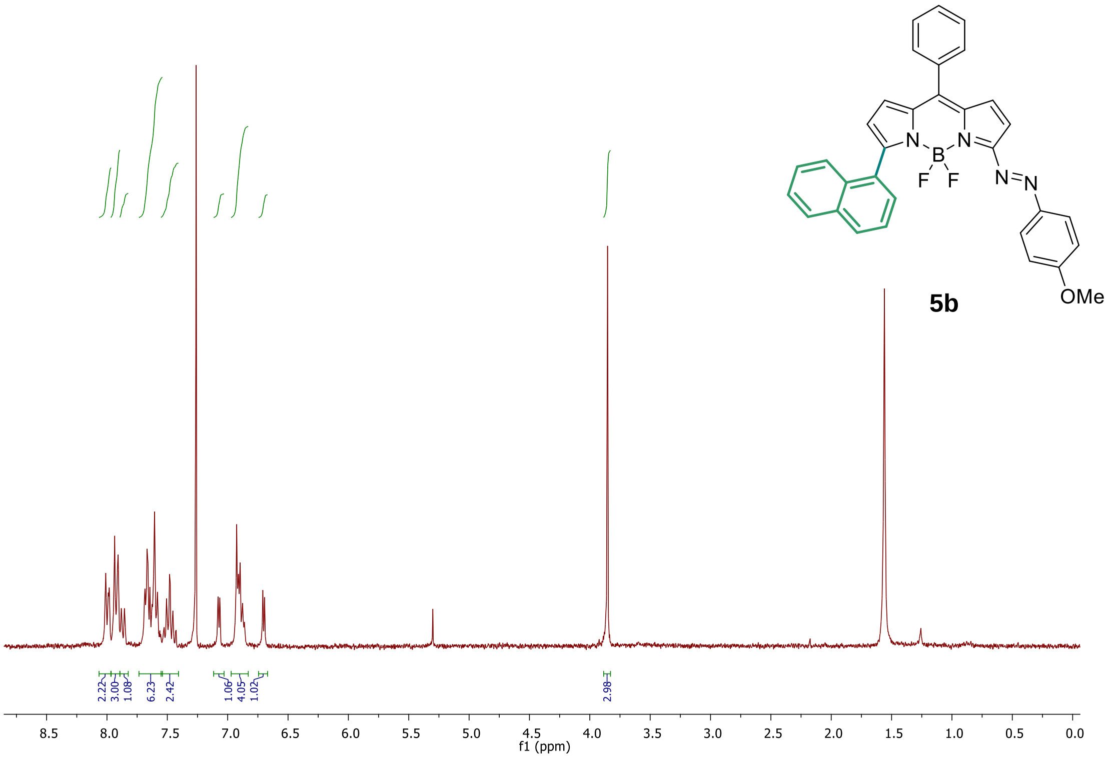

^{13}C-NMR (75 MHz, $CDCl_3$)

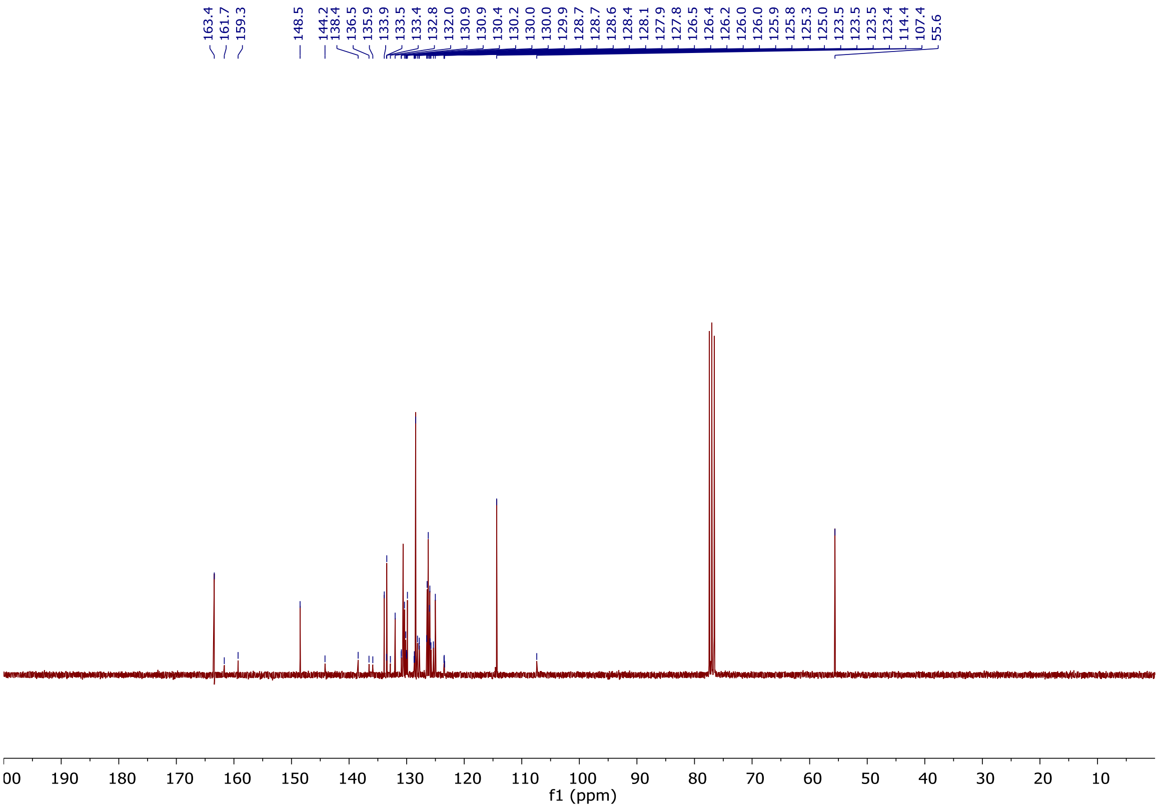

¹H-NMR (300 MHz, $CDCl_3$) **5c**

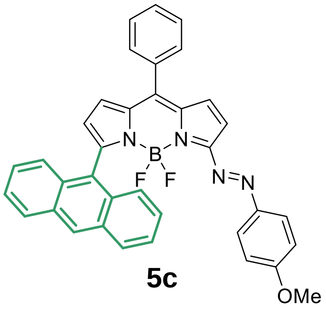

5c

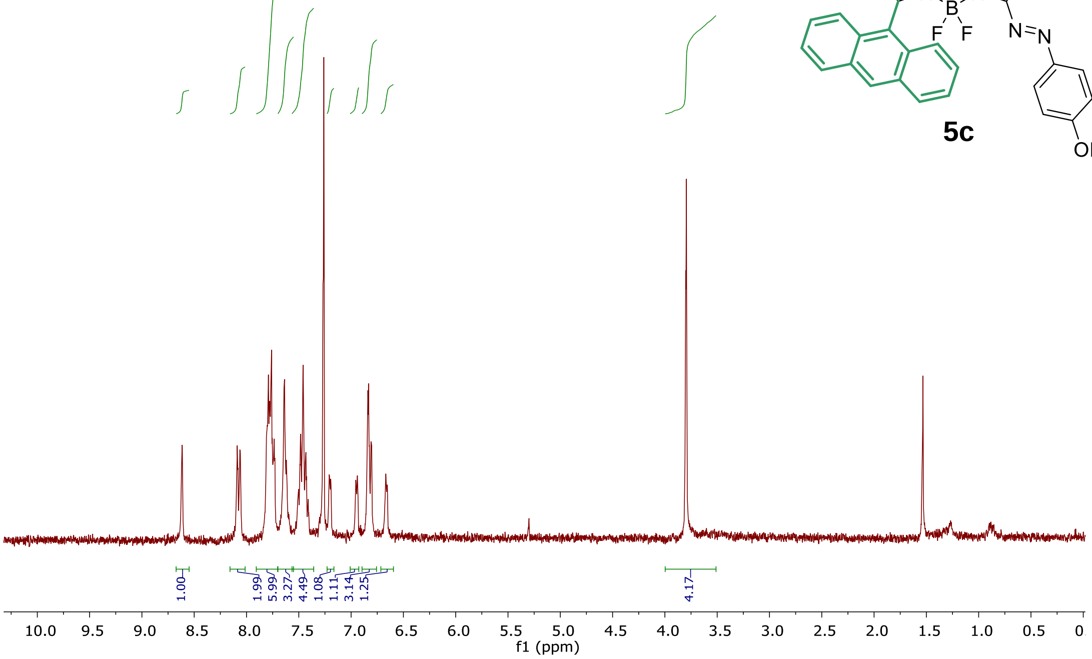

¹³C-NMR (75 MHz, $CDCl_3$)

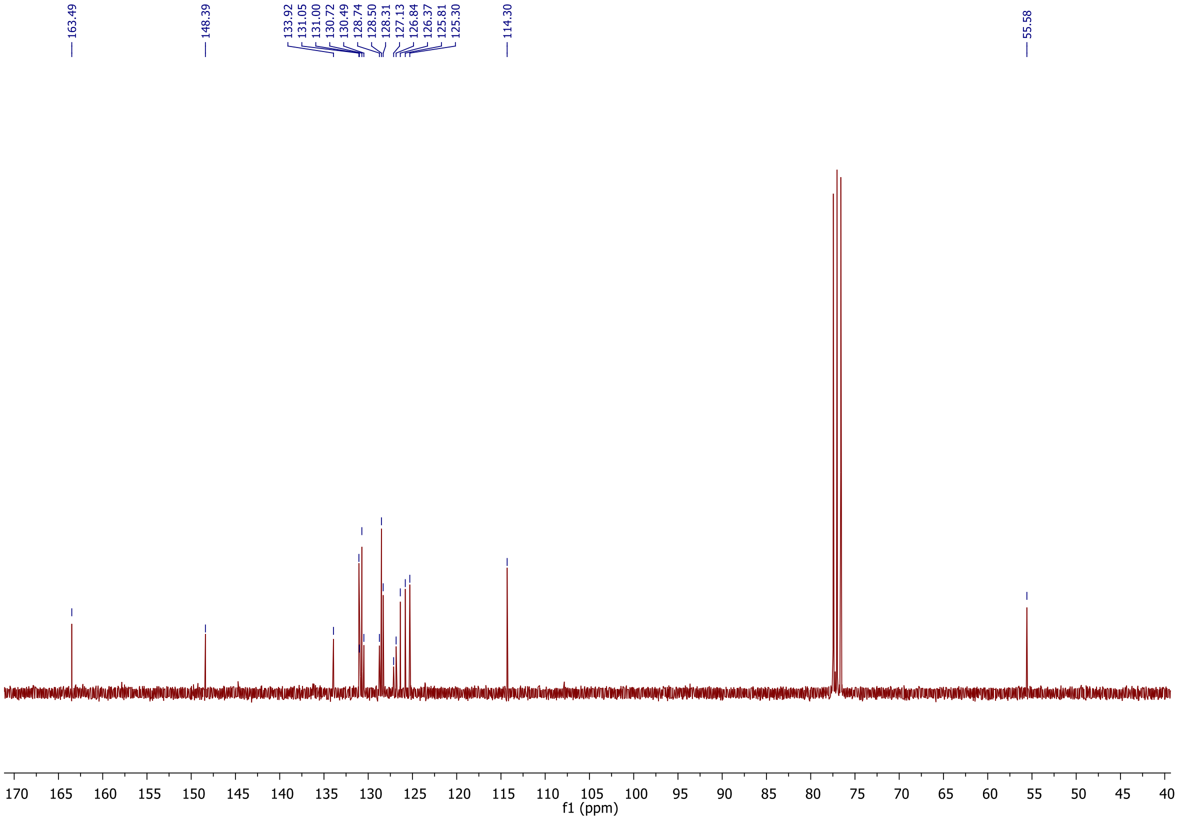

^{1}H-NMR (300 MHz, $CDCl_3$) **5d**

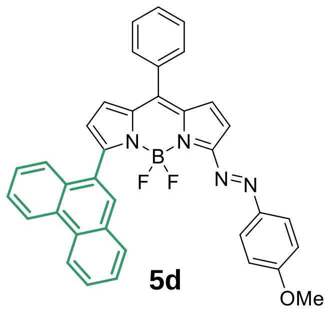

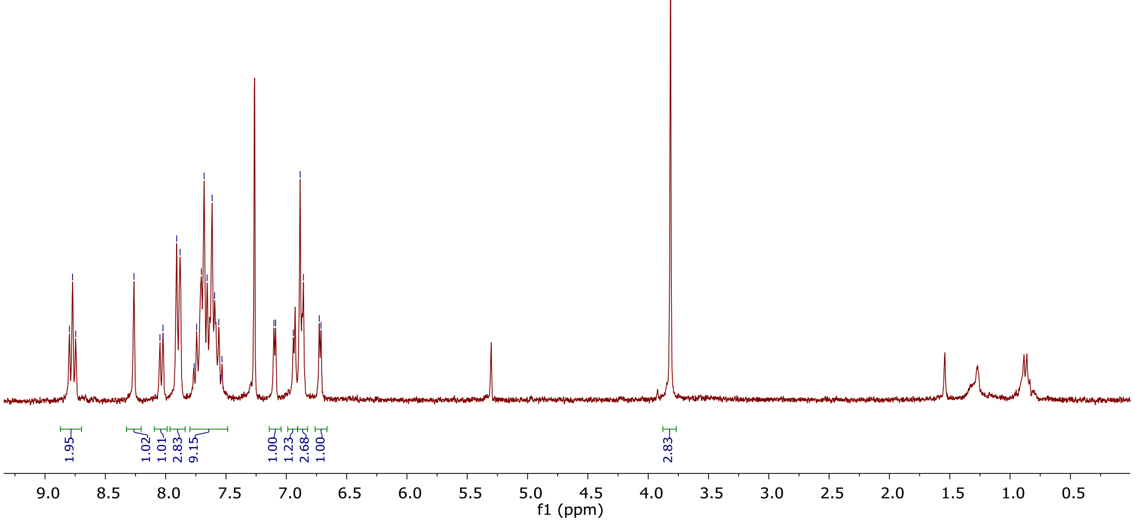

^{13}C-NMR (75 MHz, $CDCl_3$)

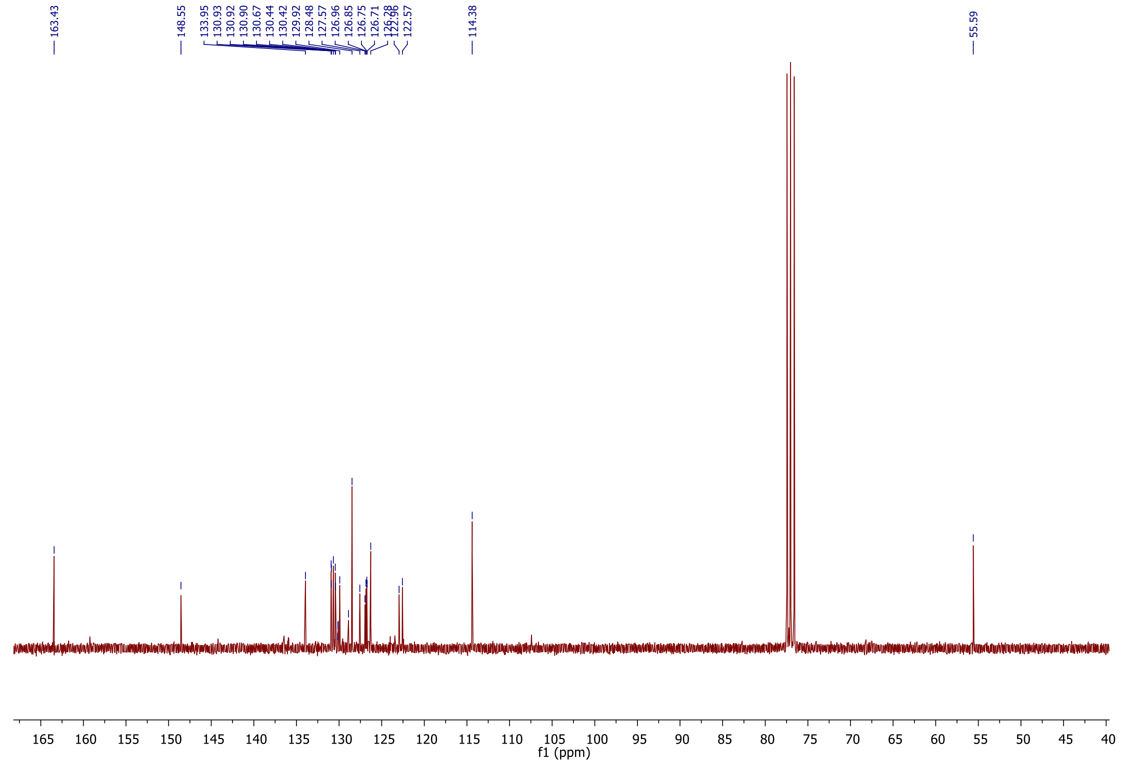

^{1}H-NMR (300 MHz, Acetone-d6) **5e**

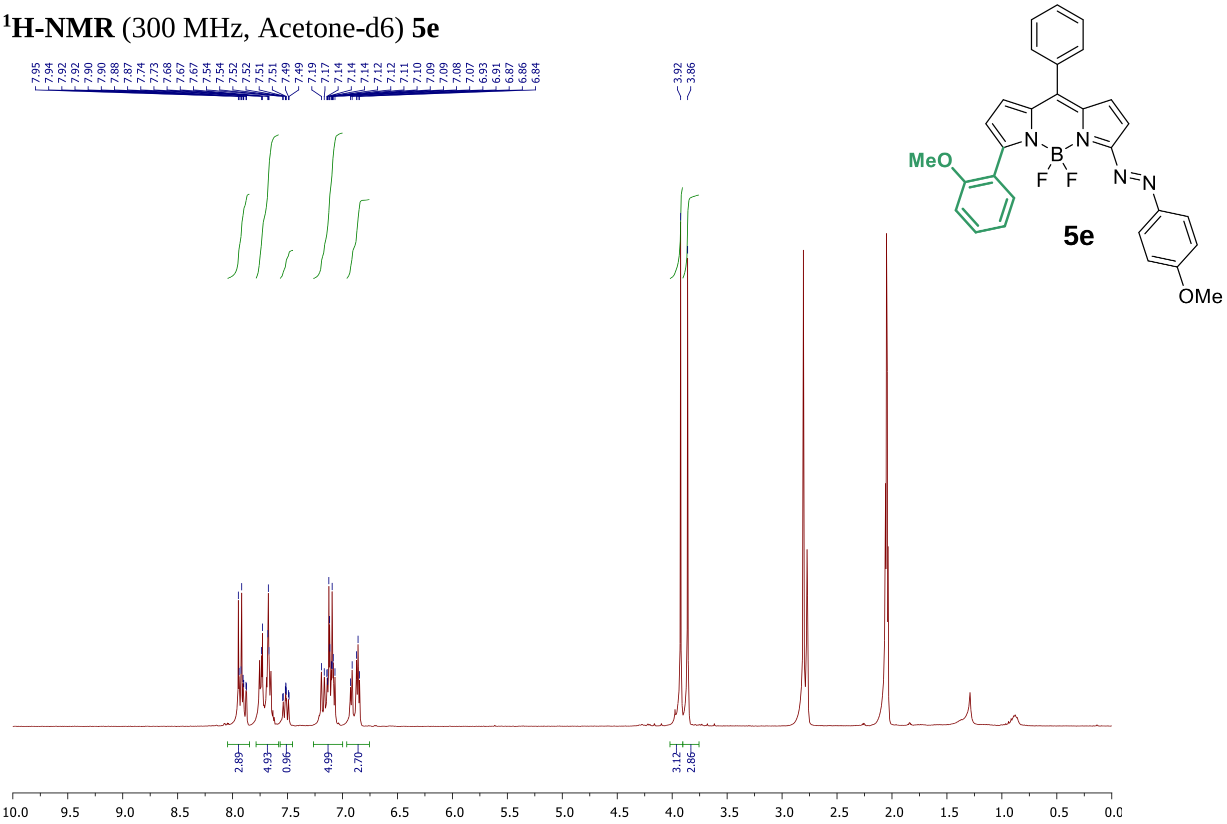

^{13}C-NMR (75 MHz, acetone-d6)

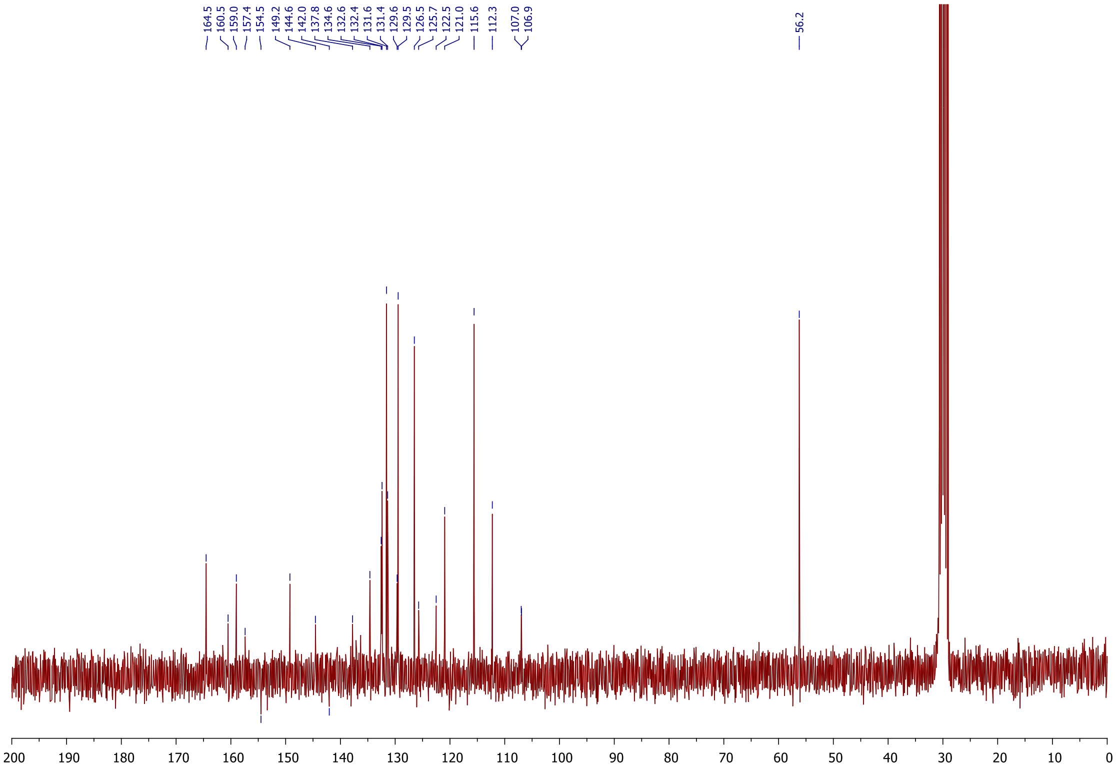

¹H-NMR (300 MHz, $CDCl_3$) **5f**

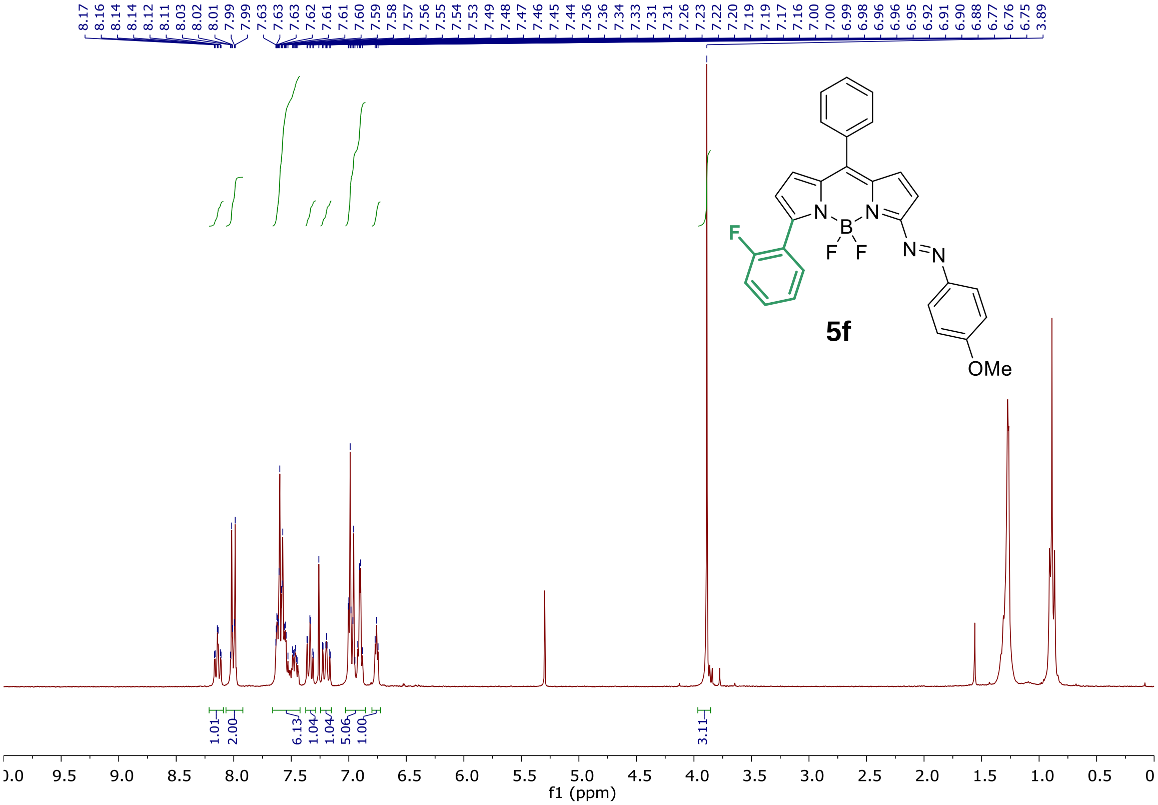

¹³C-NMR (75 MHz, $CDCl_3$)

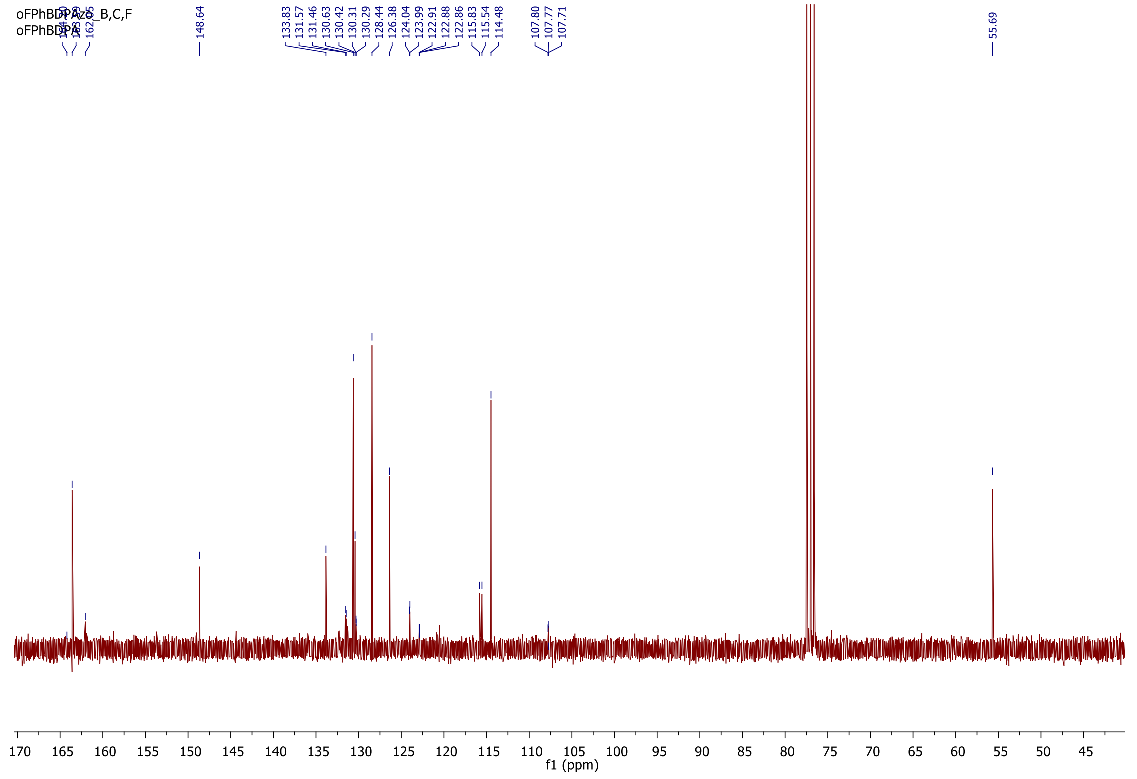

^{1}H-NMR (300 MHz, $CDCl_3$) **5g**

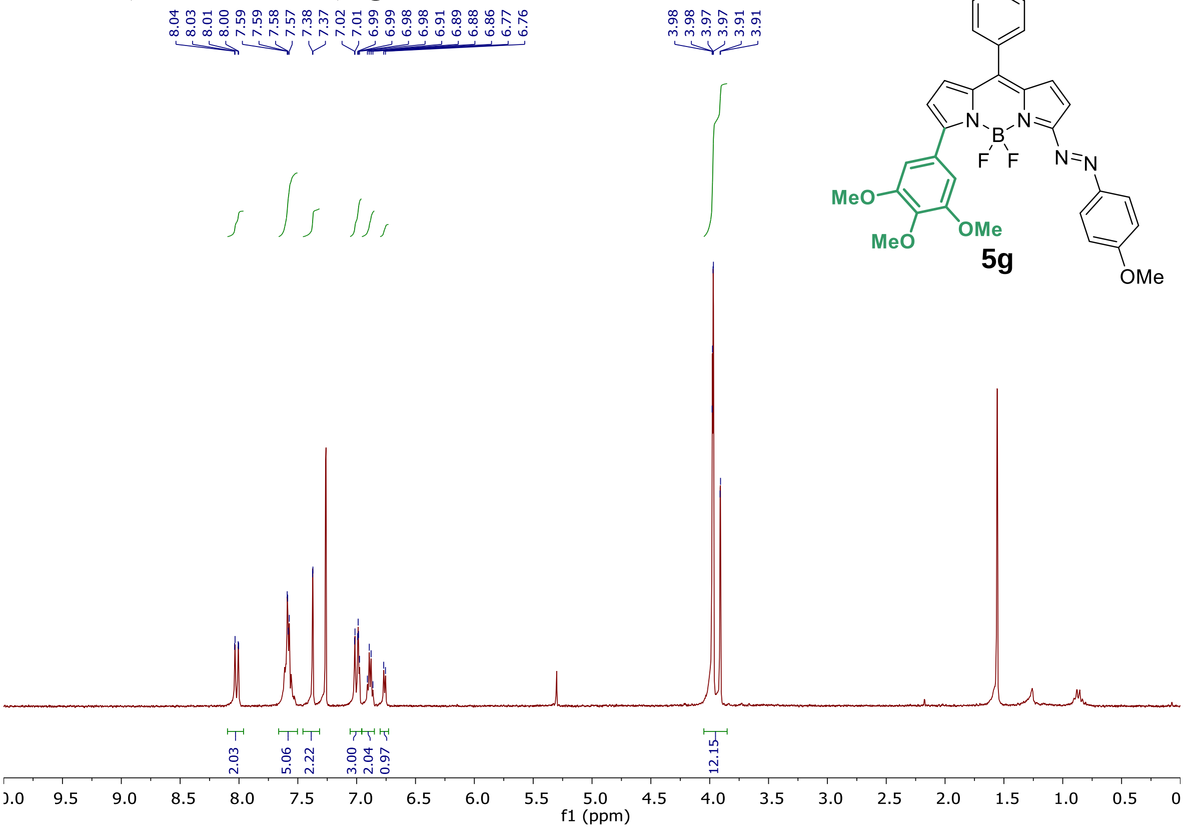

^{13}C-NMR (75 MHz, $CDCl_3$)

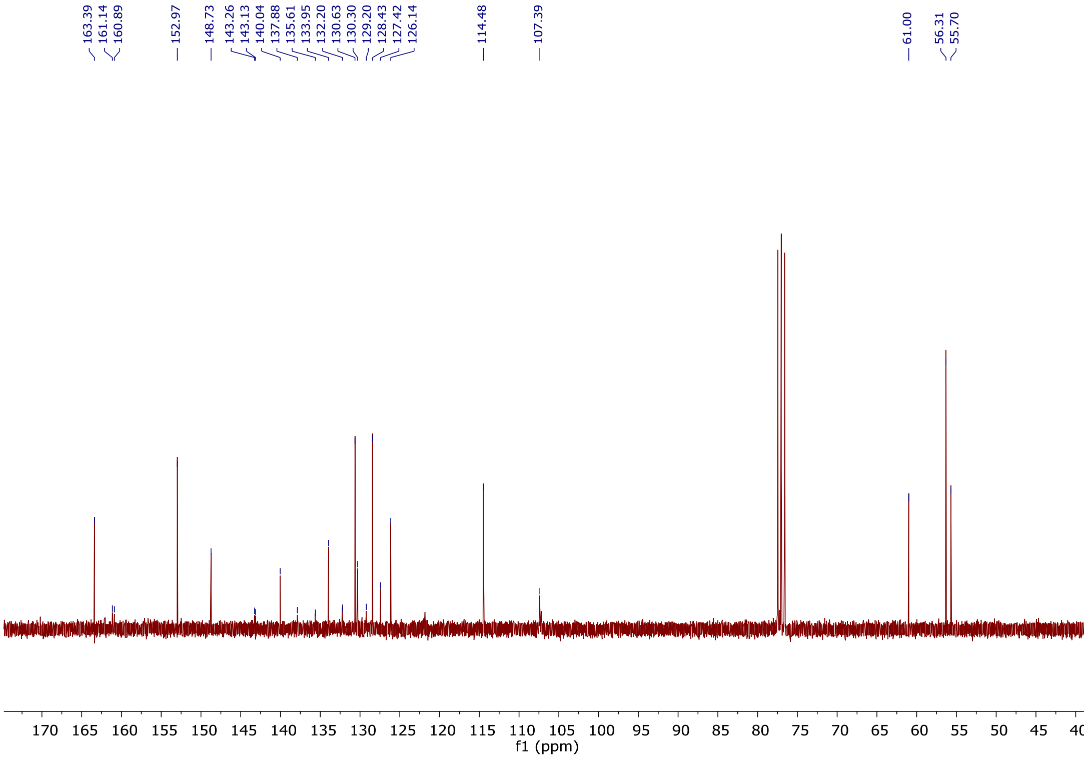

^{1}H-NMR (300 MHz, $CDCl_3$) **5h**

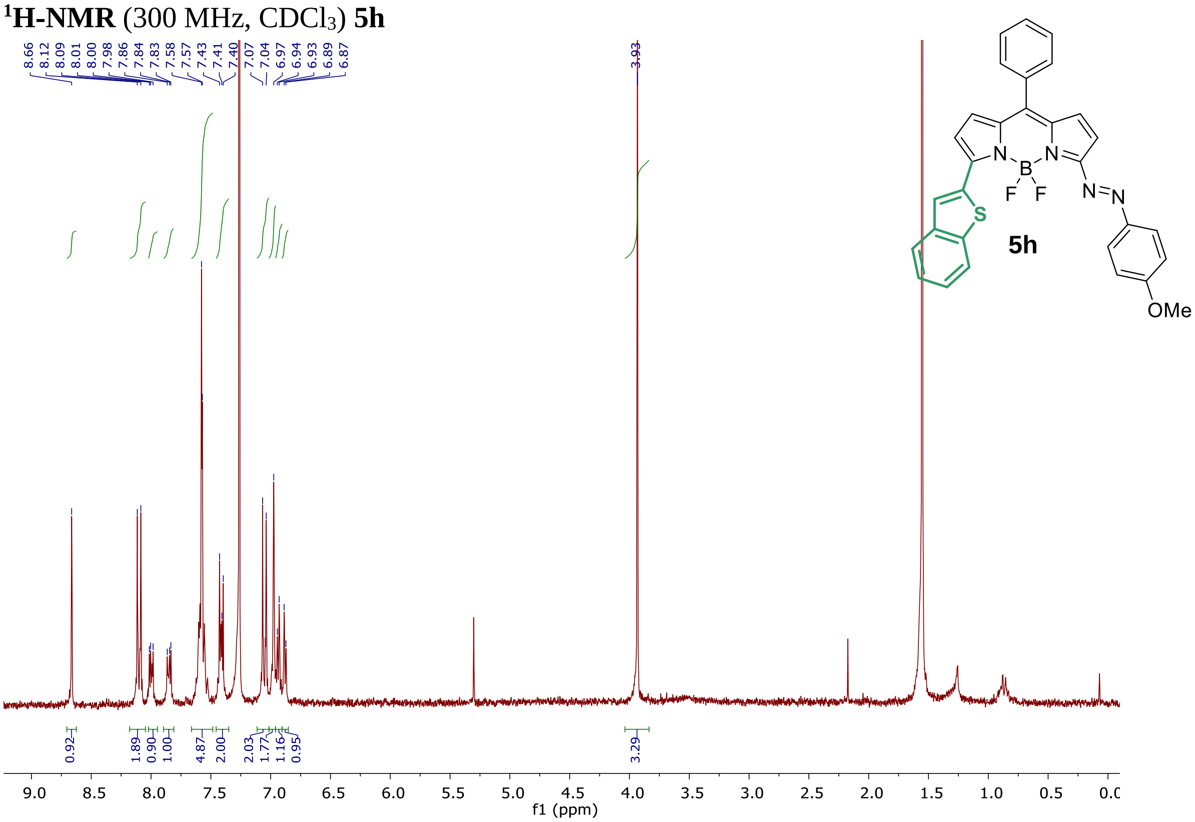

5h

^{13}C-NMR (75 MHz, $CDCl_3$)

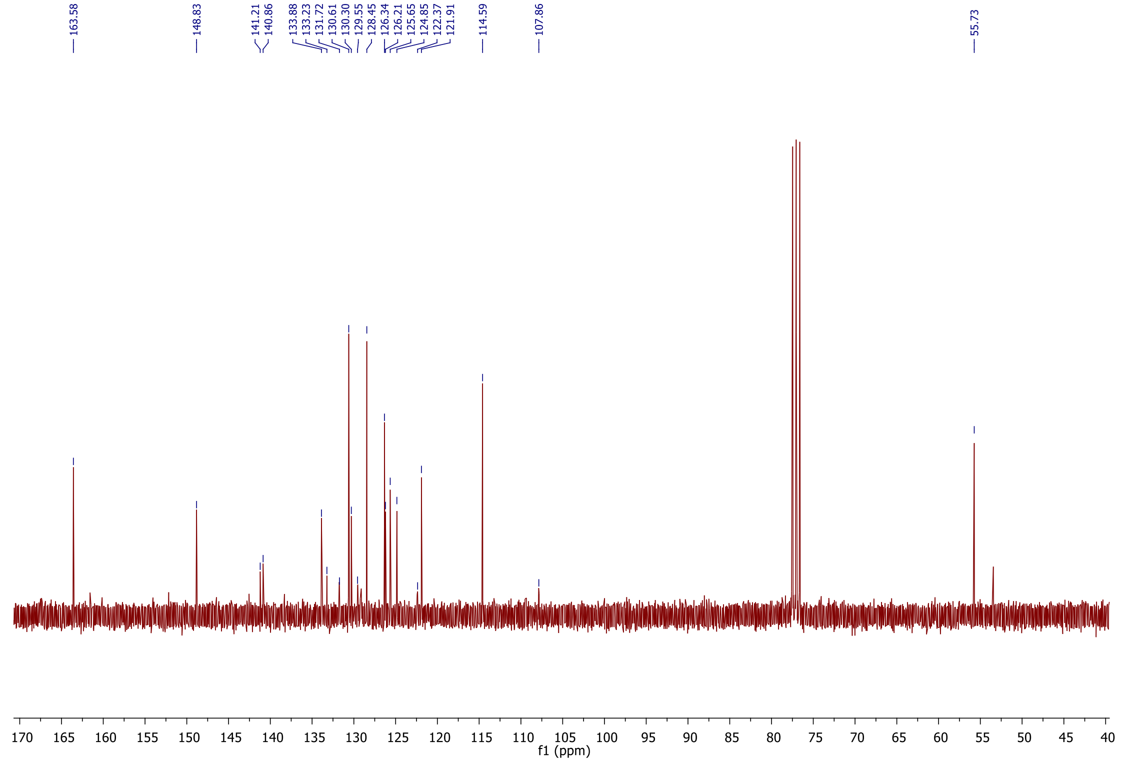

^{1}H-NMR (300 MHz, $CDCl_3$) **5i**

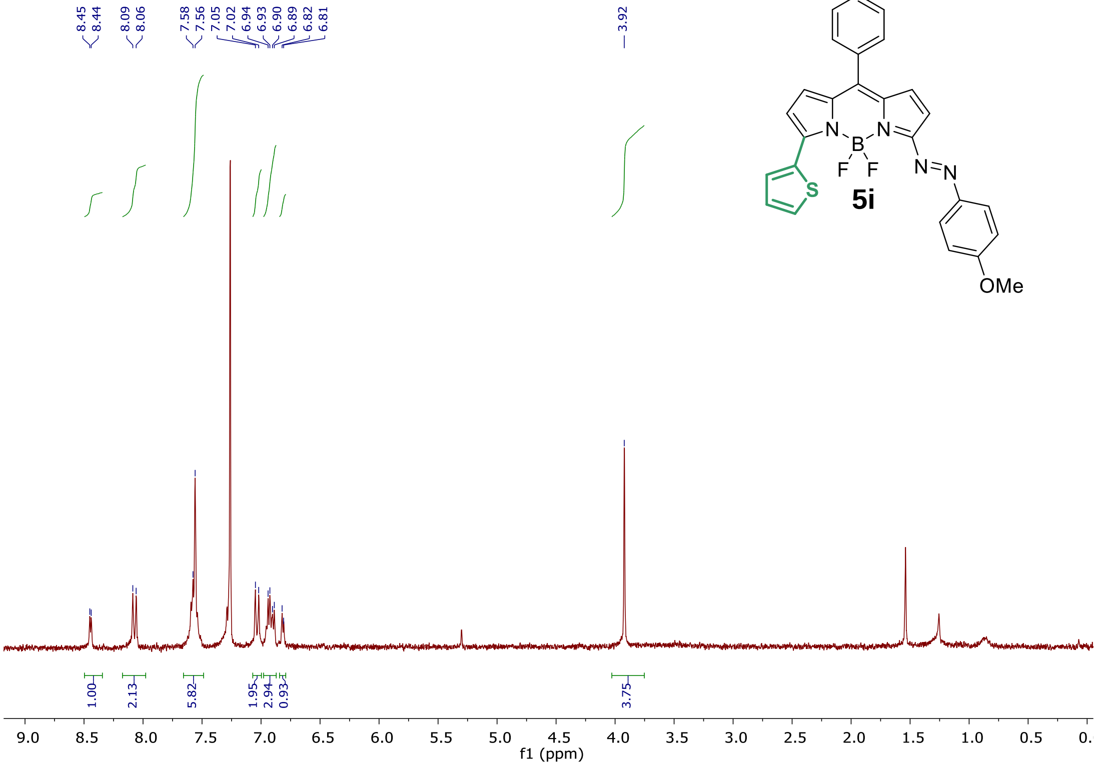

^{13}C-NMR (75 MHz, $CDCl_3$)

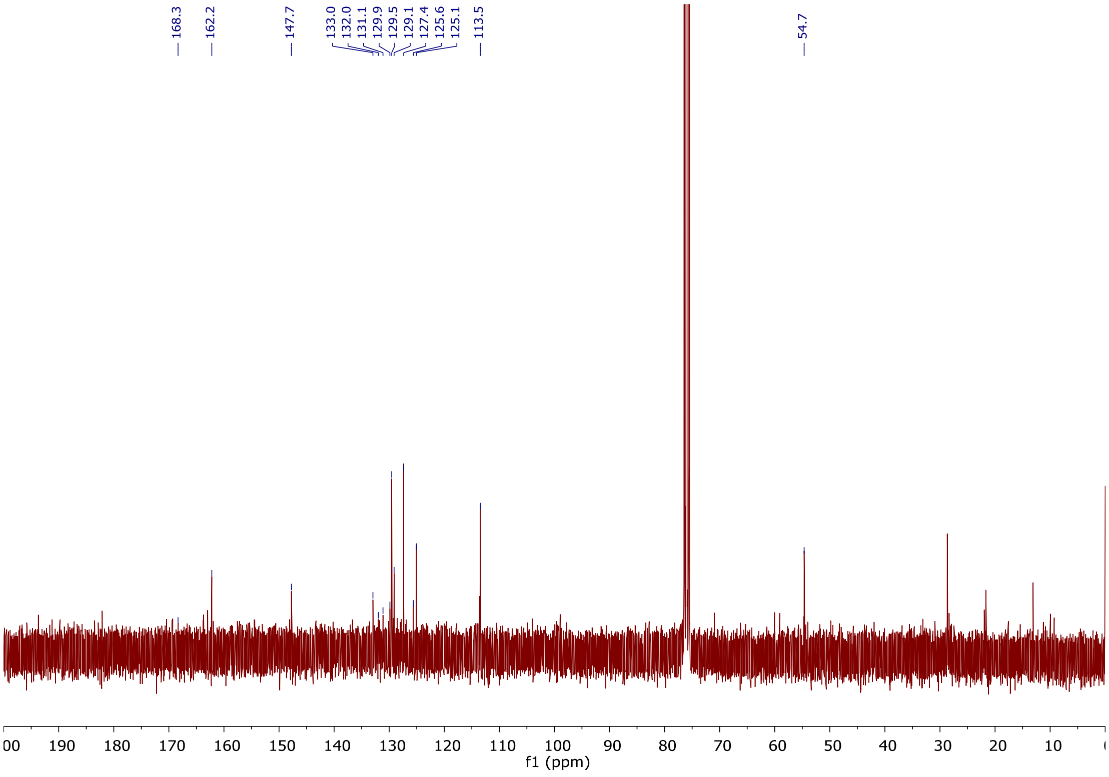

[1]**H-NMR** (300 MHz, $CDCl_3$) **6a**

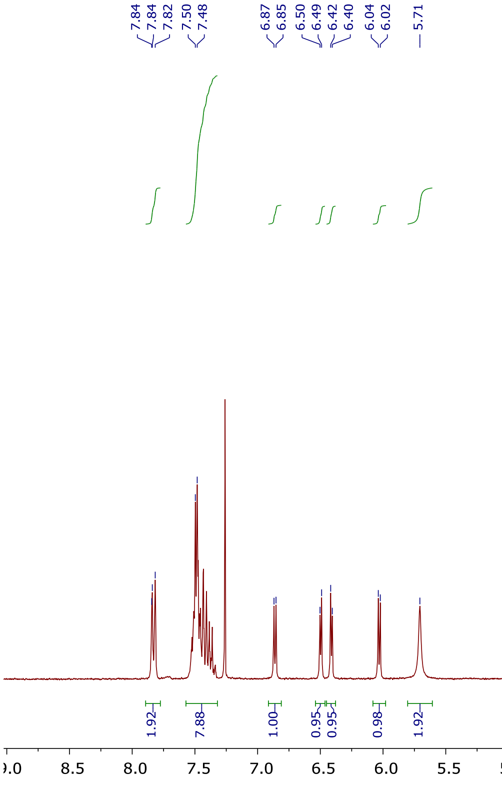

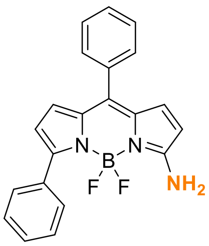

[13]**C-NMR** (75 MHz, $CDCl_3$)

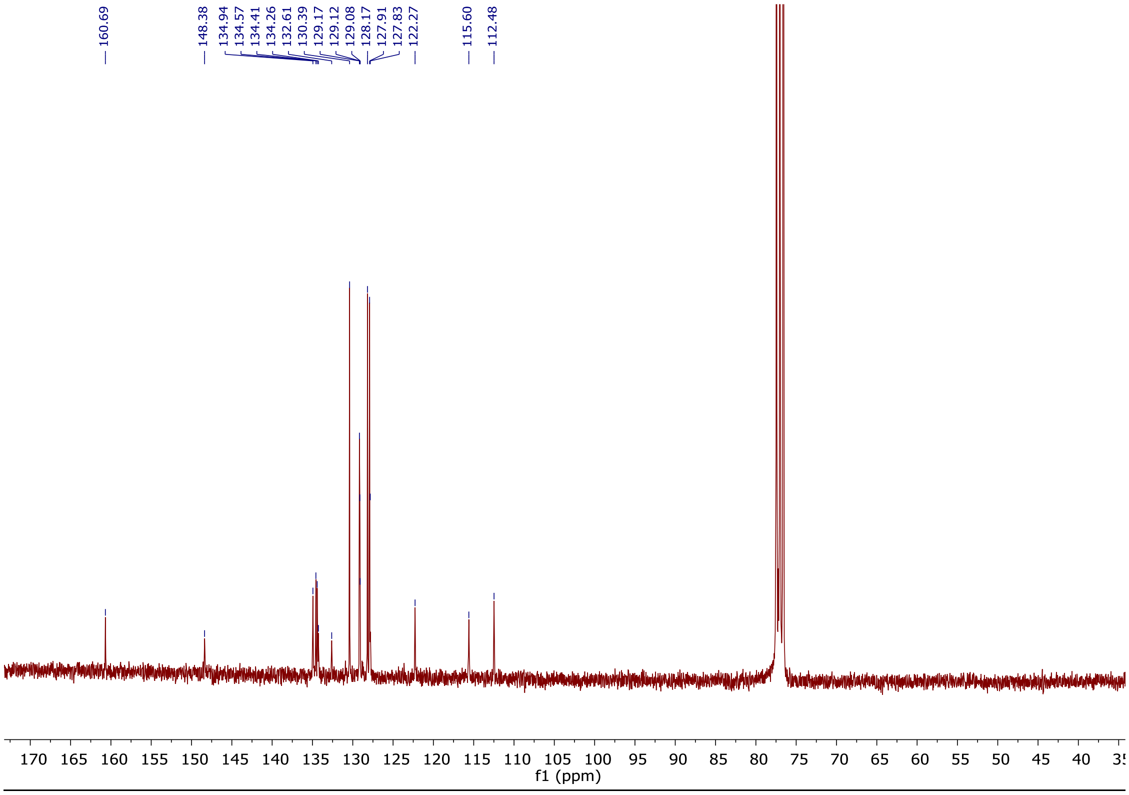

^{1}H-NMR (300 MHz, $CDCl_3$) **6b**

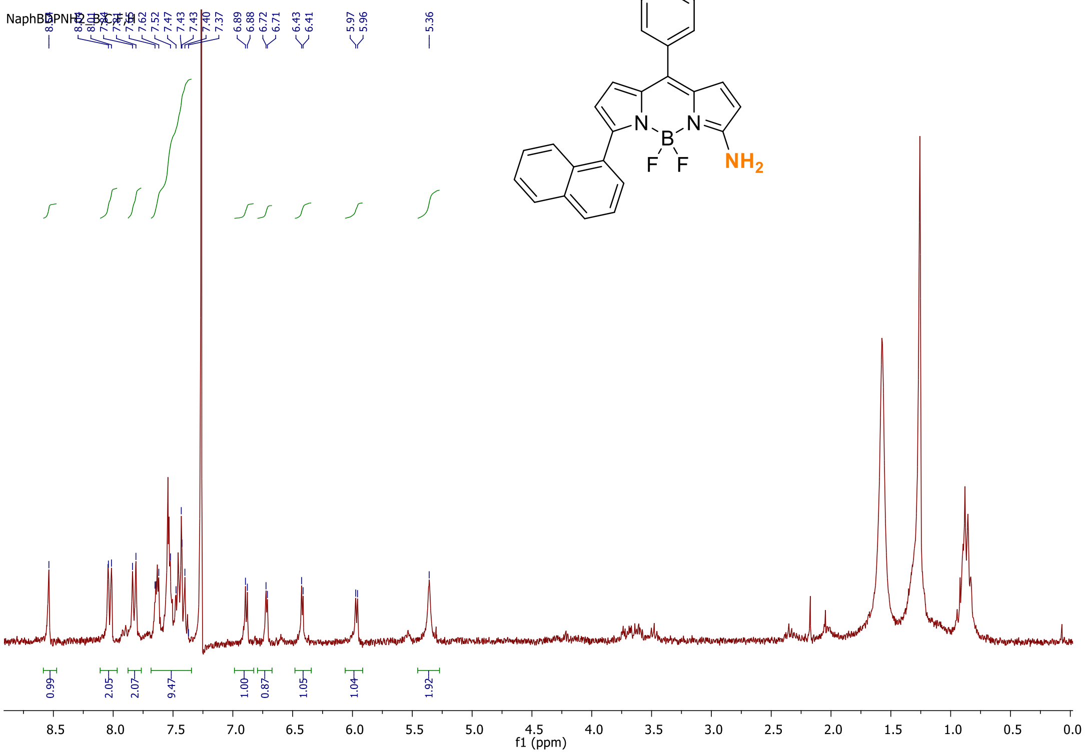

^{13}C-NMR (75 MHz, $CDCl_3$)

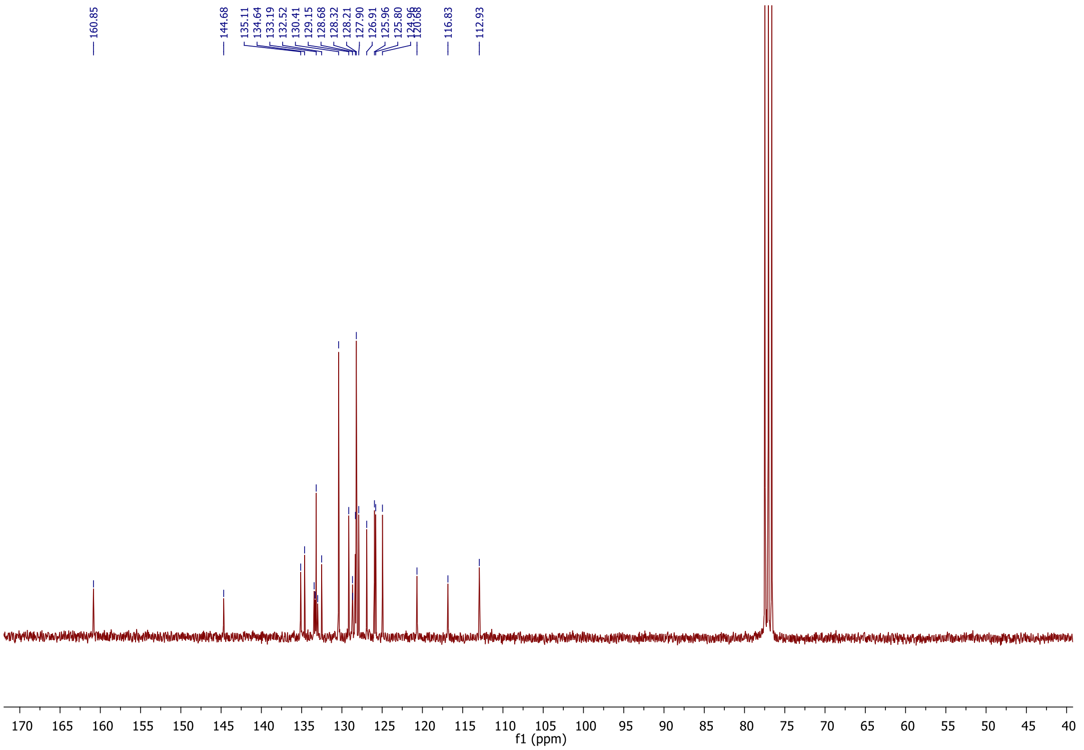

^{1}H-NMR (300 MHz, $CDCl_3$) **6c**

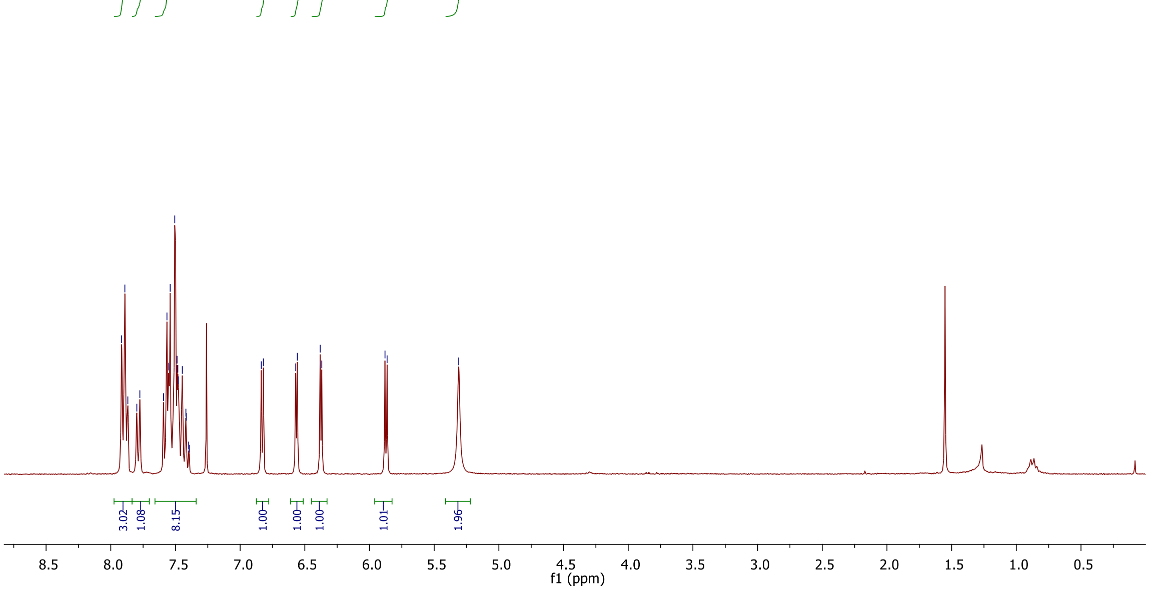

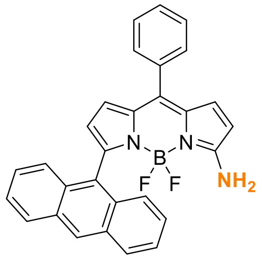

^{13}C-NMR (75 MHz, $CDCl_3$)

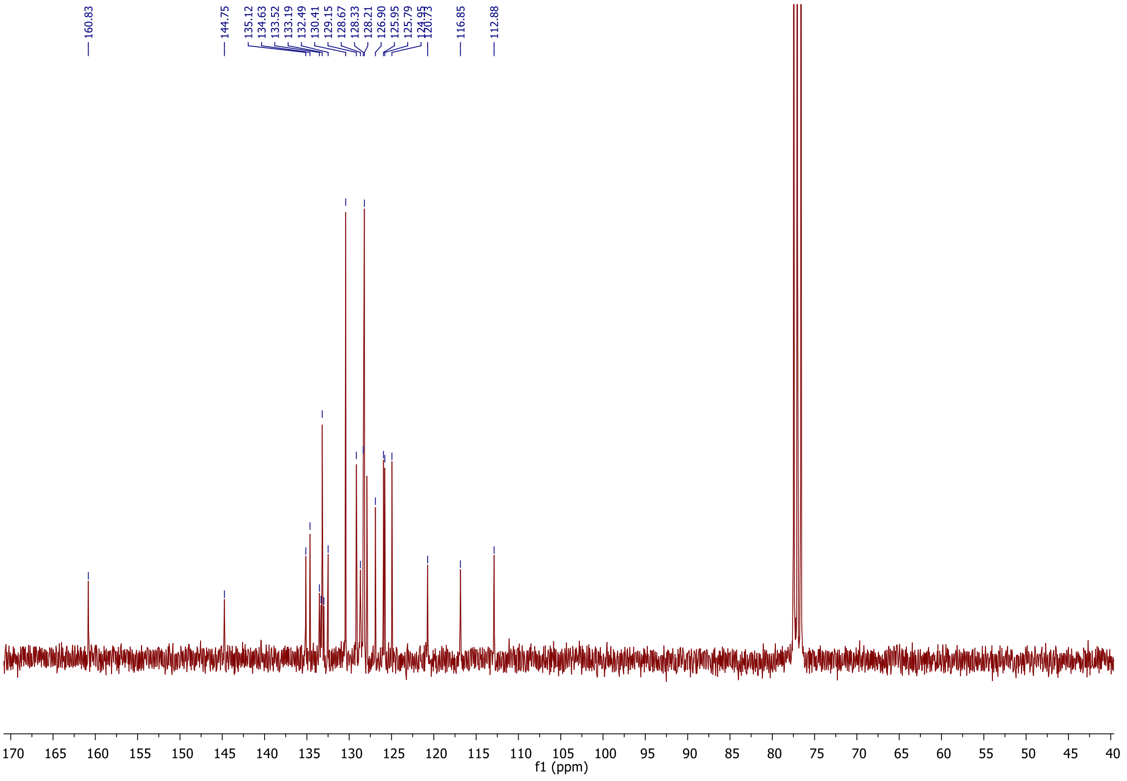

^{1}H-NMR (300 MHz, CDCl₃) **6d**

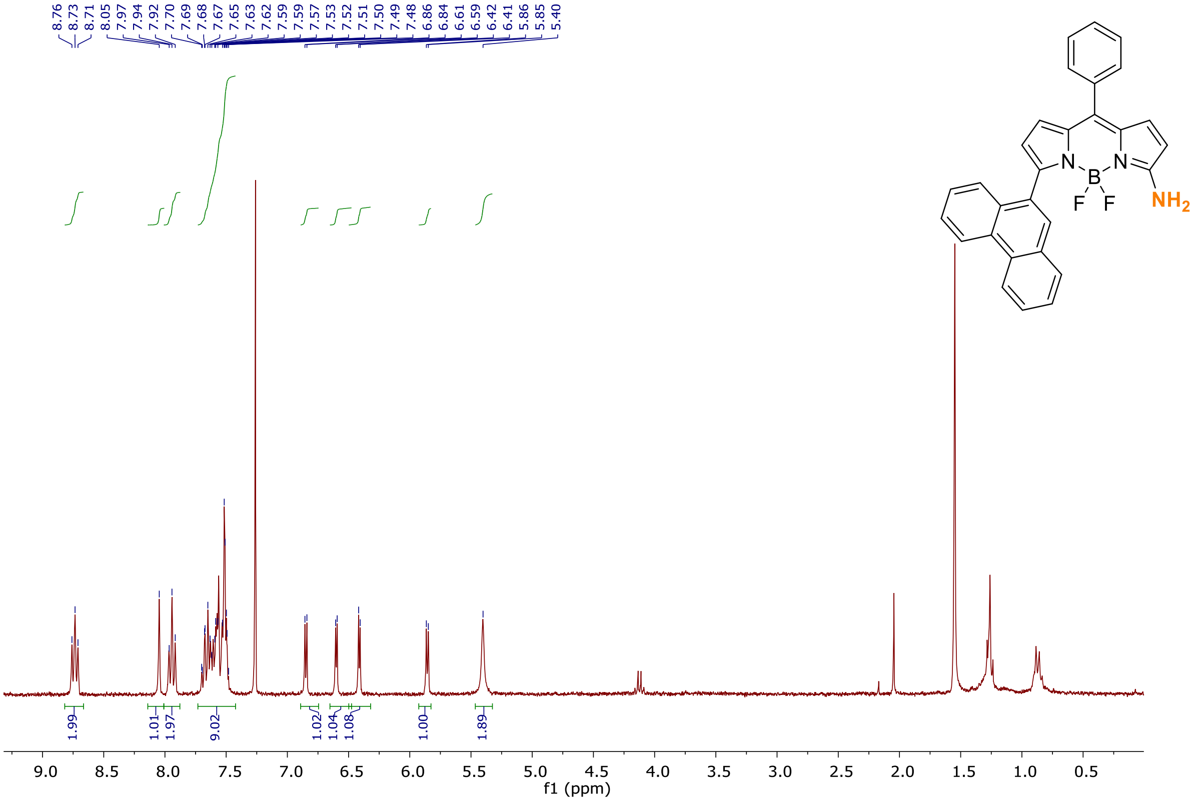

^{13}C-NMR (75 MHz, CDCl₃)

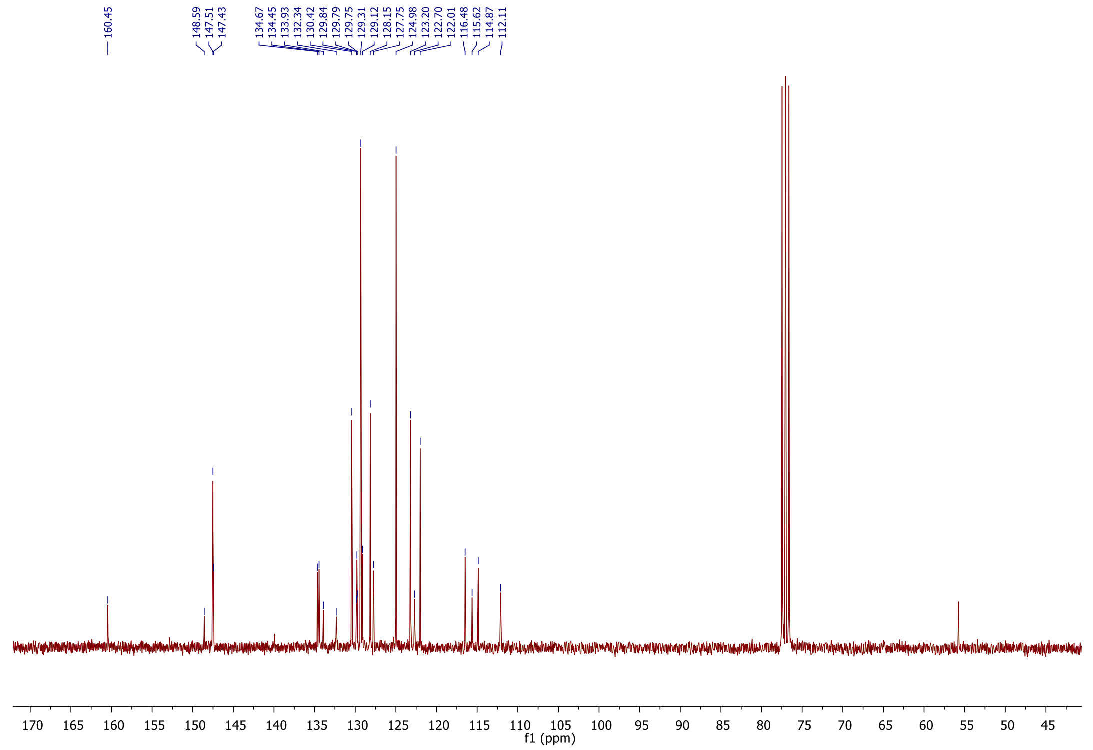

^{1}H-NMR (300 MHz, $CDCl_3$) **6e**

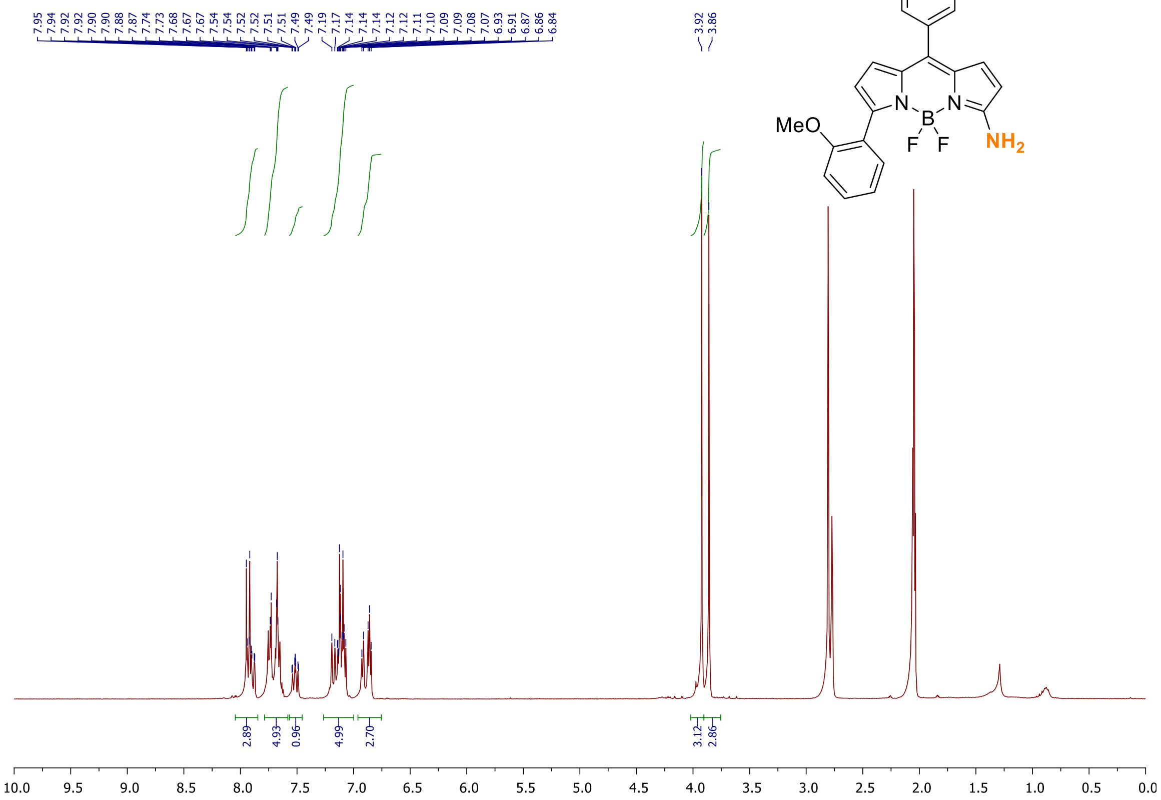

^{13}C-NMR (75 MHz, $CDCl_3$)

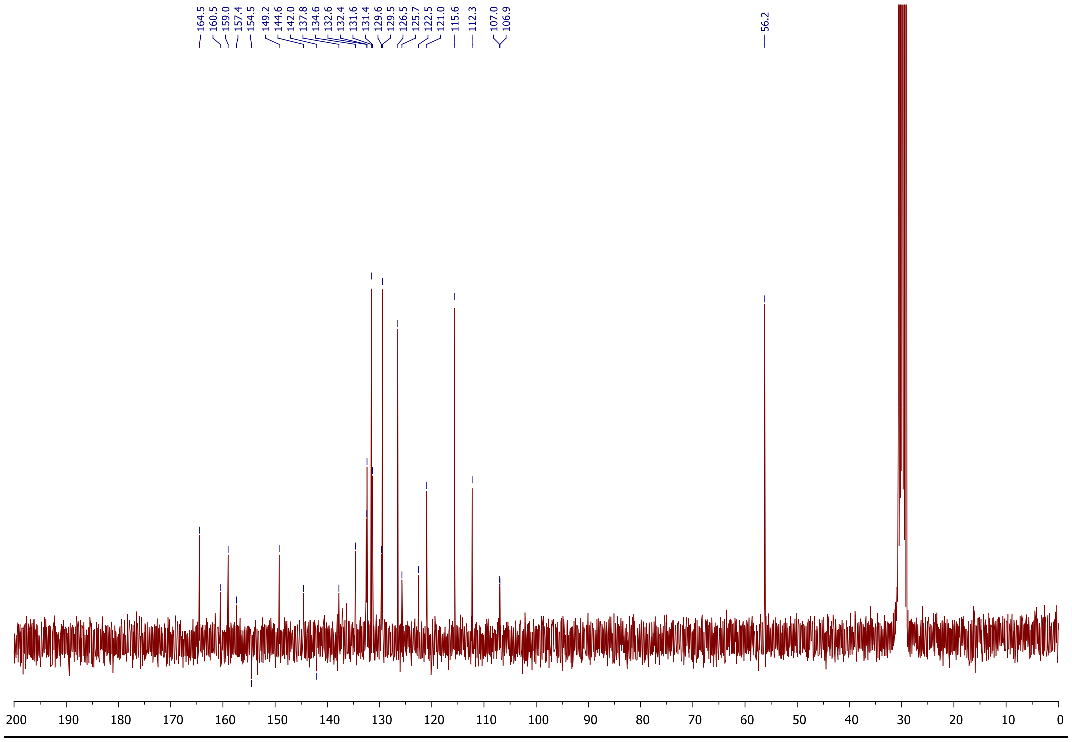

^{1}H-NMR (300 MHz, $CDCl_3$) **6f**

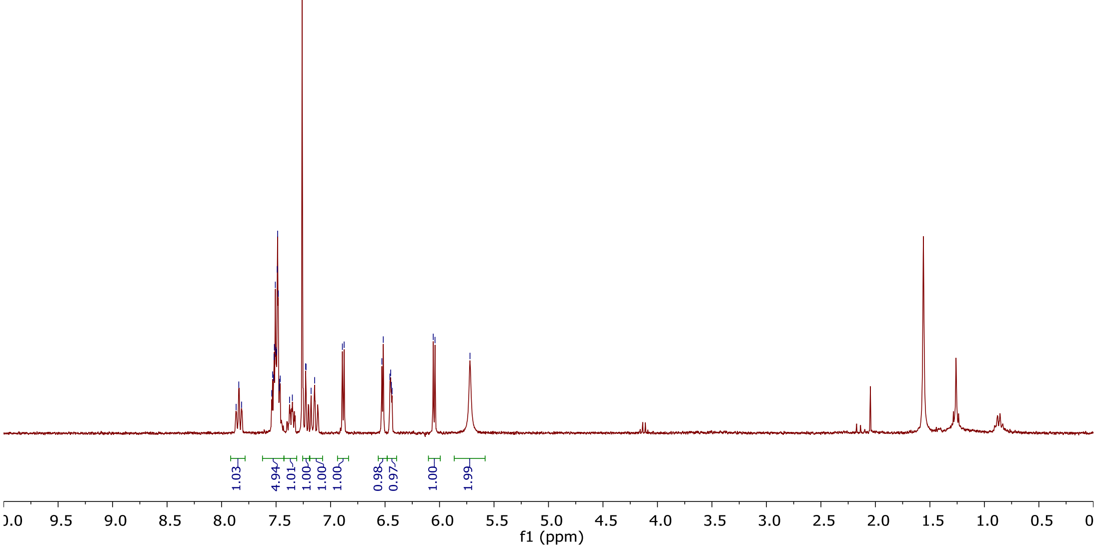

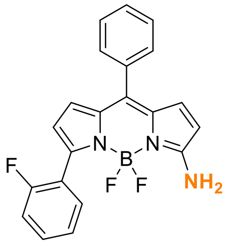

^{13}C-NMR (75 MHz, $CDCl_3$)

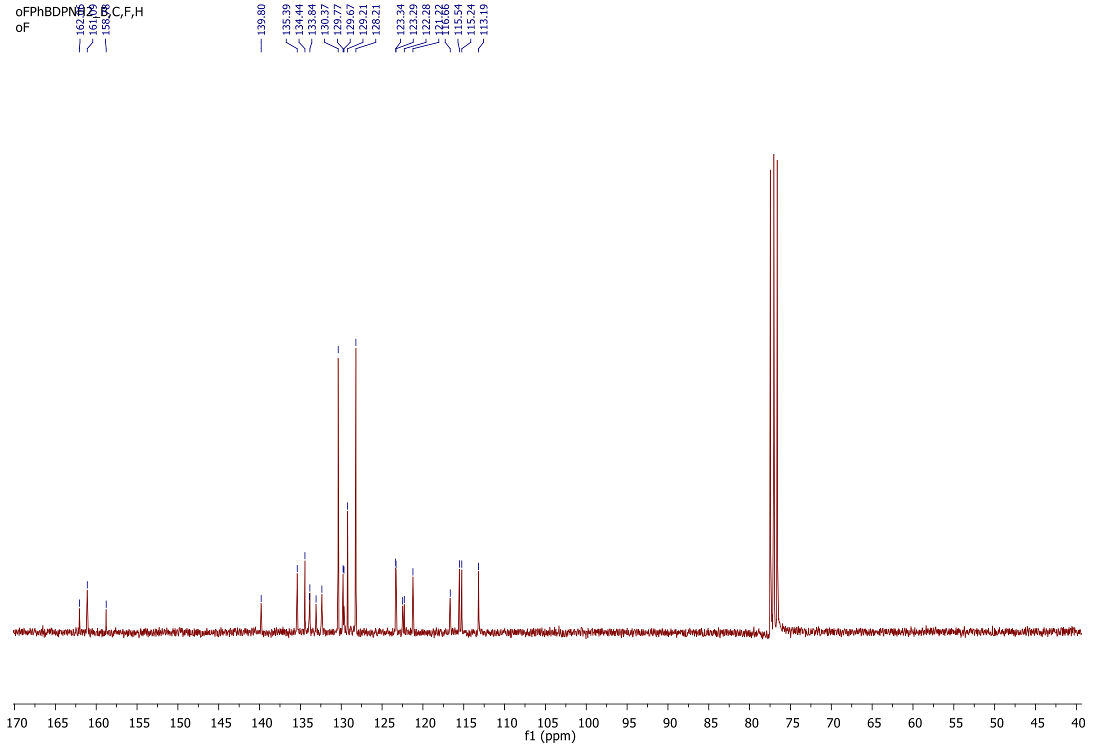

^{1}H-NMR (300 MHz, $CDCl_3$) **6g**

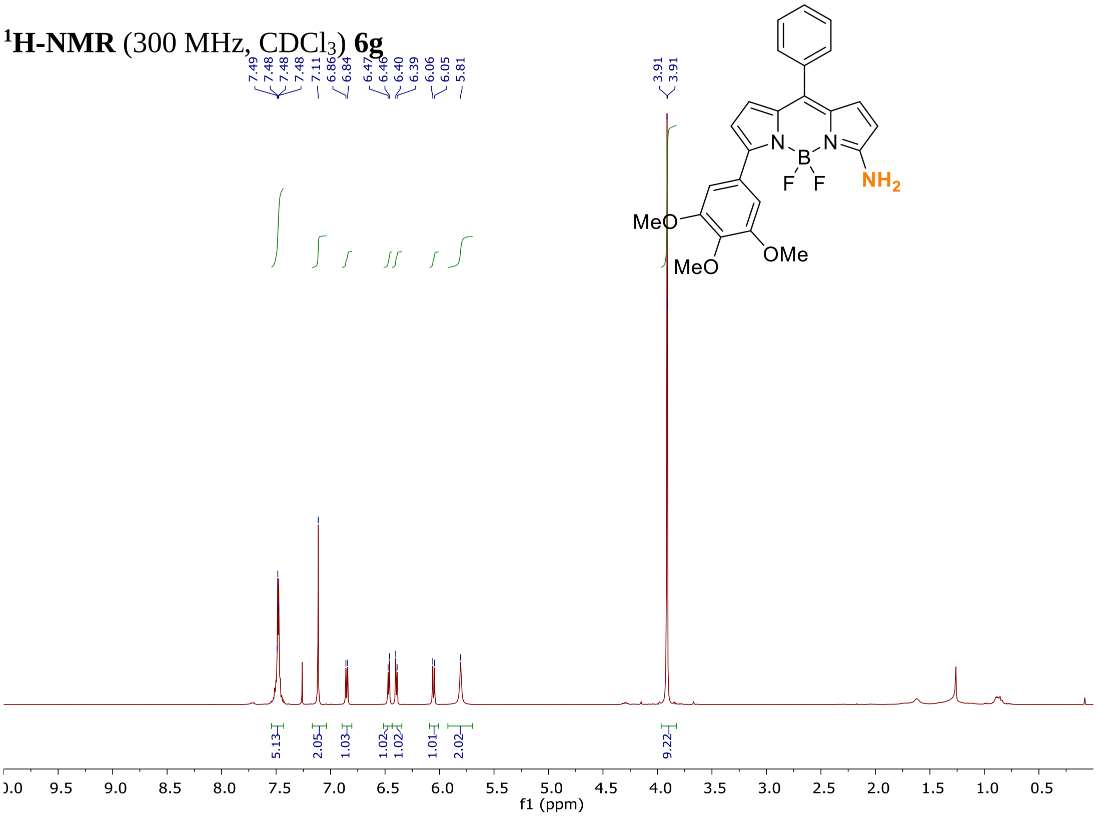

^{13}C-NMR (75 MHz, $CDCl_3$)

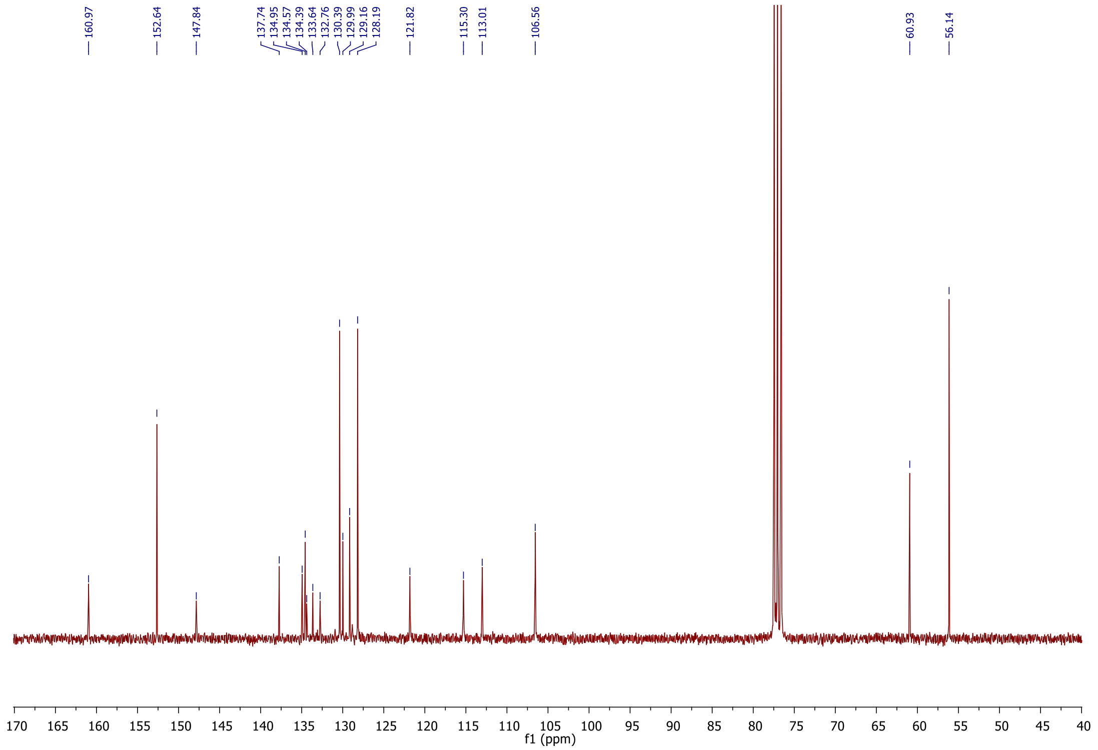

^{1}**H-NMR** (300 MHz, $CDCl_3$) **6h**

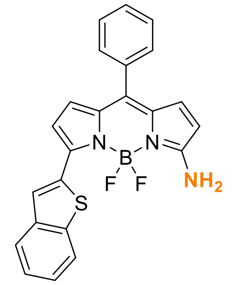

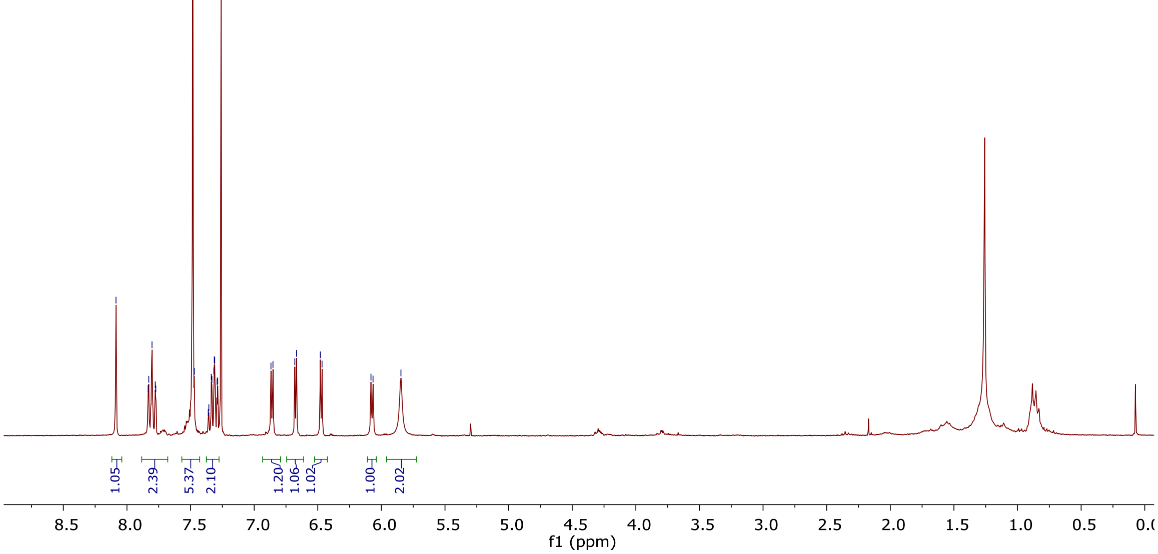

^{13}**C-NMR** (75 MHz, $CDCl_3$)

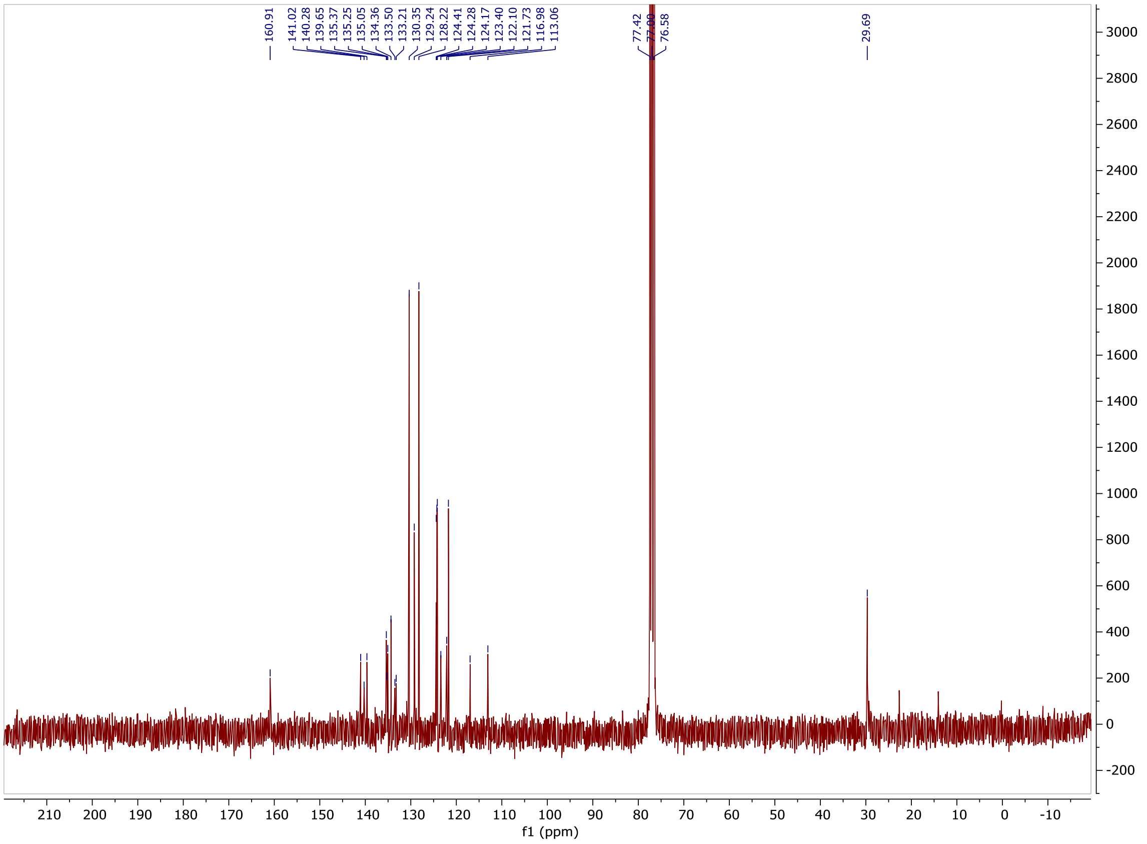

^{1}H-NMR (300 MHz, $CDCl_3$) **6i**

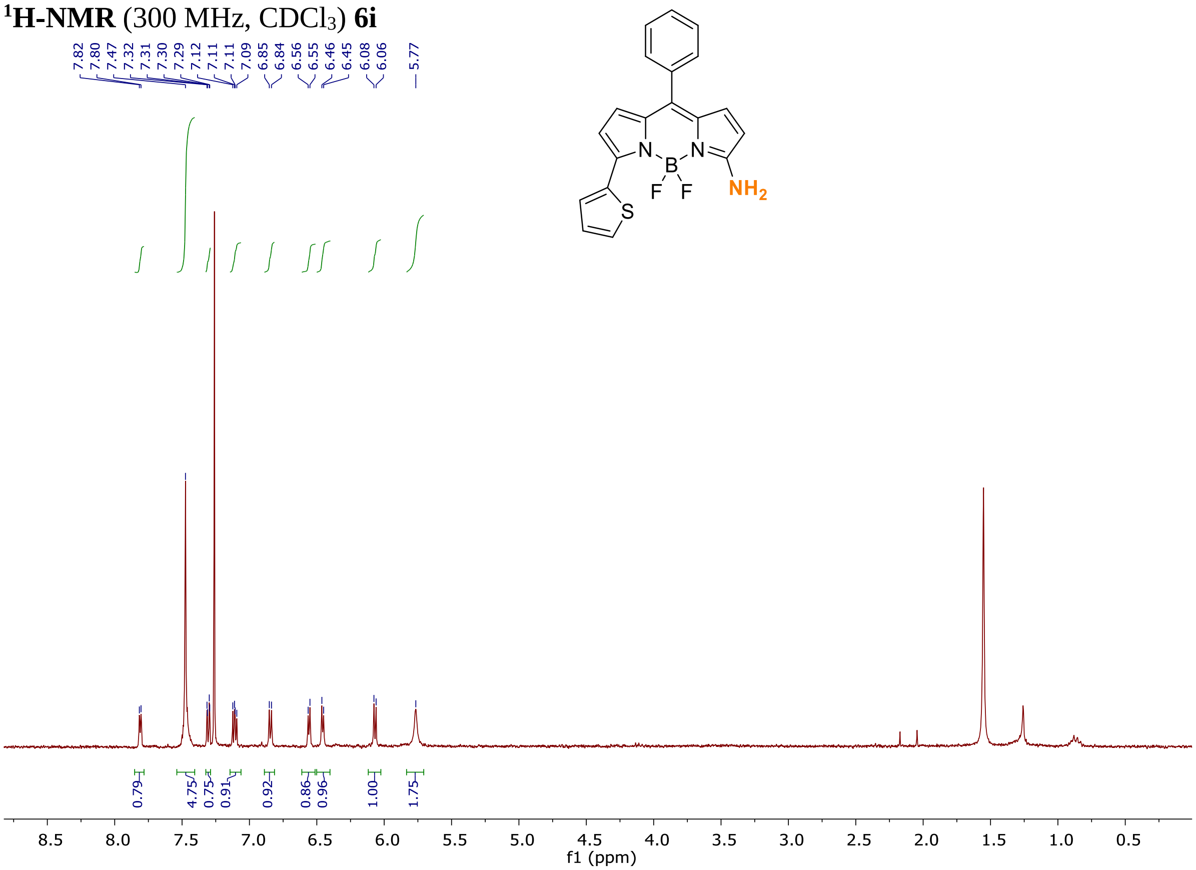

^{13}C-NMR (75 MHz, $CDCl_3$)

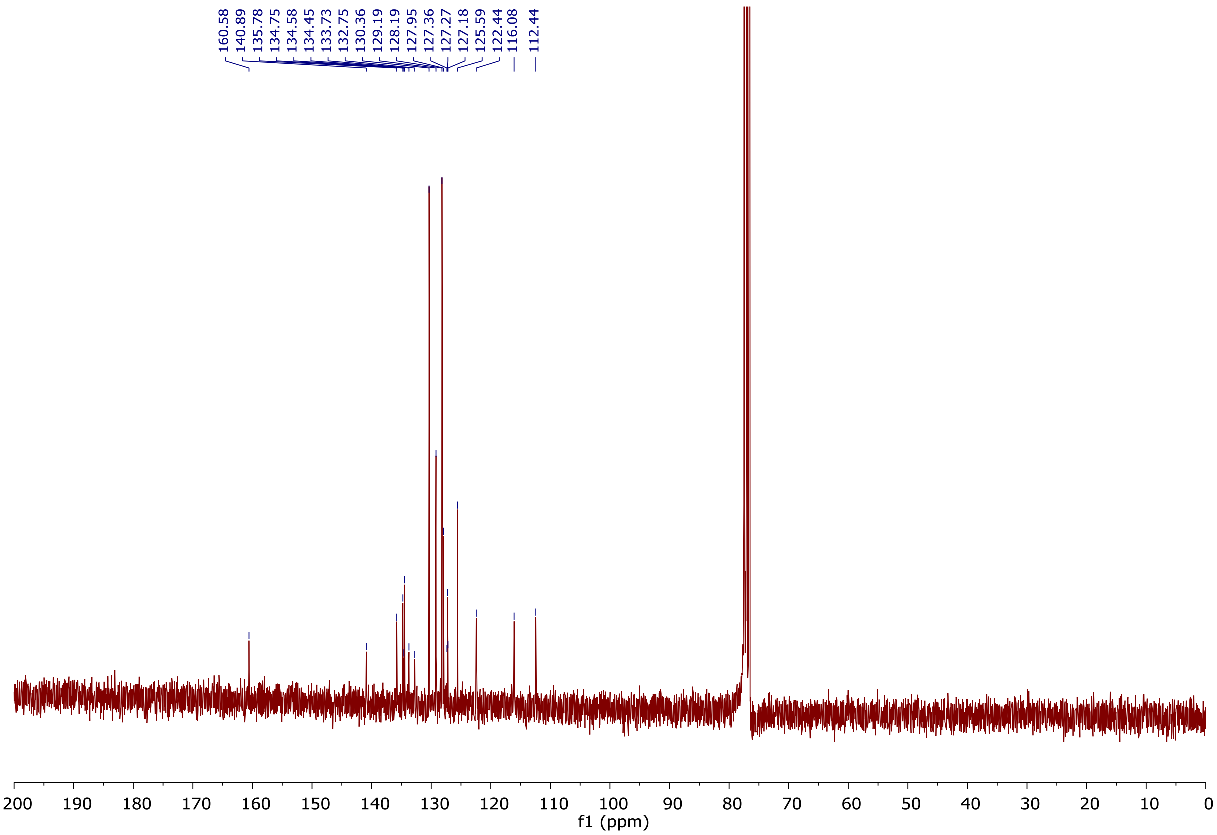

REFERENCES

1 Dilek, Ö.; Bane S. L. Synthesis of boron dipyrromethene fluorescent probes for bioorthogonal labeling. *Tetrahedron. Lett*. **2008**, *49*, 1413-1416.

2 (a) Baruah, M.; Qin, W.; Basaric, N.; De Borggraeve, W. M.; Boens, N. BODIPY-Based Hydroxyaryl Derivatives as Fluorescent pH Probes. *J. Org. Chem*. **2005**, *70*, 4152-4157. (b) Rohand, T.; Baruah, M.; Quin, W. W.; Boens, N.; Dehaen, W. Functionalisation of fluorescent BODIPY dyes by nucleophilic substitution. *Chem. Commun.*,**2006**, 266-268.

3 Henton, D. R.; McCreery, R. L.; Swenton, J. S. Anodic oxidation of 1,4-dimethoxy aromatic compounds. A facile route to functionalized quinone bisketals. *J. Org. Chem*. **1980**, *45*, 368-378.

4 Rohand, T.; Dolusic, E.; Ngo T. H.; Maes, W.; Dehaen, W. Efficient synthesis of aryldipyrromethanes in water and their application in the synthesis of corroles and dipyrromethenes. *Arkivoc* **2007**, 307-324.

5 Valeur B.; Berberan-Santos, M. N. *Molecular Fluorescence. Principles and Applications*, 2nd ed., Wiley-VCH: Weinheim (Germany), **2012**.

6 a) Lakowicz, J. *Principles of Fluorescence Spectroscopy*, 3rd ed., Springer-Verlag: New York, **2006**. b) Gasa, T.; Spruell, J.; Dichtel, W.; Srensen, T.; Philp, D.; Stoddart J.; Kuzmič, P. *Chem. Eur. J.*, **2009**, *15*, 106–116.

7 Chai, D.; Head-Gordon, M. Long-range corrected hybrid density functionals with damped atom–atom dispersion corrections. *Phys. Chem. Chem. Phys.*, **2008**, *10*, 6615-6620.

8 Dunning, T. H.; Gaussian basis sets for use in correlated molecular calculations. I. The atoms boron through neon and hydrogen. *J. Chem. Phys.*, **1989,** *90*, 1007-1023.

9 Cancès, E.; Mennucci, B.; Tomasi, J. A new integral equation formalism for the polarizable continuum model: Theoretical background and applications to isotropic and anisotropic dielectrics. *J. Chem. Phys.*, **1997**, *107*, 3032-3041.

10 Mennucci, B.; Cancès, E.; Tomasi, J. Evaluation of Solvent Effects in Isotropic and Anisotropic Dielectrics and in Ionic Solutions with a Unified Integral Equation Method: Theoretical Bases, Computational Implementation, and Numerical Applications. *J. Phys. Chem. B*, **1997**, *101*, 10506-10517.

11 Cancès, E.; Mennucci, B. New applications of integral equations methods for solvation continuum models: ionic solutions and liquid crystals. *J. Math. Chem.*, **1998**, *23*, 309-326.

12 Frisch, M. J. et al. Gaussian 16, Revision C.01, Gaussian, *Inc., Wallingford CT*, **2016**.

13 Trofimov, A. B.; Schirmer, J. An efficient polarization propagator approach to valence electron excitation spectra. *J. Phys. B At. Mol. Opt. Phys.*, **1995**, *28*, 2299-2324.

14 Schirmer, J. Beyond the random-phase approximation: A new approximation scheme for the polarization propagator. *Phys. Rev. A*, **1982,** *26*, 2395-2416.

15 Hättig, C.; Weigend F. CC2 excitation energy calculations on large molecules using the resolution of the identity approximation. *J. Chem. Phys.*, **2000**, *113*, 5154-5161.

16 Hättig, C.; Köhn, A. Transition moments and excited-state first-order properties in the coupled-cluster model CC2 using the resolution-of-the-identity approximation. *J. Chem. Phys.*, **2002**, *117*, 6939-6951.

17 Weigend, F.; Häser, M.; Patzelt, H.; Ahlrichs, R. RI-MP2: optimized auxiliary basis sets and demonstration of efficiency. *Chem. Phys. Lett.*, **1998**, *294*, 143-152.

18 Schäfer, A.; Horn, H.; Ahlrichs, R. Fully optimized contracted Gaussian basis sets for atoms Li to Kr. *J. Chem. Phys.*, **1992**, *97*, 2571-2577.

19 Balasubramani, S. G.; Chen, G. P.; Coriani, S.; Diedenhofen, M.; Frank, M. S.; Franzke, Y. J.; Furche, F.; Grotjahn, R.; Harding, M. E.; Hättig, C.; Hellweg, A.; Helmich-Paris, B.; Holzer, C.; Huniar, U.; Kaupp, M.; Marefat Khah, A.; Karbalaei Khani, S.; Müller, T.; Mack, F.; Nguyen, B. D.; Parker, S. M.; Perlt, E.; Rappoport, D.; Reiter, K.; Roy, S.; Rückert, M.; Schmitz, G.; Sierka, M.; Tapavicza, E.; Tew, D. P.; Van Wüllen, C.; Voora, V. K.; Weigend, F.; Wodyński, A.; Yu, J. M. TURBOMOLE: Modular program suite for ab initio quantum-chemical and condensed matter simulations. *J. Chem. Phys.*, **2020**, *152*, 184107-184136.

20 Plasser, F. *J. Chem. Phys.*, 2020, **152**, 084108-084114.

21 Mai, S.; Richter, M.; Heindl, M.; Menger, M. F. S. J.; Atkins, A.; Ruckenbauer, M.; Plasser, F.; Ibele, L.M.; Kropf, S.; Oppel, M.; Marquetand, P.; González, L. SHARC2.1: Surface Hopping Including Arbitrary Couplings – Program Package for Non-Adiabatic Dynamics, sharc-md.org, **2019**.

22 Richter, M.; Marquetan, P.; González-Vázquez, J.; Sola, I.; González, L. SHARC: ab Initio Molecular Dynamics with Surface Hopping in the Adiabatic Representation Including Arbitrary Couplings. *J. Chem. Theory Comput.* **2011**, *7*, 1253-1258.

23 Neese, F. The ORCA program system. *WIREs Comput. Mol. Sci.* **2012**, *2*, 73-78.

24 Fernandez-Galván I. et al. OpenMolcas: From Source Code to Insight. *J. Chem. Theory Comput*., **2019**, *15*, 5925-5964.

25 (a) Lind, C.; Cadenas, E.; Hochstein, P.; Ernster, L. D. T-diaphorase: purification, properties, and function. *Methods Enzymol.* 1990, **186** (C), 287-301. (b) Ross, D.; Siegel, D. NAD(P)H:quinone oxidoreductase 1 (NQO1, DT-diaphorase), functions and pharmacogenetics. *Methods Enzymol.* **2004**, *382*, 115-144. (c) Ross, D.; Kepa, J. K.; Winski, S. L.; Beall, H. D.; Anwar, A.; Siegel, D. NAD(P)H:quinone oxidoreductase 1 (NQO1): chemoprotection, bioactivation, gene regulation and genetic polymorphisms. *Chem. Biol. Interact*., **2000**, *129*, 77-97.

26 Riss, T. L.; Moravec, R. A.; Niles, A. L.; Duellman, S.; Benink, H. A.; Worzella, T. J.; Minor, L. *Cell viability assays Assay Guidance Manual Bethesda*: Eli Lilly & Company and the National Center for Advancing Translational Sciences; **2016**: 355-386.
